# Complete tangent stiffness matrix considering higher-order terms in the strain tensor and large rotations for a Euler Bernoulli - Timoshenko space beam-column element

**DOI:** 10.1016/j.mex.2021.101591

**Published:** 2021-11-24

**Authors:** Marcos Antonio Campos Rodrigues, Rodrigo Bird Burgos, Luiz Fernando Martha

**Affiliations:** a Federal University of Espírito Santo, Department of Civil Engineering, Avenida Fernando Ferrari, 514, Goiabeiras, Vitória, ES 29075-910, Brazil; b State University of Rio de Janeiro, Department of Structures and Foundations, Rua São Francisco Xavier, 524, Maracanã, Rio de Janeiro, RJ 20550-900, Brazil; c Pontifical Catholic University of Rio de Janeiro, Department of Civil Engineering, Rua Marques de São Vicente, 225, Gávea, Rio de Janeiro, RJ 22451-900, Brazil

**Keywords:** Tangent stiffness matrix, Analytical interpolation functions, Higher-order terms in strain tensor, Large rotations

## Abstract

This paper presents a unified method developed by Rodrigues et al. [1] to obtain a complete tangent stiffness matrix for spatial geometric nonlinear analysis using minimal discretization. The formulation presents four distinct important aspects to a complete analysis: interpolation (shape) functions, higher-order terms in the strain tensor and in the finite rotations, an updated Lagrangian kinematic description, and shear deformation effect (Timoshenko beam theory). Thus, the tangent stiffness matrix is calculated from the differential equation solution of deformed infinitesimal element equilibrium, considering the axial load and the shear deformation in this relation. This solution provides interpolation functions that are used in an updated Lagrangian formulation to construct the spatial tangent stiffness matrix considering higher-order terms in the strain tensor and in the finite rotations. The method provides an efficient formulation to perform geometric nonlinear analyses and predict the critical buckling load for spatial structures with moderate slenderness and with the interaction between axial and torsion effects, considering just one element in each member or a reduced discretization.

• Complete expressions for a geometric nonlinear analyses considering one element per member
• Spatial analyses considering higher-order terms in the strain tensor and large rotations
• Shear deformation influence included

## Graphical abstract

**Figure:**
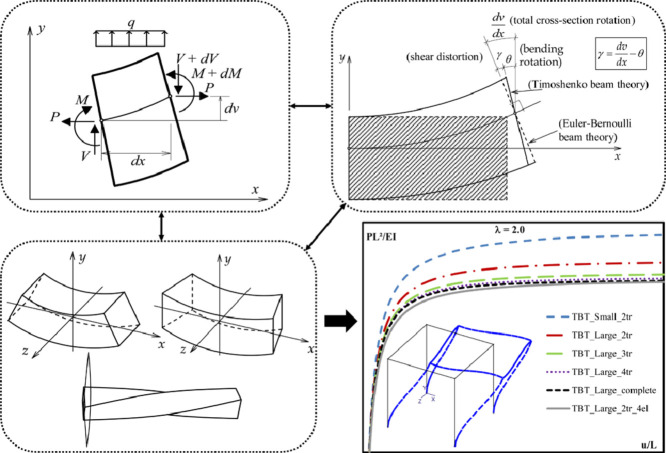

Specifications Table

**Table:** 

Subject Area:	Engineering
More specific subject area:	*Structural Nonlinear Analysis*
Method name:	*CEGNL - Complete Expressions for Geometric Nonlinear Analysis.*
Name and reference of original method:	*D. C. Chen, Geometric nonlinear analysis of three-dimensional structures, Cornell University, Ithaca, NY, 1994.* *W. Mcguire, R. H. Gallagher, R. D. Ziemian, Matrix structural analysis, John Wiley & Sons Inc, NY, USA, 2000.* *M. A. C. Rodrigues, R. B. Burgos,* L. *F. Martha, A unified approach to the Timoshenko geometric stiffness matrix considering higher-order terms in the strain tensor, Latin American Journal of Solids and Structures, 16 (2019) 1–22* *M. A. C. Rodrigues, R. B. Burgos,* L. *F. Martha, A unified approach to the Timoshenko 3d beam-column element tangent stiffness matrix considering higher-order terms in the strain tensor and large rotations, International Journal of Solids and Structures (2021),*10.1016/j.ijsolstr.2021.02.014
Resource availability:	https://gitlab.com/marcos.a.rodrigues/cenlg-complete-expressions-for-geometric-non-linear-analysis https://www.mathworks.com/matlabcentral/fileexchange/77380-cegnl-complete-expressions-for-geometric-non-linear-analys

## Method details

In the finite elements method (FEM), the analytical behavior of a solid can be approximated by a discrete behavior. The discrete solution is usually obtained by nodal displacements, while the continuous problem solution can be found by interpolating the nodal displacements using interpolating (shape) functions [2]. However, in general, the discrete solution using FEM introduces simplifications to the analytical mathematical idealization for the structure behavior, since the interpolation functions that define the deformed configuration of a structure are not compatible with the mathematical idealization for the response of the continuous medium [3]. An exception is for a linear analysis with constant cross-section elements, in which the cubic interpolation functions (the so-called Hermitian functions) represent the mathematical idealization of the structure behavior. However, in geometric nonlinear analysis, these cubic interpolation functions do not represent the analytical response.

Therefore, in geometric nonlinear analysis, to converge to the analytical behavior, a refined discretization or the use of a high-order finite element with an increase in the order of the polynomial of the basis functions is necessary. However, when the shape functions are obtained from the differential equation solution of the problem, the frame member discretization could be reduced [4], [5], [6], [7]. In fact, in this case, the continuous behavior of the element is represented by nodal parameters without the consideration of any other approximation.

The differential equation that describes a linear problem is obtained from the equilibrium of an infinitesimal element in an undeformed configuration, leading to the Hermitian interpolation functions. However, for geometric nonlinear or second-order analysis, the equilibrium of an infinitesimal element in the deformed configuration should be considered, and the differential equation considers the nonlinearity caused by the axial load in the deformed infinitesimal element [8].

Furthermore, to implement the unified method proposed by Rodrigues et al. [1], other important effects must be considered. The influence of shear deformation in beam-columns with a moderate slenderness ratio or with a small shear to bending ratio is well known. Indeed, employing the Timoshenko beam theory (TBT) in these cases provides better results than considering the Euler Bernoulli beam theory (EBBT) [9], [10]. Moreover, all expressions for the Timoshenko beam theory can be presented in a similar way to the corresponding expressions for Euler Bernoulli beam theory, differing only by auxiliary parameters that multiply the terms of the expressions. Thus the interpolation functions and the tangent stiffness matrix of a nonlinear analysis can be developed to consider either the Timoshenko or the Euler-Bernoulli beam theory, by making just a small parameter change.

In addition, to reach the expressions presented in this paper, an updated Lagrangian kinematic description considering spatial elements is used. Besides, the stiffness matrix is adjusted to consider finite rotation effects. Furthermore, the formulation employs the Green strain tensor considering all higher-order terms, which leads to accurate results and reduces the buckling load of spatial frame structures, showing that not considering these effects can overestimate the resistance of a structure.

The presented method combines three types of nonlinearities, namely, higher-order terms in the Green strain tensor, higher-order terms in the finite rotations, and the nonlinearity induced by the equilibrium of the infinitesimal element in the deformed configuration considering the axial effect. One main difference of the present formulation is that the interpolation functions for displacements and rotations in an element are directly obtained from the solution of differential equations that consider the equilibrium in the deformed configuration. As a consequence, the resulting interpolation functions depend on the axial force in the element. In addition, the present formulation considers the shear deformation influence, and expressions are developed in 3D. These considerations extend the classical tangent stiffness matrix for geometric nonlinear analysis [2,[11], [12], [13], [14]], as explained in the sequel.

The manuscript provides explicit expressions for the proposed interpolation functions and tangent stiffness matrix. Since this formulation involves trigonometric and hyperbolic functions, for the sake of computational efficiency the tangent stiffness matrix can be conveniently rewritten in polynomial functions using a Taylor series expansion. The manuscript shows expressions for expansions using two, three, and four terms. The paper in which the formulation is deduced [1] demonstrates that the tangent stiffness matrix with four terms provides accurate results when compared to the original matrix based on trigonometric and hyperbolic functions. MATLAB and C open-source codes with expressions presented in this paper are available in the GitLab [15] repository and in the MathWorks file exchange [16].

## Matrix approach to geometric nonlinear analysis

In a second-order elastic analysis, the structure behavior can be stated according to the equation $\left[K_{t}\right]\left\{d\Delta \right\}=\left\{dP\right\}$, where {dΔ} is a vector of incremental nodal displacements, {dP} is a vector of incremental nodal loads and reactions, and $\left[K_{t}\right]$ is a tangent stiffness matrix [2]. Usually, this matrix is composed by two components, $\left[K_{t}\right]=\left[K_{e}+\;K_{g}\right]$, a linear elastic component, $\left[K_{e}\right]$, and a nonlinear, $\left[K_{g}\right]$, the geometric stiffness matrix. The matrix can be developed in different ways and members of the structure need to be divided into several elements to provide satisfactory results. Also, the nonlinearity included in the matrix depends on distinct aspects, as the interpolation functions, the kinematic description, and the strain-displacement relations. Moreover, the matrix considers shear deformations and the expressions depend on the so-called Timoshenko constantΩ.

The conventional formulation of the tangent stiffness matrix is obtained from the virtual work principle considering an updated Lagrangian description using cubic (Hermitian) functions and disregard higher-order terms in the strain tensor and shear deformations (Euler-Bernoulli beam theory). However, in a geometric nonlinear analysis context, this discrete solution introduces simplifications to the mathematical idealization for the structure behavior, since these interpolation functions are not compatible with the mathematical idealization for the response of the continuous medium. Additionally, according to [10] consider higher-order terms in the strain tensor leads to smaller buckling loads, and, to predict the behavior of a beam-column with moderate slenderness, or with a small shear-to-bending rigidity ratio, the shear deformation has important effects, and in this case, the Timoshenko beam theory (TBT) provides better results.

Therefore, the method presented in this paper is for geometric nonlinear analysis and consists of calculating the tangent stiffness matrix of a 3D frame finite element, $\left[K_{t}\right]$, according to the expressions presented below. A structure member (beam or column) can be modeled considering a reduced, or even a minimal, discretization, in which each member corresponds to just one finite element. The formulation is also capable to evaluate structures considering the Timoshenko beam theory.

## Tangent stiffness matrix [K]

Theoretical concepts of the matrix formulated are developed in [1]. The authors explain that an exponential solution can be used, or, as an alternative, hyperbolic expressions can be used in cases of tensile forces, positive axial loads (*P* > 0), and trigonometric expressions in cases of compressive forces, negative axial loads (*P* < 0). The separation between tension and compression can be an additional conditional statement that can influence the efficiency of the code. This separation is not necessary if the implementation works directly with exponential functions [8,17]. The method proposed in this article employs hyperbolic and trigonometric functions because the tangent stiffness matrix is simpler. The steps necessary to use the method are postulated:

1 – Construction of elastic component of tangent stiffness matrix: $\left[K_{e}\right]$
2 –Construction of geometric component of tangent stiffness matrix: $\left[K_{g}\right]$
3 – Construction of axial and torsion interaction matrix: $\left[K_{int}\right]$
4 – Tangent stiffness matrix calculation: $\left[K\right]=\left[K_{e}\right]+\left[K_{g}\right]+\left[K_{\mathrm{int}}\right]$

Despite $\left[K_{e}\right]$ being called an elastic component, the matrix has nonlinear behavior, because in its formulation the axial load influence exists in the interpolation functions. Indeed, this interpretation comes from the elastic component in the Green strain tensor that generates the matrix [17]. The matrices use the parameters μ,Λand the constantΩ, introduced by Reddy [18].

(1) $$\begin{array}{ccc} \mu_{y} & = & \sqrt{\frac{P}{EI_{z}}\;};\mu_{z}=\sqrt{\frac{P}{EI_{y}}\;};\;\Omega_{y}=\frac{EI_{z}}{GA_{y}}\frac{1}{L^{2}};\Omega_{z}=\frac{EI_{y}}{GA_{z}}\frac{1}{L^{2}};\Lambda_{y}=\frac{\mu_{y}}{\sqrt{1+\Omega_{y}{\mu_{y}}^{2}L^{2}}};\\ & & \Lambda_{z}=\frac{\mu_{z}}{\sqrt{1+\Omega_{z}{\mu_{z}}^{2}L^{2}}} \end{array}$$

Elastic component - $\left[K_{e}\right]$:

For a tensile force (*P* > 0), the elastic component of the tangent stiffness matrix can be written in terms of hyperbolic functions, and the coefficients become:

(2) $$\begin{array}{ccc} C_{y} & = & 2\left(\text{sinh}\left(L\Lambda_{y}\right)-L\Lambda_{y}\right)+\Omega_{y}\left(L^{3}{\Lambda_{y}}^{3}+L^{2}{\Lambda_{y}}^{2}\text{sinh}\left(L\Lambda_{y}\right)\right);\\ D_{y} & = & {\left[2\;L\;\Lambda_{y}\;\text{cosh}\left(\frac{L\;\Lambda_{y}}{2}\right)-4\;\text{sinh}\left(\frac{L\;\Lambda_{y}}{2}\right)+4\;L^{2}\;{\Lambda_{y}}^{2}\;\Omega_{y}\;\text{sinh}\left(\frac{L\;\Lambda_{y}}{2}\right)\right]}^{2} \end{array}$$

(3) $$\begin{array}{c} C_{z}=2\left(\text{sinh}\left(L\Lambda_{z}\right)-L\Lambda_{z}\right)+\Omega_{z}\left(L^{3}{\Lambda_{z}}^{3}+L^{2}{\Lambda_{z}}^{2}\text{sinh}\left(L\Lambda_{z}\right)\right);\\ D_{z}\;={\left[2\;L\;\Lambda_{z}\;\text{cosh}\left(\frac{L\;\Lambda_{z}}{2}\right)-4\;\text{sinh}\left(\frac{L\;\Lambda_{z}}{2}\right)+4\;L^{2}\;{\Lambda_{z}}^{2}\;\Omega_{z}\;\text{sinh}\left(\frac{L\;\Lambda_{z}}{2}\right)\right]}^{2} \end{array}$$

(4) $$\begin{array}{ccc} \phi_{y} & = & 4\text{sinh}{\left(L\Lambda_{y}\right)}^{2}-16\text{sinh}{\left(\frac{L\Lambda_{y}}{2}\right)}^{2}+4L\Lambda_{y}\text{sinh}\left(L\Lambda_{y}\right)+L^{2}{\Lambda_{y}}^{2}\text{sinh}{\left(L\Lambda_{y}\right)}^{2}\\ & & +4L^{4}{\Lambda_{y}}^{4}{\Omega_{y}}^{2}\text{sinh}{\left(L\Lambda_{y}\right)}^{2}-16L^{4}{\Lambda_{y}}^{4}{\Omega_{y}}^{2}\text{sinh}{\left(\frac{L\Lambda_{y}}{2}\right)}^{2}\\ & & -4L^{3}{\Lambda_{y}}^{3}\Omega_{y}\text{sinh}\left(L\Lambda_{y}\right)-4L\Lambda_{y}\text{cosh}\left(L\Lambda_{y}\right)\text{sinh}\left(L\Lambda_{y}\right)-8L^{2}{\Lambda_{y}}^{2}\Omega_{y}\text{sinh}{\left(L\Lambda_{y}\right)}^{2}\\ & & +32L^{2}{\Lambda_{y}}^{2}\Omega_{y}\text{sinh}{\left(\frac{L\Lambda_{y}}{2}\right)}^{2}+4L^{3}{\Lambda_{y}}^{3}\Omega_{y}\text{cosh}\left(L\Lambda_{y}\right)\text{sinh}\left(L\Lambda_{y}\right) \end{array}$$

(5) $$\begin{array}{ccc} \phi_{z} & = & 4\text{sinh}{\left(L\Lambda_{z}\right)}^{2}-16\text{sinh}{\left(\frac{L\Lambda_{z}}{2}\right)}^{2}+4L\Lambda_{z}\text{sinh}\left(L\Lambda_{z}\right)+L^{2}{\Lambda_{z}}^{2}\text{sinh}{\left(L\Lambda_{z}\right)}^{2}\\ & & +4L^{4}{\Lambda_{z}}^{4}{\Omega_{z}}^{2}\text{sinh}{\left(L\Lambda_{z}\right)}^{2}-16L^{4}{\Lambda_{z}}^{4}{\Omega_{z}}^{2}\text{sinh}{\left(\frac{L\Lambda_{z}}{2}\right)}^{2}\\ & & -4L^{3}{\Lambda_{z}}^{3}\Omega_{z}\text{sinh}\left(L\Lambda_{z}\right)-4L\Lambda_{z}\text{cosh}\left(L\Lambda_{z}\right)\text{sinh}\left(L\Lambda_{z}\right)-8L^{2}{\Lambda_{z}}^{2}\Omega_{z}\text{sinh}{\left(L\Lambda_{z}\right)}^{2}\\ & & +32L^{2}{\Lambda_{z}}^{2}\Omega_{z}\text{sinh}{\left(\frac{L\Lambda_{z}}{2}\right)}^{2}+4L^{3}{\Lambda_{z}}^{3}\Omega_{z}\text{cosh}\left(L\Lambda_{z}\right)\text{sinh}\left(L\Lambda_{z}\right) \end{array}$$

(6) $$\begin{array}{ccc} B_{y} & = & L^{3}{\Lambda_{y}}^{3}-2\text{sinh}\left(L\Lambda_{y}\right)+2\text{cosh}\left(L\Lambda_{y}\right)\text{sinh}\left(L\Lambda_{y}\right)-L^{5}{\Lambda_{y}}^{5}\Omega_{y}\\ & & -2L\Lambda_{y}\text{sinh}{\left(L\Lambda_{y}\right)}^{2}-2L^{2}{\Lambda_{y}}^{2}\text{sinh}\left(L\Lambda_{y}\right)+4L\Lambda_{y}\text{sinh}{\left(\frac{L\Lambda_{y}}{2}\right)}^{2}+2L^{2}{\Lambda_{y}}^{2}\Omega_{y}\text{sinh}\left(L\Lambda_{y}\right)\\ & & +\;4L^{4}{\Lambda_{y}}^{4}\Omega_{y}\text{sinh}\left(L\Lambda_{y}\right)+L^{2}{\Lambda_{y}}^{2}\text{cosh}\left(L\Lambda_{y}\right)\text{sinh}\left(L\Lambda_{y}\right)+2L^{4}{\Lambda_{y}}^{4}{\Omega_{y}}^{2}\text{sinh}\left(L\Lambda_{y}\right)\\ & & -12L^{3}{\Lambda_{y}}^{3}\Omega_{y}\text{sinh}{\left(\frac{L\Lambda_{y}}{2}\right)}^{2}-2L^{6}{\Lambda_{y}}^{6}{\Omega_{y}}^{2}\text{sinh}\left(L\Lambda_{y}\right)-2L^{6}{\Lambda_{y}}^{6}{\Omega_{y}}^{3}\text{sinh}\left(L\Lambda_{y}\right)\\ & & +2L^{5}{\Lambda_{y}}^{5}{\Omega_{y}}^{2}\text{sinh}{\left(L\Lambda_{y}\right)}^{2}+12L^{5}{\Lambda_{y}}^{5}{\Omega_{y}}^{2}\text{sinh}{\left(\frac{L\Lambda_{y}}{2}\right)}^{2}-4L^{7}{\Lambda_{y}}^{7}{\Omega_{y}}^{3}\text{sinh}{\left(\frac{L\Lambda_{y}}{2}\right)}^{2}\\ & & -2L^{2}{\Lambda_{y}}^{2}\Omega_{y}\text{cosh}\left(L\Lambda_{y}\right)\text{sinh}\left(L\Lambda_{y}\right)+L^{4}{\Lambda_{y}}^{4}\Omega_{y}\text{cosh}\left(L\Lambda_{y}\right)\text{sinh}\left(L\Lambda_{y}\right)\\ & & -2L^{4}{\Lambda_{y}}^{4}{\Omega_{y}}^{2}\text{cosh}\left(L\Lambda_{y}\right)\text{sinh}\left(L\Lambda_{y}\right)+2L^{6}{\Lambda_{y}}^{6}{\Omega_{y}}^{3}\text{cosh}\left(L\Lambda_{y}\right)\text{sinh}\left(L\Lambda_{y}\right) \end{array}$$

(7) $$\begin{array}{ccc} B_{z} & = & L^{3}{\Lambda_{z}}^{3}-2\text{sinh}\left(L\Lambda_{z}\right)+2\text{cosh}\left(L\Lambda_{z}\right)\text{sinh}\left(L\Lambda_{z}\right)-L^{5}{\Lambda_{z}}^{5}\Omega_{z}\\ & & -2L\Lambda_{z}\text{sinh}{\left(L\Lambda_{z}\right)}^{2}-2L^{2}{\Lambda_{z}}^{2}\text{sinh}\left(L\Lambda_{z}\right)+4L\Lambda_{z}\text{sinh}{\left(\frac{L\Lambda_{z}}{2}\right)}^{2}+2L^{2}{\Lambda_{z}}^{2}\Omega_{z}\text{sinh}\left(L\Lambda_{z}\right)\\ & & +4L^{4}{\Lambda_{z}}^{4}\Omega_{z}\text{sinh}\left(L\Lambda_{z}\right)+L^{2}{\Lambda_{z}}^{2}\text{cosh}\left(L\Lambda_{z}\right)\text{sinh}\left(L\Lambda_{z}\right)+2L^{4}{\Lambda_{z}}^{4}{\Omega_{z}}^{2}\text{sinh}\left(L\Lambda_{z}\right)\\ & & -12L^{3}{\Lambda_{z}}^{3}\Omega_{z}\text{sinh}{\left(\frac{L\Lambda_{z}}{2}\right)}^{2}-2L^{6}{\Lambda_{z}}^{6}{\Omega_{z}}^{2}\text{sinh}\left(L\Lambda_{z}\right)-2L^{6}{\Lambda_{z}}^{6}{\Omega_{z}}^{3}\text{sinh}\left(L\Lambda_{z}\right)\\ & & +2L^{5}{\Lambda_{z}}^{5}{\Omega_{z}}^{2}\text{sinh}{\left(L\Lambda_{z}\right)}^{2}+12L^{5}{\Lambda_{z}}^{5}{\Omega_{z}}^{2}\text{sinh}{\left(\frac{L\Lambda_{z}}{2}\right)}^{2}-4L^{7}{\Lambda_{z}}^{7}{\Omega_{z}}^{3}\text{sinh}{\left(\frac{L\Lambda_{z}}{2}\right)}^{2}\\ & & -2L^{2}{\Lambda_{z}}^{2}\Omega_{z}\text{cosh}\left(L\Lambda_{z}\right)\text{sinh}\left(L\Lambda_{z}\right)+L^{4}{\Lambda_{z}}^{4}\Omega_{z}\text{cosh}\left(L\Lambda_{z}\right)\text{sinh}\left(L\Lambda_{z}\right)\\ & & -2L^{4}{\Lambda_{z}}^{4}{\Omega_{z}}^{2}\text{cosh}\left(L\Lambda_{z}\right)\text{sinh}\left(L\Lambda_{z}\right)+2L^{6}{\Lambda_{z}}^{6}{\Omega_{z}}^{3}\text{cosh}\left(L\Lambda_{z}\right)\text{sinh}\left(L\Lambda_{z}\right) \end{array}$$

(8) $$\begin{array}{ccc} F_{y} & = & 8L^{7}{\Lambda_{y}}^{7}{\Omega_{y}}^{3}\text{sinh}{\left(\frac{L\Lambda_{y}}{2}\right)}^{2}-8L^{6}{\Lambda_{y}}^{6}{\Omega_{y}}^{3}\text{sinh}\left(L\Lambda_{y}\right)\text{sinh}{\left(\frac{L\Lambda_{y}}{2}\right)}^{2}+4L^{6}{\Lambda_{y}}^{6}{\Omega_{y}}^{2}\text{sinh}\left(L\Lambda_{y}\right)\\ & & -4L^{5}{\Lambda_{y}}^{5}{\Omega_{y}}^{2}\text{sinh}{\left(L\Lambda_{y}\right)}^{2}-24L^{5}{\Lambda_{y}}^{5}{\Omega_{y}}^{2}\text{sinh}{\left(\frac{L\Lambda_{y}}{2}\right)}^{2}\\ & & +4L^{5}{\Lambda_{y}}^{5}\Omega_{y}\text{sinh}{\left(\frac{L\Lambda_{y}}{2}\right)}^{2}+2L^{5}{\Lambda_{y}}^{5}\Omega_{y}+8L^{4}{\Lambda_{y}}^{4}{\Omega_{y}}^{2}\text{sinh}\left(L\Lambda_{y}\right)\text{sinh}{\left(\frac{L\Lambda_{y}}{2}\right)}^{2}\\ & & -10L^{4}{\Lambda_{y}}^{4}\Omega_{y}\text{sinh}\left(L\Lambda_{y}\right)+24L^{3}{\Lambda_{y}}^{3}\Omega_{y}\text{sinh}{\left(\frac{L\Lambda_{y}}{2}\right)}^{2}-4L^{3}{\Lambda_{y}}^{3}\text{sinh}{\left(\frac{L\Lambda_{y}}{2}\right)}^{2}\\ & & -2L^{3}{\Lambda_{y}}^{3}+8L^{2}{\Lambda_{y}}^{2}\Omega_{y}\text{sinh}\left(L\Lambda_{y}\right)\text{sinh}{\left(\frac{L\Lambda_{y}}{2}\right)}^{2}+2L^{2}{\Lambda_{y}}^{2}\text{sinh}\left(L\Lambda_{y}\right)+4L\Lambda_{y}\text{sinh}{\left(L\Lambda_{y}\right)}^{2}\\ & & -8L\Lambda_{y}\text{sinh}{\left(\frac{L\Lambda_{y}}{2}\right)}^{2}-8\text{sinh}\left(L\Lambda_{y}\right)\text{sinh}{\left(\frac{L\Lambda_{y}}{2}\right)}^{2} \end{array}$$

(9) $$\begin{array}{ccc} F_{z} & = & 8L^{7}{\Lambda_{z}}^{7}{\Omega_{z}}^{3}\text{sinh}{\left(\frac{L\Lambda_{z}}{2}\right)}^{2}-8L^{6}{\Lambda_{z}}^{6}{\Omega_{z}}^{3}\text{sinh}\left(L\Lambda_{z}\right)\text{sinh}{\left(\frac{L\Lambda_{z}}{2}\right)}^{2}+4L^{6}{\Lambda_{z}}^{6}{\Omega_{z}}^{2}\text{sinh}\left(L\Lambda_{z}\right)\\ & & -\;4L^{5}{\Lambda_{z}}^{5}{\Omega_{z}}^{2}\text{sinh}{\left(L\Lambda_{z}\right)}^{2}-24L^{5}{\Lambda_{z}}^{5}{\Omega_{z}}^{2}\text{sinh}{\left(\frac{L\Lambda_{z}}{2}\right)}^{2}\\ & & +4L^{5}{\Lambda_{z}}^{5}\Omega_{z}\text{sinh}{\left(\frac{L\Lambda_{z}}{2}\right)}^{2}+2L^{5}{\Lambda_{z}}^{5}\Omega_{z}+8L^{4}{\Lambda_{z}}^{4}{\Omega_{z}}^{2}\text{sinh}\left(L\Lambda_{z}\right)\text{sinh}{\left(\frac{L\Lambda_{z}}{2}\right)}^{2}\\ & & -10L^{4}{\Lambda_{z}}^{4}\Omega_{z}\text{sinh}\left(L\Lambda_{z}\right)+24L^{3}{\Lambda_{z}}^{3}\Omega_{z}\text{sinh}{\left(\frac{L\Lambda_{z}}{2}\right)}^{2}-4L^{3}{\Lambda_{z}}^{3}\text{sinh}{\left(\frac{L\Lambda_{z}}{2}\right)}^{2}\\ & & -2L^{3}{\Lambda_{z}}^{3}+8L^{2}{\Lambda_{z}}^{2}\Omega_{z}\text{sinh}\left(L\Lambda_{z}\right)\text{sinh}{\left(\frac{L\Lambda_{z}}{2}\right)}^{2}+2L^{2}{\Lambda_{z}}^{2}\text{sinh}\left(L\Lambda_{z}\right)+4L\Lambda_{z}\text{sinh}{\left(L\Lambda_{z}\right)}^{2}\\ & & -8L\Lambda_{z}\text{sinh}{\left(\frac{L\Lambda_{z}}{2}\right)}^{2}-8\text{sinh}\left(L\Lambda_{z}\right)\text{sinh}{\left(\frac{L\Lambda_{z}}{2}\right)}^{2} \end{array}$$

For a compressive force (*P* < 0), the elastic component of the tangent stiffness matrix can be written in terms of trigonometric functions, and the coefficients become:

(10) $$\begin{array}{ccc} C_{y} & = & \left(\sin\left(L\Lambda_{y}\right)-L\Lambda_{y}-L^{3}{\Lambda_{y}}^{3}\Omega_{y}-L^{2}{\Lambda_{y}}^{2}\Omega_{y}\sin\left(L\Lambda_{y}\right)\right);\\ C_{z} & = & \left(\sin\left(L\Lambda_{z}\right)-L\Lambda_{z}-L^{3}{\Lambda_{z}}^{3}\Omega_{z}-L^{2}{\Lambda_{z}}^{2}\Omega_{z}\sin\left(L\Lambda_{z}\right)\right); \end{array}$$

(11) $$\begin{array}{ccc} D_{y}\; & = & 4\cos\left(L\Lambda_{y}\right)-L^{2}{\Lambda_{y}}^{2}-8L^{2}{\Lambda_{y}}^{2}\Omega_{y}-L^{2}{\Lambda_{y}}^{2}\cos\left(L\Lambda_{y}\right)+4L\Lambda_{y}\sin\left(L\Lambda_{y}\right)\\ & & -4L^{4}{\Lambda_{y}}^{4}{\Omega_{y}}^{2}+8L^{2}{\Lambda_{y}}^{2}\Omega_{y}\cos\left(L\Lambda_{y}\right)\\ & & +\;4L^{3}{\Lambda_{y}}^{3}\Omega_{y}\sin\left(L\Lambda_{y}\right)+4L^{4}{\Lambda_{y}}^{4}{\Omega_{y}}^{2}\cos\left(L\Lambda_{y}\right)-4 \end{array}$$

(12) $$\begin{array}{ccc} D_{z}\; & = & 4\cos\left(L\Lambda_{z}\right)-L^{2}{\Lambda_{z}}^{2}-8L^{2}{\Lambda_{z}}^{2}\Omega_{z}-L^{2}{\Lambda_{z}}^{2}\cos\left(L\Lambda_{z}\right)+4L\Lambda_{z}\sin\left(L\Lambda_{z}\right)\\ & & -\;4L^{4}{\Lambda_{z}}^{4}{\Omega_{z}}^{2}+8L^{2}{\Lambda_{z}}^{2}\Omega_{z}\cos\left(L\Lambda_{z}\right)\\ & & +4L^{3}{\Lambda_{z}}^{3}\Omega_{z}\sin\left(L\Lambda_{z}\right)+4L^{4}{\Lambda_{z}}^{4}{\Omega_{z}}^{2}\cos\left(L\Lambda_{z}\right)-4 \end{array}$$

(13) $$\begin{array}{ccc} \phi_{y} & = & 4\cos\left(2L\Lambda_{y}\right)-16\cos\left(L\Lambda_{y}\right)+L^{2}{\Lambda_{y}}^{2}+24L^{2}{\Lambda_{y}}^{2}\Omega_{y}-L^{2}{\Lambda_{y}}^{2}\cos\left(2L\Lambda_{y}\right)\\ & & -8L\Lambda_{y}\sin\left(L\Lambda_{y}\right)+4L\Lambda_{y}\sin\left(2L\Lambda_{y}\right)+12L^{4}{\Lambda_{y}}^{4}{\Omega_{y}}^{2}-32L^{2}{\Lambda_{y}}^{2}\Omega_{y}\cos\left(L\Lambda_{y}\right)\\ & & +8L^{2}{\Lambda_{y}}^{2}\Omega_{y}\cos\left(2L\Lambda_{y}\right)-8L^{3}{\Lambda_{y}}^{3}\Omega_{y}\sin\left(L\Lambda_{y}\right)+4L^{3}{\Lambda_{y}}^{3}\Omega_{y}\sin\left(2L\Lambda_{y}\right)\\ & & -16L^{4}{\Lambda_{y}}^{4}{\Omega_{y}}^{2}\cos\left(L\Lambda_{y}\right)+4L^{4}{\Lambda_{y}}^{4}{\Omega_{y}}^{2}\cos\left(2L\Lambda_{y}\right)+12 \end{array}$$

(14) $$\begin{array}{ccc} \phi_{z} & = & 4\cos\left(2L\Lambda_{z}\right)-16\cos\left(L\Lambda_{z}\right)+L^{2}{\Lambda_{z}}^{2}+24L^{2}{\Lambda_{z}}^{2}\Omega_{z}-L^{2}{\Lambda_{z}}^{2}\cos\left(2L\Lambda_{z}\right)\\ & & -8L\Lambda_{z}\sin\left(L\Lambda_{z}\right)+4L\Lambda_{z}\sin\left(2L\Lambda_{z}\right)+12L^{4}{\Lambda_{z}}^{4}{\Omega_{z}}^{2}-32L^{2}{\Lambda_{z}}^{2}\Omega_{z}\cos\left(L\Lambda_{z}\right)\\ & & +8L^{2}{\Lambda_{z}}^{2}\Omega_{z}\cos\left(2L\Lambda_{z}\right)-8L^{3}{\Lambda_{z}}^{3}\Omega_{z}\sin\left(L\Lambda_{z}\right)+4L^{3}{\Lambda_{z}}^{3}\Omega_{z}\sin\left(2L\Lambda_{z}\right)\\ & & -16L^{4}{\Lambda_{z}}^{4}{\Omega_{z}}^{2}\cos\left(L\Lambda_{z}\right)+4L^{4}{\Lambda_{z}}^{4}{\Omega_{z}}^{2}\cos\left(2L\Lambda_{z}\right)+12 \end{array}$$

(15) $$\begin{array}{ccc} B_{y} & = & 2(-\sin\left(2L\Lambda_{y}\right)+2\sin\left(L\Lambda_{y}\right)+L^{3}{\Lambda_{y}}^{3}+L\Lambda_{y}+6L^{3}{\Lambda_{y}}^{3}\Omega_{y}+L^{5}{\Lambda_{y}}^{5}\Omega_{y}\\ & & -2L^{2}{\Lambda_{y}}^{2}\sin\left(L\Lambda_{y}\right)+\frac{L^{2}{\Lambda_{y}}^{2}\sin\left(2L\Lambda_{y}\right)}{2}-2L\Lambda_{y}\cos\left(L\Lambda_{y}\right)+L\Lambda_{y}\cos\left(2L\Lambda_{y}\right)\\ & & +\;7L^{5}{\Lambda_{y}}^{5}{\Omega_{y}}^{2}+2L^{7}{\Lambda_{y}}^{7}{\Omega_{y}}^{3}-6L^{3}{\Lambda_{y}}^{3}\Omega_{y}\cos\left(L\Lambda_{y}\right)+2L^{2}{\Lambda_{y}}^{2}\Omega_{y}\sin\left(L\Lambda_{y}\right)\\ & & -\;L^{2}{\Lambda_{y}}^{2}\Omega_{y}\sin\left(2L\Lambda_{y}\right)-4L^{4}{\Lambda_{y}}^{4}\Omega_{y}\sin\left(L\Lambda_{y}\right)-\frac{L^{4}{\Lambda_{y}}^{4}\Omega_{y}\sin\left(2L\Lambda_{y}\right)}{2}\\ & & -6L^{5}{\Lambda_{y}}^{5}{\Omega_{y}}^{2}\cos\left(L\Lambda_{y}\right)-L^{5}{\Lambda_{y}}^{5}{\Omega_{y}}^{2}\cos\left(2L\Lambda_{y}\right)-2L^{7}{\Lambda_{y}}^{7}{\Omega_{y}}^{3}\cos\left(L\Lambda_{y}\right)\\ & & -2L^{4}{\Lambda_{y}}^{4}{\Omega_{y}}^{2}\sin\left(L\Lambda_{y}\right)+L^{4}{\Lambda_{y}}^{4}{\Omega_{y}}^{2}\sin\left(2L\Lambda_{y}\right)\\ & & -2L^{6}{\Lambda_{y}}^{6}{\Omega_{y}}^{2}\sin\left(L\Lambda_{y}\right)\\ & & -2L^{6}{\Lambda_{y}}^{6}{\Omega_{y}}^{3}\sin\left(L\Lambda_{y}\right)+L^{6}{\Lambda_{y}}^{6}{\Omega_{y}}^{3}\sin\left(2L\Lambda_{y}\right)) \end{array}$$

(16) $$\begin{array}{ccc} B_{z} & = & 2(-\sin\left(2L\Lambda_{z}\right)+2\sin\left(L\Lambda_{z}\right)+L^{3}{\Lambda_{z}}^{3}+L\Lambda_{z}+6L^{3}{\Lambda_{z}}^{3}\Omega_{z}+L^{5}{\Lambda_{z}}^{5}\Omega_{z}-2L^{2}{\Lambda_{z}}^{2}\sin\left(L\Lambda_{z}\right)\\ & & +\frac{L^{2}{\Lambda_{z}}^{2}\sin\left(2L\Lambda_{z}\right)}{2}-2L\Lambda_{z}\cos\left(L\Lambda_{z}\right)+L\Lambda_{z}\cos\left(2L\Lambda_{z}\right)\\ & & +7L^{5}{\Lambda_{z}}^{5}{\Omega_{z}}^{2}+2L^{7}{\Lambda_{z}}^{7}{\Omega_{z}}^{3}-6L^{3}{\Lambda_{z}}^{3}\Omega_{z}\cos\left(L\Lambda_{z}\right)+2L^{2}{\Lambda_{z}}^{2}\Omega_{z}\sin\left(L\Lambda_{z}\right)-L^{2}{\Lambda_{z}}^{2}\Omega_{z}\sin\left(2L\Lambda_{z}\right)\\ & & -4L^{4}{\Lambda_{z}}^{4}\Omega_{z}\sin\left(L\Lambda_{z}\right)-\frac{L^{4}{\Lambda_{z}}^{4}\Omega_{z}\sin\left(2L\Lambda_{z}\right)}{2}\\ & & -6L^{5}{\Lambda_{z}}^{5}{\Omega_{z}}^{2}\cos\left(L\Lambda_{z}\right)-L^{5}{\Lambda_{z}}^{5}{\Omega_{z}}^{2}\cos\left(2L\Lambda_{z}\right)-2L^{7}{\Lambda_{z}}^{7}{\Omega_{z}}^{3}\cos\left(L\Lambda_{z}\right)\\ & & -2L^{4}{\Lambda_{z}}^{4}{\Omega_{z}}^{2}\sin\left(L\Lambda_{z}\right)+L^{4}{\Lambda_{z}}^{4}{\Omega_{z}}^{2}\sin\left(2L\Lambda_{z}\right)-2L^{6}{\Lambda_{z}}^{6}{\Omega_{z}}^{2}\sin\left(L\Lambda_{z}\right)\\ & & -2L^{6}{\Lambda_{z}}^{6}{\Omega_{z}}^{3}\sin\left(L\Lambda_{z}\right)+L^{6}{\Lambda_{z}}^{6}{\Omega_{z}}^{3}\sin\left(2L\Lambda_{z}\right)) \end{array}$$

(17) $$\begin{array}{ccc} F_{y} & = & 4(\sin\left(2L\Lambda_{y}\right)-2\sin\left(L\Lambda_{y}\right)-L\Lambda_{y}-6L^{3}{\Lambda_{y}}^{3}\Omega_{y}-L^{3}{\Lambda_{y}}^{3}\cos\left(L\Lambda_{y}\right)\\ & & +L^{2}{\Lambda_{y}}^{2}\sin\left(L\Lambda_{y}\right)+2L\Lambda_{y}\cos\left(L\Lambda_{y}\right)-L\Lambda_{y}\cos\left(2L\Lambda_{y}\right)-7L^{5}{\Lambda_{y}}^{5}{\Omega_{y}}^{2}-2L^{7}{\Lambda_{y}}^{7}{\Omega_{y}}^{3}\\ & & +6L^{3}{\Lambda_{y}}^{3}\Omega_{y}\cos\left(L\Lambda_{y}\right)-L^{5}{\Lambda_{y}}^{5}\Omega_{y}\cos\left(L\Lambda_{y}\right)-2L^{2}{\Lambda_{y}}^{2}\Omega_{y}\sin\left(L\Lambda_{y}\right)\\ & & +L^{2}{\Lambda_{y}}^{2}\Omega_{y}\sin\left(2L\Lambda_{y}\right)+5L^{4}{\Lambda_{y}}^{4}\Omega_{y}\sin\left(L\Lambda_{y}\right)+6L^{5}{\Lambda_{y}}^{5}{\Omega_{y}}^{2}\cos\left(L\Lambda_{y}\right)\\ & & +L^{5}{\Lambda_{y}}^{5}{\Omega_{y}}^{2}\cos\left(2L\Lambda_{y}\right)+2L^{7}{\Lambda_{y}}^{7}{\Omega_{y}}^{3}\cos\left(L\Lambda_{y}\right)+2L^{4}{\Lambda_{y}}^{4}{\Omega_{y}}^{2}\sin\left(L\Lambda_{y}\right)\\ & & -\;L^{4}{\Lambda_{y}}^{4}{\Omega_{y}}^{2}\sin\left(2L\Lambda_{y}\right)+2L^{6}{\Lambda_{y}}^{6}{\Omega_{y}}^{2}\sin\left(L\Lambda_{y}\right)+2L^{6}{\Lambda_{y}}^{6}{\Omega_{y}}^{3}\sin\left(L\Lambda_{y}\right)\\ & & -L^{6}{\Lambda_{y}}^{6}{\Omega_{y}}^{3}\sin\left(2L\Lambda_{y}\right)) \end{array}$$

(18) $$\begin{array}{ccc} F_{z} & = & 4(\sin\left(2L\Lambda_{z}\right)-2\sin\left(L\Lambda_{z}\right)-L\Lambda_{z}-6L^{3}{\Lambda_{z}}^{3}\Omega_{z}-L^{3}{\Lambda_{z}}^{3}\cos\left(L\Lambda_{z}\right)\\ & & +L^{2}{\Lambda_{z}}^{2}\sin\left(L\Lambda_{z}\right)+2L\Lambda_{z}\cos\left(L\Lambda_{z}\right)-L\Lambda_{z}\cos\left(2L\Lambda_{z}\right)-7L^{5}{\Lambda_{z}}^{5}{\Omega_{z}}^{2}-2L^{7}{\Lambda_{z}}^{7}{\Omega_{z}}^{3}\\ & & +6L^{3}{\Lambda_{z}}^{3}\Omega_{z}\cos\left(L\Lambda_{z}\right)-L^{5}{\Lambda_{z}}^{5}\Omega_{z}\cos\left(L\Lambda_{z}\right)-2L^{2}{\Lambda_{z}}^{2}\Omega_{z}\sin\left(L\Lambda_{z}\right)\\ & & +L^{2}{\Lambda_{z}}^{2}\Omega_{z}\sin\left(2L\Lambda_{z}\right)+5L^{4}{\Lambda_{z}}^{4}\Omega_{z}\sin\left(L\Lambda_{z}\right)+6L^{5}{\Lambda_{z}}^{5}{\Omega_{z}}^{2}\cos\left(L\Lambda_{z}\right)\\ & & +L^{5}{\Lambda_{z}}^{5}{\Omega_{z}}^{2}\cos\left(2L\Lambda_{z}\right)+2L^{7}{\Lambda_{z}}^{7}{\Omega_{z}}^{3}\cos\left(L\Lambda_{z}\right)+2L^{4}{\Lambda_{z}}^{4}{\Omega_{z}}^{2}\sin\left(L\Lambda_{z}\right)\\ & & -L^{4}{\Lambda_{z}}^{4}{\Omega_{z}}^{2}\sin\left(2L\Lambda_{z}\right)+2L^{6}{\Lambda_{z}}^{6}{\Omega_{z}}^{2}\sin\left(L\Lambda_{z}\right)+2L^{6}{\Lambda_{z}}^{6}{\Omega_{z}}^{3}\sin\left(L\Lambda_{z}\right)\\ & & -L^{6}{\Lambda_{z}}^{6}{\Omega_{z}}^{3}\sin\left(2L\Lambda_{z}\right)) \end{array}$$

Geometric component - $\left[K_{g}\right]$:

For a tensile force (*P* > 0), the geometric component of the tangent stiffness matrix can be written in terms of hyperbolic functions, and the coefficients become:

(19) $$\begin{array}{c} D_{y}\;=\left(L\Lambda_{y}\text{sinh}\left(L\Lambda_{y}\right)-2L^{2}{\Lambda_{y}}^{2}\Omega_{y}-2\text{cosh}\left(L\Lambda_{y}\right)+2L^{2}{\Lambda_{y}}^{2}\Omega_{y}\text{cosh}\left(L\Lambda_{y}\right)+2\right);\\ C_{y}\;=\left(\text{cosh}\left(L\Lambda_{y}\right)-1\right)\left(\text{sinh}\left(L\Lambda_{y}\right)-L\Lambda_{y}\right) \end{array}$$

(20) $$\begin{array}{ccc} \alpha_{y}\; & = & \Lambda_{y}(3L\Lambda_{y}-3\text{sinh}\left(L\Lambda_{y}\right)-2L^{3}{\Lambda_{y}}^{3}\Omega_{y}+L^{5}{\Lambda_{y}}^{5}{\Omega_{y}}^{2}+2L\Lambda_{y}\text{sinh}{\left(\frac{L\Lambda_{y}}{2}\right)}^{2}\\ & & +2L^{2}{\Lambda_{y}}^{2}\Omega_{y}\text{sinh}\left(L\Lambda_{y}\right)+L^{4}{\Lambda_{y}}^{4}{\Omega_{y}}^{2}\text{sinh}\left(L\Lambda_{y}\right)); \end{array}$$

(21) $$\begin{array}{ccc} \beta_{y} & = & 2(L^{2}{\Lambda_{y}}^{2}+4\text{sinh}{\left(\frac{L\Lambda_{y}}{2}\right)}^{2}-2L\Lambda_{y}\text{sinh}\left(L\Lambda_{y}\right)+L^{2}{\Lambda_{y}}^{2}\text{sinh}{\left(\frac{L\Lambda_{y}}{2}\right)}^{2}\\ & & +\;4L^{4}{\Lambda_{y}}^{4}{\Omega_{y}}^{2}\text{sinh}{\left(\frac{L\Lambda_{y}}{2}\right)}^{2}+2L^{3}{\Lambda_{y}}^{3}\Omega_{y}\text{sinh}\left(L\Lambda_{y}\right)-8L^{2}{\Lambda_{y}}^{2}\Omega_{y}\text{sinh}{\left(\frac{L\Lambda_{y}}{2}\right)}^{2}) \end{array}$$

(22) $$\begin{array}{ccc} \phi_{y} & = & 4L^{4}{\Lambda_{y}}^{4}{\Omega_{y}}^{2}\text{sinh}{\left(L\Lambda_{y}\right)}^{2}-16L^{4}{\Lambda_{y}}^{4}{\Omega_{y}}^{2}\text{sinh}{\left(\frac{L\Lambda_{y}}{2}\right)}^{2}\\ & & +\;8L^{3}{\Lambda_{y}}^{3}\Omega_{y}\text{sinh}\left(L\Lambda_{y}\right)\text{sinh}{\left(\frac{L\Lambda_{y}}{2}\right)}^{2}-8L^{2}{\Lambda_{y}}^{2}\Omega_{y}\text{sinh}{\left(L\Lambda_{y}\right)}^{2}\\ & & +\;32L^{2}{\Lambda_{y}}^{2}\Omega_{y}\text{sinh}{\left(\frac{L\Lambda_{y}}{2}\right)}^{2}+L^{2}{\Lambda_{y}}^{2}\text{sinh}{\left(L\Lambda_{y}\right)}^{2}-8L\Lambda_{y}\text{sinh}\left(L\Lambda_{y}\right)\text{sinh}{\left(\frac{L\Lambda_{y}}{2}\right)}^{2}\\ & & +\;4\text{sinh}{\left(L\Lambda_{y}\right)}^{2}-16\text{sinh}{\left(\frac{L\Lambda_{y}}{2}\right)}^{2} \end{array}$$

(23) $$\begin{array}{c} \gamma_{y}\;={\left(L^{2}{\Lambda_{y}}^{2}\Omega_{y}-1\right)}^{2}\left(L^{2}{\Lambda_{y}}^{2}-8\text{sinh}{\left(\frac{L\Lambda_{y}}{2}\right)}^{2}+L\Lambda_{y}\text{sinh}\left(L\Lambda_{y}\right)\right);\\ \gamma_{z}\;={\left(L^{2}{\Lambda_{z}}^{2}\Omega_{z}-1\right)}^{2}\left(L^{2}{\Lambda_{z}}^{2}-8\text{sinh}{\left(\frac{L\Lambda_{z}}{2}\right)}^{2}+L\Lambda_{z}\text{sinh}\left(L\Lambda_{z}\right)\right) \end{array}$$

(24) $$\begin{array}{ccc} D_{z}\; & = & \left(L\Lambda_{z}\text{sinh}\left(L\Lambda_{z}\right)-2L^{2}{\Lambda_{z}}^{2}\Omega_{z}-2\text{cosh}\left(L\Lambda_{z}\right)+2L^{2}{\Lambda_{z}}^{2}\Omega_{z}\text{cosh}\left(L\Lambda_{z}\right)+2\right);\\ C_{z}\; & = & \left(\text{cosh}\left(L\Lambda_{z}\right)-1\right)\left(\text{sinh}\left(L\Lambda_{z}\right)-L\Lambda_{z}\right) \end{array}$$

(25) $$\begin{array}{ccc} \alpha_{z}\; & = & \Lambda_{z}(3L\Lambda_{z}-3\text{sinh}\left(L\Lambda_{z}\right)-2L^{3}{\Lambda_{z}}^{3}\Omega_{z}+L^{5}{\Lambda_{z}}^{5}{\Omega_{z}}^{2}+2L\Lambda_{z}\text{sinh}{\left(\frac{L\Lambda_{z}}{2}\right)}^{2}\\ & & +2L^{2}{\Lambda_{z}}^{2}\Omega_{y}\text{sinh}\left(L\Lambda_{z}\right)+L^{4}{\Lambda_{z}}^{4}{\Omega_{z}}^{2}\text{sinh}\left(L\Lambda_{z}\right)) \end{array}$$

(26) $$\begin{array}{ccc} \beta_{z} & = & 2(L^{2}{\Lambda_{z}}^{2}+4\text{sinh}{\left(\frac{L\Lambda_{z}}{2}\right)}^{2}-2L\Lambda_{z}\text{sinh}\left(L\Lambda_{z}\right)+L^{2}{\Lambda_{z}}^{2}\text{sinh}{\left(\frac{L\Lambda_{z}}{2}\right)}^{2}\\ & & +\;4L^{4}{\Lambda_{z}}^{4}{\Omega_{z}}^{2}\text{sinh}{\left(\frac{L\Lambda_{z}}{2}\right)}^{2}+2L^{3}{\Lambda_{z}}^{3}\Omega_{z}\text{sinh}\left(L\Lambda_{z}\right)\\ & & -\;8L^{2}{\Lambda_{z}}^{2}\Omega_{z}\text{sinh}{\left(\frac{L\Lambda_{z}}{2}\right)}^{2}) \end{array}$$

(27) $$\begin{array}{ccc} \phi_{z} & = & 4L^{4}{\Lambda_{z}}^{4}{\Omega_{z}}^{2}\text{sinh}{\left(L\Lambda_{z}\right)}^{2}-16L^{4}{\Lambda_{z}}^{4}{\Omega_{z}}^{2}\text{sinh}{\left(\frac{L\Lambda_{z}}{2}\right)}^{2}\\ & & +8L^{3}{\Lambda_{z}}^{3}\Omega_{z}\text{sinh}\left(L\Lambda_{z}\right)\text{sinh}{\left(\frac{L\Lambda_{z}}{2}\right)}^{2}-8L^{2}{\Lambda_{z}}^{2}\Omega_{z}\text{sinh}{\left(L\Lambda_{z}\right)}^{2}\\ & & +32L^{2}{\Lambda_{z}}^{2}\Omega_{z}\text{sinh}{\left(\frac{L\Lambda_{z}}{2}\right)}^{2}+L^{2}{\Lambda_{z}}^{2}\text{sinh}{\left(L\Lambda_{z}\right)}^{2}-8L\Lambda_{z}\text{sinh}\left(L\Lambda_{z}\right)\text{sinh}{\left(\frac{L\Lambda_{z}}{2}\right)}^{2}\\ & & +4\text{sinh}{\left(L\Lambda_{z}\right)}^{2}-16\text{sinh}{\left(\frac{L\Lambda_{z}}{2}\right)}^{2} \end{array}$$

(28) $$\begin{array}{ccc} \varphi_{y} & = & {\left(L^{2}{\Lambda_{y}}^{2}\Omega_{y}-1\right)}^{2}(-4L^{5}{\Lambda_{y}}^{5}{\Omega_{y}}^{2}\text{sinh}{\left(\frac{L\Lambda_{y}}{2}\right)}^{2}\\ & & +4L^{4}{\Lambda_{y}}^{4}{\Omega_{y}}^{2}\text{sinh}\left(L\Lambda_{y}\right)\text{sinh}{\left(\frac{L\Lambda_{y}}{2}\right)}^{2}-2L^{4}{\Lambda_{y}}^{4}\Omega_{y}\text{sinh}\left(L\Lambda_{y}\right)+2L^{3}{\Lambda_{y}}^{3}\Omega_{y}\text{sinh}{\left(L\Lambda_{y}\right)}^{2}\\ & & +\;8L^{3}{\Lambda_{y}}^{3}\Omega_{y}\text{sinh}{\left(\frac{L\Lambda_{y}}{2}\right)}^{2}-L^{3}{\Lambda_{y}}^{3}-8L^{2}{\Lambda_{y}}^{2}\Omega_{y}\text{sinh}\left(L\Lambda_{y}\right)\text{sinh}{\left(\frac{L\Lambda_{y}}{2}\right)}^{2}\\ & & +2L^{2}{\Lambda_{y}}^{2}\text{sinh}\left(L\Lambda_{y}\right)\text{sinh}{\left(\frac{L\Lambda_{y}}{2}\right)}^{2}+3L^{2}{\Lambda_{y}}^{2}\text{sinh}\left(L\Lambda_{y}\right)\\ & & -\;4L\Lambda_{y}\text{sinh}{\left(L\Lambda_{y}\right)}^{2}+4L\Lambda_{y}\text{sinh}{\left(\frac{L\Lambda_{y}}{2}\right)}^{2}+4\text{sinh}\left(L\Lambda_{y}\right)\text{sinh}{\left(\frac{L\Lambda_{y}}{2}\right)}^{2}) \end{array}$$

(29) $$\begin{array}{ccc} \varphi_{z} & = & {\left(L^{2}{\Lambda_{z}}^{2}\Omega_{z}-1\right)}^{2}(-4L^{5}{\Lambda_{z}}^{5}{\Omega_{z}}^{2}\text{sinh}{\left(\frac{L\Lambda_{z}}{2}\right)}^{2}+4L^{4}{\Lambda_{z}}^{4}{\Omega_{z}}^{2}\text{sinh}\left(L\Lambda_{z}\right)\text{sinh}{\left(\frac{L\Lambda_{z}}{2}\right)}^{2}\\ & & -\;2L^{4}{\Lambda_{z}}^{4}\Omega_{z}\text{sinh}\left(L\Lambda_{z}\right)+2L^{3}{\Lambda_{z}}^{3}\Omega_{z}\text{sinh}{\left(L\Lambda_{z}\right)}^{2}\\ & & +8L^{3}{\Lambda_{z}}^{3}\Omega_{z}\text{sinh}{\left(\frac{L\Lambda_{z}}{2}\right)}^{2}-L^{3}{\Lambda_{z}}^{3}-8L^{2}{\Lambda_{z}}^{2}\Omega_{z}\text{sinh}\left(L\Lambda_{z}\right)\text{sinh}{\left(\frac{L\Lambda_{z}}{2}\right)}^{2}\\ & & +2L^{2}{\Lambda_{z}}^{2}\text{sinh}\left(L\Lambda_{z}\right)\text{sinh}{\left(\frac{L\Lambda_{z}}{2}\right)}^{2}+3L^{2}{\Lambda_{z}}^{2}\text{sinh}\left(L\Lambda_{z}\right)\\ & & -4L\Lambda_{z}\text{sinh}{\left(L\Lambda_{z}\right)}^{2}+4L\Lambda_{z}\text{sinh}{\left(\frac{L\Lambda_{z}}{2}\right)}^{2}+4\text{sinh}\left(L\Lambda_{z}\right)\text{sinh}{\left(\frac{L\Lambda_{z}}{2}\right)}^{2} \end{array}$$

(30) $$\begin{array}{ccc} \eta_{y} & = & {\left(L^{2}{\Lambda_{y}}^{2}\Omega_{y}-1\right)}^{2}(4L^{5}{\Lambda_{y}}^{5}{\Omega_{y}}^{2}\text{sinh}{\left(\frac{L\Lambda_{y}}{2}\right)}^{2}-4L^{4}{\Lambda_{y}}^{4}{\Omega_{y}}^{2}\text{sinh}\left(L\Lambda_{y}\right)\text{sinh}{\left(\frac{L\Lambda_{y}}{2}\right)}^{2}\\ & & +2L^{4}{\Lambda_{y}}^{4}\Omega_{y}\text{sinh}\left(L\Lambda_{y}\right)-2L^{3}{\Lambda_{y}}^{3}\Omega_{y}\text{sinh}{\left(L\Lambda_{y}\right)}^{2}\\ & & -8L^{3}{\Lambda_{y}}^{3}\Omega_{y}\text{sinh}{\left(\frac{L\Lambda_{y}}{2}\right)}^{2}+2L^{3}{\Lambda_{y}}^{3}\text{sinh}{\left(\frac{L\Lambda_{y}}{2}\right)}^{2}+L^{3}{\Lambda_{y}}^{3}+8L^{2}{\Lambda_{y}}^{2}\Omega_{y}\text{sinh}\left(L\Lambda_{y}\right)\text{sinh}{\left(\frac{L\Lambda_{y}}{2}\right)}^{2}\\ & & -3L^{2}{\Lambda_{y}}^{2}\text{sinh}\left(L\Lambda_{y}\right)+12L\Lambda_{y}\text{sinh}{\left(\frac{L\Lambda_{y}}{2}\right)}^{2}\\ & & -4\text{sinh}\left(L\Lambda_{y}\right)\text{sinh}{\left(\frac{L\Lambda_{y}}{2}\right)}^{2}) \end{array}$$

(31) $$\begin{array}{ccc} \eta_{z} & = & {\left(L^{2}{\Lambda_{z}}^{2}\Omega_{z}-1\right)}^{2}(4L^{5}{\Lambda_{z}}^{5}{\Omega_{z}}^{2}\text{sinh}{\left(\frac{L\Lambda_{z}}{2}\right)}^{2}-4L^{4}{\Lambda_{z}}^{4}{\Omega_{z}}^{2}\text{sinh}\left(L\Lambda_{z}\right)\text{sinh}{\left(\frac{L\Lambda_{z}}{2}\right)}^{2}\\ & & +\;2L^{4}{\Lambda_{z}}^{4}\Omega_{z}\text{sinh}\left(L\Lambda_{z}\right)-2L^{3}{\Lambda_{z}}^{3}\Omega_{z}\text{sinh}{\left(L\Lambda_{z}\right)}^{2}\\ & & -8L^{3}{\Lambda_{z}}^{3}\Omega_{z}\text{sinh}{\left(\frac{L\Lambda_{z}}{2}\right)}^{2}+2L^{3}{\Lambda_{z}}^{3}\text{sinh}{\left(\frac{L\Lambda_{z}}{2}\right)}^{2}+L^{3}{\Lambda_{z}}^{3}\\ & & +\;8L^{2}{\Lambda_{z}}^{2}\Omega_{z}\text{sinh}\left(L\Lambda_{z}\right)\text{sinh}{\left(\frac{L\Lambda_{z}}{2}\right)}^{2}-3L^{2}{\Lambda_{z}}^{2}\text{sinh}\left(L\Lambda_{z}\right)+12L\Lambda_{z}\text{sinh}{\left(\frac{L\Lambda_{z}}{2}\right)}^{2}\\ & & -4\text{sinh}\left(L\Lambda_{z}\right)\text{sinh}{\left(\frac{L\Lambda_{z}}{2}\right)}^{2}) \end{array}$$

(32) $$\begin{array}{ccc} \omega_{y} & = & 2(4L^{5}{\Lambda_{y}}^{5}{\Omega_{y}}^{2}\text{sinh}{\left(\frac{L\Lambda_{y}}{2}\right)}^{2}+4L^{4}{\Lambda_{y}}^{4}{\Omega_{y}}^{2}\text{sinh}\left(L\Lambda_{y}\right)\text{sinh}{\left(\frac{L\Lambda_{y}}{2}\right)}^{2}\\ & & +2L^{4}{\Lambda_{y}}^{4}\Omega_{y}\text{sinh}\left(L\Lambda_{y}\right)+2L^{3}{\Lambda_{y}}^{3}\Omega_{y}\text{sinh}{\left(L\Lambda_{y}\right)}^{2}\\ & & -8L^{3}{\Lambda_{y}}^{3}\Omega_{y}\text{sinh}{\left(\frac{L\Lambda_{y}}{2}\right)}^{2}+L^{3}{\Lambda_{y}}^{3}-8L^{2}{\Lambda_{y}}^{2}\Omega_{y}\text{sinh}\left(L\Lambda_{y}\right)\text{sinh}{\left(\frac{L\Lambda_{y}}{2}\right)}^{2}\\ & & +2L^{2}{\Lambda_{y}}^{2}\text{sinh}\left(L\Lambda_{y}\right)\text{sinh}{\left(\frac{L\Lambda_{y}}{2}\right)}^{2}-L^{2}{\Lambda_{y}}^{2}\text{sinh}\left(L\Lambda_{y}\right)\\ & & -2L\Lambda_{y}\text{sinh}{\left(L\Lambda_{y}\right)}^{2}+4L\Lambda_{y}\text{sinh}{\left(\frac{L\Lambda_{y}}{2}\right)}^{2}+4\text{sinh}\left(L\Lambda_{y}\right)\text{sinh}{\left(\frac{L\Lambda_{y}}{2}\right)}^{2}) \end{array}$$

(33) $$\begin{array}{ccc} \omega_{z} & = & 2(4L^{5}{\Lambda_{z}}^{5}{\Omega_{z}}^{2}\text{sinh}{\left(\frac{L\Lambda_{z}}{2}\right)}^{2}+4L^{4}{\Lambda_{z}}^{4}{\Omega_{z}}^{2}\text{sinh}\left(L\Lambda_{z}\right)\text{sinh}{\left(\frac{L\Lambda_{z}}{2}\right)}^{2}\\ & & +2L^{4}{\Lambda_{z}}^{4}\Omega_{z}\text{sinh}\left(L\Lambda_{z}\right)+2L^{3}{\Lambda_{z}}^{3}\Omega_{z}\text{sinh}{\left(L\Lambda_{z}\right)}^{2}\\ & & -8L^{3}{\Lambda_{z}}^{3}\Omega_{z}\text{sinh}{\left(\frac{L\Lambda_{z}}{2}\right)}^{2}+L^{3}{\Lambda_{z}}^{3}-8L^{2}{\Lambda_{z}}^{2}\Omega_{z}\text{sinh}\left(L\Lambda_{z}\right)\text{sinh}{\left(\frac{L\Lambda_{z}}{2}\right)}^{2}\\ & & +\;2L^{2}{\Lambda_{z}}^{2}\text{sinh}\left(L\Lambda_{z}\right)\text{sinh}{\left(\frac{L\Lambda_{z}}{2}\right)}^{2}-L^{2}{\Lambda_{z}}^{2}\text{sinh}\left(L\Lambda_{z}\right)\\ & & -2L\Lambda_{z}\text{sinh}{\left(L\Lambda_{z}\right)}^{2}+4L\Lambda_{z}\text{sinh}{\left(\frac{L\Lambda_{z}}{2}\right)}^{2}+4\text{sinh}\left(L\Lambda_{z}\right)\text{sinh}{\left(\frac{L\Lambda_{z}}{2}\right)}^{2}) \end{array}$$

(34) $$\begin{array}{ccc} \psi_{y} & = & -4L^{5}{\Lambda_{y}}^{5}{\Omega_{y}}^{2}\text{sinh}{\left(\frac{L\Lambda_{y}}{2}\right)}^{2}-4L^{4}{\Lambda_{y}}^{4}{\Omega_{y}}^{2}\text{sinh}\left(L\Lambda_{y}\right)\text{sinh}{\left(\frac{L\Lambda_{y}}{2}\right)}^{2}\\ & & -2L^{4}{\Lambda_{y}}^{4}\Omega_{y}\text{sinh}\left(L\Lambda_{y}\right)-2L^{3}{\Lambda_{y}}^{3}\Omega_{y}\text{sinh}{\left(L\Lambda_{y}\right)}^{2}\\ & & +8L^{3}{\Lambda_{y}}^{3}\Omega_{y}\text{sinh}{\left(\frac{L\Lambda_{y}}{2}\right)}^{2}-2L^{3}{\Lambda_{y}}^{3}\text{sinh}{\left(\frac{L\Lambda_{y}}{2}\right)}^{2}-L^{3}{\Lambda_{y}}^{3}\\ & & +8L^{2}{\Lambda_{y}}^{2}\Omega_{y}\text{sinh}\left(L\Lambda_{y}\right)\text{sinh}{\left(\frac{L\Lambda_{y}}{2}\right)}^{2}+L^{2}{\Lambda_{y}}^{2}\text{sinh}\left(L\Lambda_{y}\right)\\ & & +2L\Lambda_{y}\text{sinh}{\left(L\Lambda_{y}\right)}^{2}-4L\Lambda_{y}\text{sinh}{\left(\frac{L\Lambda_{y}}{2}\right)}^{2}-4\text{sinh}\left(L\Lambda_{y}\right)\text{sinh}{\left(\frac{L\Lambda_{y}}{2}\right)}^{2} \end{array}$$

(35) $$\begin{array}{ccc} \psi_{z} & = & -4L^{5}{\Lambda_{z}}^{5}{\Omega_{z}}^{2}\text{sinh}{\left(\frac{L\Lambda_{z}}{2}\right)}^{2}-4L^{4}{\Lambda_{z}}^{4}{\Omega_{z}}^{2}\text{sinh}\left(L\Lambda_{z}\right)\text{sinh}{\left(\frac{L\Lambda_{z}}{2}\right)}^{2}\\ & & -\;2L^{4}{\Lambda_{z}}^{4}\Omega_{z}\text{sinh}\left(L\Lambda_{z}\right)-2L^{3}{\Lambda_{z}}^{3}\Omega_{z}\text{sinh}{\left(L\Lambda_{z}\right)}^{2}\\ & & +8L^{3}{\Lambda_{z}}^{3}\Omega_{z}\text{sinh}{\left(\frac{L\Lambda_{z}}{2}\right)}^{2}-2L^{3}{\Lambda_{z}}^{3}\text{sinh}{\left(\frac{L\Lambda_{z}}{2}\right)}^{2}-L^{3}{\Lambda_{z}}^{3}\\ & & +8L^{2}{\Lambda_{z}}^{2}\Omega_{z}\text{sinh}\left(L\Lambda_{z}\right)\text{sinh}{\left(\frac{L\Lambda_{z}}{2}\right)}^{2}+L^{2}{\Lambda_{z}}^{2}\text{sinh}\left(L\Lambda_{z}\right)\\ & & +2L\Lambda_{z}\text{sinh}{\left(L\Lambda_{z}\right)}^{2}-4L\Lambda_{z}\text{sinh}{\left(\frac{L\Lambda_{z}}{2}\right)}^{2}-4\text{sinh}\left(L\Lambda_{z}\right)\text{sinh}{\left(\frac{L\Lambda_{z}}{2}\right)}^{2} \end{array}$$

For a compressive force (*P* < 0), the geometric component of the tangent stiffness matrix can be written in terms of trigonometric functions, and the coefficients become:

(36) $$\begin{array}{ccc} D_{y}\; & = & \left(4\sin{\left(\frac{L\Lambda_{y}}{2}\right)}^{2}-L\Lambda_{y}\sin\left(L\Lambda_{y}\right)+4L^{2}{\Lambda_{y}}^{2}\Omega_{y}\sin{\left(\frac{L\Lambda_{y}}{2}\right)}^{2}\right);\\ C_{y}\; & = & 2\sin{\left(\frac{L\Lambda_{y}}{2}\right)}^{2}\left(L\Lambda_{y}-\sin\left(L\Lambda_{y}\right)\right) \end{array}$$

(37) $$\begin{array}{ccc} \alpha_{y}\; & = & \Lambda_{y}(3L\Lambda_{y}-3\sin\left(L\Lambda_{y}\right)+2L^{3}{\Lambda_{y}}^{3}\Omega_{y}+L^{5}{\Lambda_{y}}^{5}{\Omega_{y}}^{2}-2L\Lambda_{y}\sin{\left(\frac{L\Lambda_{y}}{2}\right)}^{2}-2L^{2}{\Lambda_{y}}^{2}\Omega_{y}\sin\left(L\Lambda_{y}\right)\\ & & +L^{4}{\Lambda_{y}}^{4}{\Omega_{y}}^{2}\sin\left(L\Lambda_{y}\right)); \end{array}$$

(38) $$\begin{array}{ccc} \beta_{y} & = & 2(L^{2}{\Lambda_{y}}^{2}+4\sin{\left(\frac{L\Lambda_{y}}{2}\right)}^{2}-2L\Lambda_{y}\sin\left(L\Lambda_{y}\right)-L^{2}{\Lambda_{y}}^{2}\sin{\left(\frac{L\Lambda_{y}}{2}\right)}^{2}\\ & & +4L^{4}{\Lambda_{y}}^{4}{\Omega_{y}}^{2}\sin{\left(\frac{L\Lambda_{y}}{2}\right)}^{2}-2L^{3}{\Lambda_{y}}^{3}\Omega_{y}\sin\left(L\Lambda_{y}\right)+8L^{2}{\Lambda_{y}}^{2}\Omega_{y}\sin{\left(\frac{L\Lambda_{y}}{2}\right)}^{2}) \end{array}$$

(39) $$\begin{array}{ccc} \phi_{y} & = & 4L^{4}{\Lambda_{y}}^{4}{\Omega_{y}}^{2}\sin{\left(L\Lambda_{y}\right)}^{2}-16L^{4}{\Lambda_{y}}^{4}{\Omega_{y}}^{2}\sin{\left(\frac{L\Lambda_{y}}{2}\right)}^{2}+8L^{3}{\Lambda_{y}}^{3}\Omega_{y}\sin\left(L\Lambda_{y}\right)\sin{\left(\frac{L\Lambda_{y}}{2}\right)}^{2}\\ & & +8L^{2}{\Lambda_{y}}^{2}\Omega_{y}\sin{\left(L\Lambda_{y}\right)}^{2}-32L^{2}{\Lambda_{y}}^{2}\Omega_{y}\sin{\left(\frac{L\Lambda_{y}}{2}\right)}^{2}\\ & & -L^{2}{\Lambda_{y}}^{2}\sin{\left(L\Lambda_{y}\right)}^{2}+8L\Lambda_{y}\sin\left(L\Lambda_{y}\right)\sin{\left(\frac{L\Lambda_{y}}{2}\right)}^{2}\\ & & +\;4\sin{\left(L\Lambda_{y}\right)}^{2}-16\sin{\left(\frac{L\Lambda_{y}}{2}\right)}^{2} \end{array}$$

(40) $$\begin{array}{ccc} \varphi_{y} & = & -{\left(\Omega_{y}L^{2}{\Lambda_{y}}^{2}+1\right)}^{2}(\sin\left(2L\Lambda_{y}\right)+L^{3}{\Lambda_{y}}^{3}+5L^{3}{\Lambda_{y}}^{3}\Omega_{y}-\frac{L^{2}{\Lambda_{y}}^{2}\sin\left(2L\Lambda_{y}\right)}{2}\\ & & +\;2L^{5}{\Lambda_{y}}^{5}{\Omega_{y}}^{2}+2L\Lambda_{y}\left(2\sin{\left(L\Lambda_{y}\right)}^{2}-1\right)+2L^{2}{\Lambda_{y}}^{2}\Omega_{y}\sin\left(2L\Lambda_{y}\right)\\ & & +L^{3}{\Lambda_{y}}^{3}\Omega_{y}\left(2\sin{\left(L\Lambda_{y}\right)}^{2}-1\right)+L^{4}{\Lambda_{y}}^{4}{\Omega_{y}}^{2}\sin\left(2L\Lambda_{y}\right)\\ & & +\left(2\sin{\left(\frac{L\Lambda_{y}}{2}\right)}^{2}-1\right)\left(2L^{5}{\Lambda_{y}}^{5}{\Omega_{y}}^{2}+4L^{3}{\Lambda_{y}}^{3}\Omega_{y}-2L\Lambda_{y})\right)\\ & & +\sin\left(L\Lambda_{y}\right){\left(\Omega_{y}L^{2}{\Lambda_{y}}^{2}+1\right)}^{2}\left(2L^{4}{\Lambda_{y}}^{4}{\Omega_{y}}^{2}+2L^{4}{\Lambda_{y}}^{4}\Omega_{y}+4L^{2}{\Lambda_{y}}^{2}\Omega_{y}+2L^{2}{\Lambda_{y}}^{2}+2\right) \end{array}$$

(41) $$\begin{array}{ccc} \eta_{y} & = & \;{\left(\Omega_{y}\;L^{2}\;{\Lambda_{y}}^{2}+1\right)}^{2}\;(\sin\left(2\;L\;\Lambda_{y}\right)+6\;L\;\Lambda_{y}+5\;L^{3}\;{\Lambda_{y}}^{3}\;\Omega_{y}+2\;L^{5}\;{\Lambda_{y}}^{5}\;{\Omega_{y}}^{2}+2\;L^{2}\;{\Lambda_{y}}^{2}\;\Omega_{y}\;\sin\left(2\;L\;\Lambda_{y}\right)\\ & & +L^{3}\;{\Lambda_{y}}^{3}\;\Omega_{y}\;\left(2\;\sin{\left(L\;\Lambda_{y}\right)}^{2}-1\right)+L^{4}\;{\Lambda_{y}}^{4}\;{\Omega_{y}}^{2}\;\sin\left(2\;L\;\Lambda_{y}\right)\\ & & +\;\;\left(2\;\sin{\left(\frac{L\;\Lambda_{y}}{2}\right)}^{2}-1\right)\;\left(2\;L^{5}\;{\Lambda_{y}}^{5}\;{\Omega_{y}}^{2}+4\;L^{3}\;{\Lambda_{y}}^{3}\;\Omega_{y}-L^{3}\;{\Lambda_{y}}^{3}+6\;L\;\Lambda_{y}\right))\\ & & -\;\sin\left(L\;\Lambda_{y}\right)\;{\left(\Omega_{y}\;L^{2}\;{\Lambda_{y}}^{2}+1\right)}^{2}\;\left(2\;L^{4}\;{\Lambda_{y}}^{4}\;{\Omega_{y}}^{2}+2\;L^{4}\;{\Lambda_{y}}^{4}\;\Omega_{y}+4\;L^{2}\;{\Lambda_{y}}^{2}\;\Omega_{y}+3\;L^{2}\;{\Lambda_{y}}^{2}+2\right) \end{array}$$

(42) $$\begin{array}{ccc} \omega_{y} & = & \sin\left(L\Lambda_{y}\right)(4\cos\left(L\Lambda_{y}\right)+4L^{2}{\Lambda_{y}}^{2}-8L^{2}{\Lambda_{y}}^{2}\Omega_{y}+4L^{4}{\Lambda_{y}}^{4}\Omega_{y}-2L^{2}{\Lambda_{y}}^{2}\cos\left(L\Lambda_{y}\right)\\ & & -4L^{4}{\Lambda_{y}}^{4}{\Omega_{y}}^{2}+8L^{2}{\Lambda_{y}}^{2}\Omega_{y}\cos\left(L\Lambda_{y}\right)+4L^{4}{\Lambda_{y}}^{4}{\Omega_{y}}^{2}\cos\left(L\Lambda_{y}\right)-4)\\ & & +4L^{5}{\Lambda_{y}}^{5}{\Omega_{y}}^{2}\cos\left(L\Lambda_{y}\right)-4L^{5}{\Lambda_{y}}^{5}{\Omega_{y}}^{2}-4L^{3}{\Lambda_{y}}^{3}\Omega_{y}\cos{\left(L\Lambda_{y}\right)}^{2}+8L^{3}{\Lambda_{y}}^{3}\Omega_{y}\cos\left(L\Lambda_{y}\right)\\ & & -\;4L^{3}{\Lambda_{y}}^{3}\Omega_{y}-2L^{3}{\Lambda_{y}}^{3}-4L\Lambda_{y}\cos{\left(L\Lambda_{y}\right)}^{2}+4L\Lambda_{y}\cos\left(L\Lambda_{y}\right) \end{array}$$

(43) $$\begin{array}{ccc} \psi_{y} & = & -\sin\left(L\Lambda_{y}\right)(2\cos\left(L\Lambda_{y}\right)+L^{2}{\Lambda_{y}}^{2}-4L^{2}{\Lambda_{y}}^{2}\Omega_{y}+2L^{4}{\Lambda_{y}}^{4}\Omega_{y}-2L^{4}{\Lambda_{y}}^{4}{\Omega_{y}}^{2}\\ & & +4L^{2}{\Lambda_{y}}^{2}\Omega_{y}\cos\left(L\Lambda_{y}\right)+2L^{4}{\Lambda_{y}}^{4}{\Omega_{y}}^{2}\cos\left(L\Lambda_{y}\right)-2)-2L^{5}{\Lambda_{y}}^{5}{\Omega_{y}}^{2}\cos\left(L\Lambda_{y}\right)\\ & & +2L^{5}{\Lambda_{y}}^{5}{\Omega_{y}}^{2}+2L^{3}{\Lambda_{y}}^{3}\Omega_{y}\cos{\left(L\Lambda_{y}\right)}^{2}-4L^{3}{\Lambda_{y}}^{3}\Omega_{y}\cos\left(L\Lambda_{y}\right)+2L^{3}{\Lambda_{y}}^{3}\Omega_{y}+L^{3}{\Lambda_{y}}^{3}\cos\left(L\Lambda_{y}\right)\\ & & +2L\Lambda_{y}\cos{\left(L\Lambda_{y}\right)}^{2}-2L\Lambda_{y}\cos\left(L\Lambda_{y}\right) \end{array}$$

(44) $$\begin{array}{ccc} \gamma_{y}\; & = & {\left(\Omega_{y}L^{2}{\Lambda_{y}}^{2}+1\right)}^{2}\left(L^{2}{\Lambda_{y}}^{2}-8\sin{\left(\frac{L\Lambda_{y}}{2}\right)}^{2}+L\Lambda_{y}\sin\left(L\Lambda_{y}\right)\right);\\ \gamma_{z}\; & = & {\left(\Omega_{z}L^{2}{\Lambda_{z}}^{2}+1\right)}^{2}\left(L^{2}{\Lambda_{z}}^{2}-8\sin{\left(\frac{L\Lambda_{z}}{2}\right)}^{2}+L\Lambda_{z}\sin\left(L\Lambda_{z}\right)\right) \end{array}$$

(45) $$\begin{array}{ccc} D_{z}\; & = & \left(4\sin{\left(\frac{L\Lambda_{z}}{2}\right)}^{2}-L\Lambda_{z}\sin\left(L\Lambda_{z}\right)+4L^{2}{\Lambda_{z}}^{2}\Omega_{z}\sin{\left(\frac{L\Lambda_{z}}{2}\right)}^{2}\right);\\ \;C_{z}\; & = & 2\sin{\left(\frac{L\Lambda_{z}}{2}\right)}^{2}\left(L\Lambda_{z}-\sin\left(L\Lambda_{z}\right)\right) \end{array}$$

(46) $$\begin{array}{ccc} \alpha_{z}\; & = & \Lambda_{z}(3L\Lambda_{z}-3\sin\left(L\Lambda_{z}\right)+2L^{3}{\Lambda_{z}}^{3}\Omega_{z}+L^{5}{\Lambda_{z}}^{5}{\Omega_{z}}^{2}-2L\Lambda_{z}\sin{\left(\frac{L\Lambda_{z}}{2}\right)}^{2}\\ & & -2L^{2}{\Lambda_{z}}^{2}\Omega_{z}\sin\left(L\Lambda_{z}\right)+L^{4}{\Lambda_{z}}^{4}{\Omega_{z}}^{2}\sin\left(L\Lambda_{z}\right)) \end{array}$$

(47) $$\begin{array}{ccc} \beta_{z} & = & 2(L^{2}{\Lambda_{z}}^{2}+4\sin{\left(\frac{L\Lambda_{z}}{2}\right)}^{2}-2L\Lambda_{z}\sin\left(L\Lambda_{z}\right)-L^{2}{\Lambda_{z}}^{2}\sin{\left(\frac{L\Lambda_{z}}{2}\right)}^{2}\\ & & +4L^{4}{\Lambda_{z}}^{4}{\Omega_{z}}^{2}\sin{\left(\frac{L\Lambda_{z}}{2}\right)}^{2}-2L^{3}{\Lambda_{z}}^{3}\Omega_{z}\sin\left(L\Lambda_{z}\right)+8L^{2}{\Lambda_{z}}^{2}\Omega_{z}\sin{\left(\frac{L\Lambda_{z}}{2}\right)}^{2}) \end{array}$$

(48) $$\begin{array}{ccc} \phi_{z} & = & 4L^{4}{\Lambda_{z}}^{4}{\Omega_{z}}^{2}\sin{\left(L\Lambda_{z}\right)}^{2}-16L^{4}{\Lambda_{z}}^{4}{\Omega_{z}}^{2}\sin{\left(\frac{L\Lambda_{z}}{2}\right)}^{2}+8L^{3}{\Lambda_{z}}^{3}\Omega_{z}\sin\left(L\Lambda_{z}\right)\sin{\left(\frac{L\Lambda_{z}}{2}\right)}^{2}\\ & & +\;8L^{2}{\Lambda_{z}}^{2}\Omega_{z}\sin{\left(L\Lambda_{z}\right)}^{2}-32L^{2}{\Lambda_{z}}^{2}\Omega_{z}\sin{\left(\frac{L\Lambda_{z}}{2}\right)}^{2}\\ & & -L^{2}{\Lambda_{z}}^{2}\sin{\left(L\Lambda_{z}\right)}^{2}+8L\Lambda_{z}\sin\left(L\Lambda_{z}\right)\sin{\left(\frac{L\Lambda_{z}}{2}\right)}^{2}+4\sin{\left(L\Lambda_{z}\right)}^{2}-16\sin{\left(\frac{L\Lambda_{z}}{2}\right)}^{2} \end{array}$$

(49) $$\begin{array}{ccc} \varphi_{z} & = & -{\left(\Omega_{z}L^{2}{\Lambda_{z}}^{2}+1\right)}^{2}(\sin\left(2L\Lambda_{z}\right)+L^{3}{\Lambda_{z}}^{3}+5L^{3}{\Lambda_{z}}^{3}\Omega_{z}-\frac{L^{2}{\Lambda_{z}}^{2}\sin\left(2L\Lambda_{z}\right)}{2}\\ & & +2L^{5}{\Lambda_{z}}^{5}{\Omega_{z}}^{2}+2L\Lambda_{z}\left(2\sin{\left(L\Lambda_{z}\right)}^{2}-1\right)+2L^{2}{\Lambda_{z}}^{2}\Omega_{z}\sin\left(2L\Lambda_{z}\right)\\ & & +L^{3}{\Lambda_{z}}^{3}\Omega_{z}\left(2\sin{\left(L\Lambda_{z}\right)}^{2}-1\right)+L^{4}{\Lambda_{z}}^{4}{\Omega_{z}}^{2}\sin\left(2L\Lambda_{z}\right)\\ & & +\;\left(2\sin{\left(\frac{L\Lambda_{z}}{2}\right)}^{2}-1\right)\left(2L^{5}{\Lambda_{z}}^{5}{\Omega_{z}}^{2}+4L^{3}{\Lambda_{z}}^{3}\Omega_{z}-2L\Lambda_{z}\right))\\ & & +\sin\left(L\Lambda_{z}\right){\left(\Omega_{z}L^{2}{\Lambda_{z}}^{2}+1\right)}^{2}\left(2L^{4}{\Lambda_{z}}^{4}{\Omega_{z}}^{2}+2L^{4}{\Lambda_{z}}^{4}\Omega_{z}+4L^{2}{\Lambda_{z}}^{2}\Omega_{z}+2L^{2}{\Lambda_{z}}^{2}+2\right) \end{array}$$

(50) $$\begin{array}{ccc} \eta_{z} & = & {\left(\Omega_{z}\;L^{2}\;{\Lambda_{z}}^{2}+1\right)}^{2}\;(\sin\left(2\;L\;\Lambda_{z}\right)+6\;L\;\Lambda_{z}+5\;L^{3}\;{\Lambda_{z}}^{3}\;\Omega_{z}\\ & & +2\;L^{5}\;{\Lambda_{z}}^{5}\;{\Omega_{z}}^{2}+2\;L^{2}\;{\Lambda_{z}}^{2}\;\Omega_{z}\;\sin\left(2\;L\;\Lambda_{z}\right)+L^{3}\;{\Lambda_{z}}^{3}\;\Omega_{z}\;\left(2\;\sin{\left(L\;\Lambda_{z}\right)}^{2}-1\right)+L^{4}\;{\Lambda_{z}}^{4}\;{\Omega_{z}}^{2}\;\sin\left(2\;L\;\Lambda_{z}\right)\\ & & +\;\;\left(2\;\sin{\left(\frac{L\;\Lambda_{z}}{2}\right)}^{2}-1\right)\;\left(2\;L^{5}\;{\Lambda_{z}}^{5}\;{\Omega_{z}}^{2}+4\;L^{3}\;{\Lambda_{z}}^{3}\;\Omega_{z}-L^{3}\;{\Lambda_{z}}^{3}+6\;L\;\Lambda_{z}\right))\\ & & -\;\sin\left(L\;\Lambda_{z}\right)\;{\left(\Omega_{z}\;L^{2}\;{\Lambda_{z}}^{2}+1\right)}^{2}\;\left(2\;L^{4}\;{\Lambda_{z}}^{4}\;{\Omega_{z}}^{2}+2\;L^{4}\;{\Lambda_{z}}^{4}\;\Omega_{z}+4\;L^{2}\;{\Lambda_{z}}^{2}\;\Omega_{z}+3\;L^{2}\;{\Lambda_{z}}^{2}+2\right) \end{array}$$

(51) $$\begin{array}{ccc} \omega_{z} & = & \sin\left(L\Lambda_{z}\right)(4\cos\left(L\Lambda_{z}\right)+4L^{2}{\Lambda_{z}}^{2}-8L^{2}{\Lambda_{z}}^{2}\Omega_{z}+4L^{4}{\Lambda_{z}}^{4}\Omega_{z}-2L^{2}{\Lambda_{z}}^{2}\cos\left(L\Lambda_{z}\right)\\ & & -4L^{4}{\Lambda_{z}}^{4}{\Omega_{z}}^{2}+8L^{2}{\Lambda_{z}}^{2}\Omega_{z}\cos\left(L\Lambda_{z}\right)+4L^{4}{\Lambda_{z}}^{4}{\Omega_{z}}^{2}\cos\left(L\Lambda_{z}\right)-4)\\ & & \;+4L^{5}{\Lambda_{z}}^{5}{\Omega_{z}}^{2}\cos\left(L\Lambda_{z}\right)-4L^{5}{\Lambda_{z}}^{5}{\Omega_{z}}^{2}-4L^{3}{\Lambda_{z}}^{3}\Omega_{z}\cos{\left(L\Lambda_{z}\right)}^{2}+8L^{3}{\Lambda_{z}}^{3}\Omega_{z}\cos\left(L\Lambda_{z}\right)\\ & & -4L^{3}{\Lambda_{z}}^{3}\Omega_{z}-2L^{3}{\Lambda_{z}}^{3}-4L\Lambda_{z}\cos{\left(L\Lambda_{z}\right)}^{2}+4L\Lambda_{z}\cos\left(L\Lambda_{z}\right) \end{array}$$

(52) $$\begin{array}{ccc} \psi_{z} & = & -\sin\left(L\Lambda_{z}\right)(2\cos\left(L\Lambda_{z}\right)+L^{2}{\Lambda_{z}}^{2}-4L^{2}{\Lambda_{z}}^{2}\Omega_{z}+2L^{4}{\Lambda_{z}}^{4}\Omega_{z}-2L^{4}{\Lambda_{z}}^{4}{\Omega_{z}}^{2}+4L^{2}{\Lambda_{z}}^{2}\Omega_{z}\cos\left(L\Lambda_{z}\right)\\ & & +2L^{4}{\Lambda_{z}}^{4}{\Omega_{z}}^{2}\cos\left(L\Lambda_{z}\right)-2)-2L^{5}{\Lambda_{z}}^{5}{\Omega_{z}}^{2}\cos\left(L\Lambda_{z}\right)\\ & & +2L^{5}{\Lambda_{z}}^{5}{\Omega_{z}}^{2}+2L^{3}{\Lambda_{z}}^{3}\Omega_{z}\cos{\left(L\Lambda_{z}\right)}^{2}-4L^{3}{\Lambda_{z}}^{3}\Omega_{z}\cos\left(L\Lambda_{z}\right)+2L^{3}{\Lambda_{z}}^{3}\Omega_{z}\\ & & +\;L^{3}{\Lambda_{z}}^{3}\cos\left(L\Lambda_{z}\right)+2L\Lambda_{z}\cos{\left(L\Lambda_{z}\right)}^{2}-2L\Lambda_{z}\cos\left(L\Lambda_{z}\right) \end{array}$$

Interaction matrix of axial load and torsion - $\left[K_{int}\right]$:

For a tensile force (*P* > 0), the matrix that considers the interaction between the axial load and the torsion is given according:

The coefficients are written in terms of hyperbolic functions:

(53) $$\begin{array}{ccc} \varphi_{y} & = & \left(1-\Omega_{y}{\Lambda_{y}}^{2}L^{2}\right)\left[\Lambda_{y}L-2sinh\left(\Lambda_{y}L\right)-2\cosh\left(\Lambda_{y}L\right)+\Lambda_{y}L\left(\cosh\left(\Lambda_{y}L\right)+sinh\left(\Lambda_{y}L\right)\right)+2\right];\\ \phi_{y} & = & \left(\cosh\left(\Lambda_{y}L\right)+sinh\left(\Lambda_{y}L\right)-1\right) \end{array}$$

(54) $$\begin{array}{ccc} \varphi_{z} & = & \left(1-\Omega_{z}{\Lambda_{z}}^{2}L^{2}\right)\left[\Lambda_{z}L-2sinh\left(\Lambda_{z}L\right)-2\cosh\left(\Lambda_{z}L\right)+\Lambda_{z}L\left(\cosh\left(\Lambda_{z}L\right)+sinh\left(\Lambda_{z}L\right)\right)+2\right];\\ \phi_{z} & = & \left(\cosh\left(\Lambda_{z}L\right)+sinh\left(\Lambda_{z}L\right)-1\right) \end{array}$$

(55) $$\begin{array}{ccc} D_{y} & = & \sinh\left(\frac{\Lambda_{z}L}{2}\right)(2\sinh\left(\frac{\Lambda_{y}L}{2}\right)-\Lambda_{y}\mathrm{Lcosh}\left(\frac{\Lambda_{y}L}{2}\right)\\ & & -2\Omega_{y}{\Lambda_{y}}^{2}L^{2}\sinh\left(\frac{\Lambda_{y}L}{2}\right));\\ D_{z} & = & \sinh\left(\frac{\Lambda_{y}L}{2}\right)(2\sinh\left(\frac{\Lambda_{z}L}{2}\right)-\Lambda_{z}\mathrm{Lcosh}\left(\frac{\Lambda_{z}L}{2}\right)\\ & & -2\Omega_{z}{\Lambda_{z}}^{2}L^{2}\sinh\left(\frac{\Lambda_{z}L}{2}\right)) \end{array}$$

(56) $$\psi =\Lambda_{y}cosh\left(\frac{\Lambda_{y}L}{2}\right)sinh\left(\frac{\Lambda_{z}L}{2}\right)-\Lambda_{z}cosh\left(\frac{\Lambda_{z}L}{2}\right)sinh\left(\frac{\Lambda_{y}L}{2}\right)$$

(57) $$\begin{array}{ccc} D_{1} & = & \;\left(L\;\Lambda_{y}\;\text{sinh}\left(L\;\Lambda_{y}\right)-4\;\text{sinh}{\left(\frac{L\;\Lambda_{y}}{2}\right)}^{2}+4\;L^{2}\;{\Lambda_{y}}^{2}\;\Omega_{y}\;\text{sinh}{\left(\frac{L\;\Lambda_{y}}{2}\right)}^{2}\right)\\ & & \times \;\;\left(L\;\Lambda_{z}\;\text{sinh}\left(L\;\Lambda_{z}\right)-4\;\text{sinh}{\left(\frac{L\;\Lambda_{z}}{2}\right)}^{2}+4\;L^{2}\;{\Lambda_{z}}^{2}\;\Omega_{z}\;\text{sinh}{\left(\frac{L\;\Lambda_{z}}{2}\right)}^{2}\right) \end{array}$$

(58) $$\begin{array}{ccc} D_{2} & = & \left({\Lambda_{y}}^{2}-{\Lambda_{z}}^{2}\right)\;\left(L\;\Lambda_{y}\;\text{sinh}\left(L\;\Lambda_{y}\right)-2\;L^{2}\;{\Lambda_{y}}^{2}\;\Omega_{y}-2\;\text{cosh}\left(L\;\Lambda_{y}\right)+2\;L^{2}\;{\Lambda_{y}}^{2}\;\Omega_{y}\;\text{cosh}\left(L\;\Lambda_{y}\right)+2\right)\;\\ & & +\left(L\;\Lambda_{z}\;\text{sinh}\left(L\;\Lambda_{z}\right)-2\;L^{2}\;{\Lambda_{z}}^{2}\;\Omega_{z}-2\;\text{cosh}\left(L\;\Lambda_{z}\right)+2\;L^{2}\;{\Lambda_{z}}^{2}\;\Omega_{z}\;\text{cosh}\left(L\;\Lambda_{z}\right)+2\right) \end{array}$$

(59) $$\begin{array}{ccc} \varphi_{1} & = & L^{3}\;\left(2\;{\Lambda_{y}}^{2}\;{\Lambda_{z}}^{3}\;\;\text{sinh}\left(L\;\Lambda_{z}\right)\;\text{sinh}{\left(\frac{L\;\Lambda_{y}}{2}\right)}^{2}\;\left(\Omega_{y}-\Omega_{z}\right)-2\;{\Lambda_{y}}^{3}\;{\Lambda_{z}}^{2}\;\text{sinh}\left(L\;\Lambda_{y}\right)\;\text{sinh}{\left(\frac{L\;\Lambda_{z}}{2}\right)}^{2}\;\left(\Omega_{y}-\Omega_{z}\right)\right)\\ & & +L\left({\Lambda_{y}}^{2}-{\Lambda_{z}}^{2}\right)\;(\Lambda_{y}\;\text{sinh}\left(L\;\Lambda_{y}\right)-\Lambda_{z}\;\text{sinh}\left(L\;\Lambda_{z}\right)-\Lambda_{y}\;\text{sinh}\left(L\;\Lambda_{y}\right)\;\\ & & \times \left(2\;\text{sinh}{\left(\frac{L\;\Lambda_{z}}{2}\right)}^{2}+1\right)+\Lambda_{z}\;\text{sinh}\left(L\;\Lambda_{z}\right)\;\left(2\;\text{sinh}{\left(\frac{L\;\Lambda_{y}}{2}\right)}^{2}+1\right))\\ & & \;-\frac{L^{2}\Lambda_{y}\Lambda_{z}\;\;\left(4\;\Lambda_{y}\;\Lambda_{z}\;\text{sinh}{\left(\frac{L\;\Lambda_{y}}{2}\right)}^{2}+4\;\Lambda_{y}\;\Lambda_{z}\;\text{sinh}{\left(\frac{L\;\Lambda_{z}}{2}\right)}^{2}-{\Lambda_{y}}^{2}\;\text{sinh}\left(L\;\Lambda_{y}\right)\;\text{sinh}\left(L\;\Lambda_{z}\right)-{\Lambda_{z}}^{2}\;\text{sinh}\left(L\;\Lambda_{y}\right)\;\text{sinh}\left(L\;\Lambda_{z}\right)+8\;\Lambda_{y}\;\Lambda_{z}\;\text{sinh}{\left(\frac{L\;\Lambda_{y}}{2}\right)}^{2}\;\text{sinh}{\left(\frac{L\;\Lambda_{z}}{2}\right)}^{2}\right)}{2} \end{array}$$

(60) $$\begin{array}{ccc} \varphi_{2} & = & 4{\Lambda_{y}}^{2}-4{\Lambda_{z}}^{2}-4{\Lambda_{y}}^{2}\text{cosh}{\left(\frac{L\Lambda_{y}}{2}\right)}^{2}-4{\Lambda_{y}}^{2}\text{cosh}{\left(\frac{L\Lambda_{z}}{2}\right)}^{2}+4{\Lambda_{z}}^{2}\text{cosh}{\left(\frac{L\Lambda_{y}}{2}\right)}^{2}\\ & & +4{\Lambda_{z}}^{2}\text{cosh}{\left(\frac{L\Lambda_{z}}{2}\right)}^{2}-4L^{2}{\Lambda_{y}}^{4}\Omega_{y}+4L^{2}{\Lambda_{z}}^{4}\Omega_{z}+4{\Lambda_{y}}^{2}\text{cosh}{\left(\frac{L\Lambda_{y}}{2}\right)}^{2}\text{cosh}{\left(\frac{L\Lambda_{z}}{2}\right)}^{2}\\ & & -4{\Lambda_{z}}^{2}\text{cosh}{\left(\frac{L\Lambda_{y}}{2}\right)}^{2}\text{cosh}{\left(\frac{L\Lambda_{z}}{2}\right)}^{2}-2L\Lambda_{y}{\Lambda_{z}}^{2}\text{sinh}\left(L\Lambda_{y}\right)+2L{\Lambda_{y}}^{2}\Lambda_{z}\text{sinh}\left(L\Lambda_{z}\right)\\ & & +L^{2}{\Lambda_{y}}^{2}{\Lambda_{z}}^{2}\text{cosh}{\left(\frac{L\Lambda_{y}}{2}\right)}^{2}-L^{2}{\Lambda_{y}}^{2}{\Lambda_{z}}^{2}\text{cosh}{\left(\frac{L\Lambda_{z}}{2}\right)}^{2}+4L^{2}{\Lambda_{y}}^{2}{\Lambda_{z}}^{2}\Omega_{y}\\ & & -4L^{2}{\Lambda_{y}}^{2}{\Lambda_{z}}^{2}\Omega_{z}+4L^{2}{\Lambda_{y}}^{4}\Omega_{y}\text{cosh}{\left(\frac{L\Lambda_{y}}{2}\right)}^{2}+4L^{2}{\Lambda_{y}}^{4}\Omega_{y}\text{cosh}{\left(\frac{L\Lambda_{z}}{2}\right)}^{2}\\ & & -4L^{2}{\Lambda_{z}}^{4}\Omega_{z}\text{cosh}{\left(\frac{L\Lambda_{y}}{2}\right)}^{2}-4L^{2}{\Lambda_{z}}^{4}\Omega_{z}\text{cosh}{\left(\frac{L\Lambda_{z}}{2}\right)}^{2}-4L^{4}{\Lambda_{y}}^{2}{\Lambda_{z}}^{4}\Omega_{y}\Omega_{z}+4L^{4}{\Lambda_{y}}^{4}{\Lambda_{z}}^{2}\Omega_{y}\Omega_{z}\\ & & +L^{3}{\Lambda_{y}}^{3}{\Lambda_{z}}^{2}\Omega_{y}\text{sinh}\left(L\Lambda_{y}\right)-L^{3}{\Lambda_{y}}^{2}{\Lambda_{z}}^{3}\Omega_{z}\text{sinh}\left(L\Lambda_{z}\right)-4L^{2}{\Lambda_{y}}^{2}{\Lambda_{z}}^{2}\Omega_{y}\text{cosh}{\left(\frac{L\Lambda_{y}}{2}\right)}^{2}\\ & & -4L^{2}{\Lambda_{y}}^{2}{\Lambda_{z}}^{2}\Omega_{z}\text{cosh}{\left(\frac{L\Lambda_{y}}{2}\right)}^{2}-4L^{2}{\Lambda_{y}}^{2}{\Lambda_{z}}^{2}\Omega_{y}\text{cosh}{\left(\frac{L\Lambda_{z}}{2}\right)}^{2}\\ & & +\;4L^{2}{\Lambda_{y}}^{2}{\Lambda_{z}}^{2}\Omega_{z}\text{cosh}{\left(\frac{L\Lambda_{z}}{2}\right)}^{2}-4L^{2}{\Lambda_{y}}^{4}\Omega_{y}\text{cosh}{\left(\frac{L\Lambda_{y}}{2}\right)}^{2}\text{cosh}{\left(\frac{L\Lambda_{z}}{2}\right)}^{2}\\ & & +4L^{2}{\Lambda_{z}}^{4}\Omega_{z}\text{cosh}{\left(\frac{L\Lambda_{y}}{2}\right)}^{2}\text{cosh}{\left(\frac{L\Lambda_{z}}{2}\right)}^{2}+L^{3}\Lambda_{y}{\Lambda_{z}}^{4}\Omega_{z}\text{sinh}\left(L\Lambda_{y}\right)-L^{3}{\Lambda_{y}}^{4}\Lambda_{z}\Omega_{y}\text{sinh}\left(L\Lambda_{z}\right)\\ & & +4L\Lambda_{y}{\Lambda_{z}}^{2}\text{cosh}\left(\frac{L\Lambda_{y}}{2}\right)\text{cosh}{\left(\frac{L\Lambda_{z}}{2}\right)}^{2}\text{sinh}\left(\frac{L\Lambda_{y}}{2}\right)-4L^{2}{\Lambda_{y}}^{2}{\Lambda_{z}}^{2}\Omega_{y}\text{cosh}{\left(\frac{L\Lambda_{y}}{2}\right)}^{2}\text{cosh}{\left(\frac{L\Lambda_{z}}{2}\right)}^{2}\\ & & -4L{\Lambda_{y}}^{2}\Lambda_{z}\text{cosh}{\left(\frac{L\Lambda_{y}}{2}\right)}^{2}\text{cosh}\left(\frac{L\Lambda_{z}}{2}\right)\text{sinh}\left(\frac{L\Lambda_{z}}{2}\right)\\ & & -4L^{2}{\Lambda_{y}}^{2}{\Lambda_{z}}^{2}\Omega_{z}\text{cosh}{\left(\frac{L\Lambda_{y}}{2}\right)}^{2}\text{cosh}{\left(\frac{L\Lambda_{z}}{2}\right)}^{2}+4L^{4}{\Lambda_{y}}^{2}{\Lambda_{z}}^{4}\Omega_{y}\Omega_{z}\text{cosh}{\left(\frac{L\Lambda_{y}}{2}\right)}^{2}\\ & & -4L^{4}{\Lambda_{y}}^{4}{\Lambda_{z}}^{2}\Omega_{y}\Omega_{z}\text{cosh}{\left(\frac{L\Lambda_{y}}{2}\right)}^{2}+4L^{4}{\Lambda_{y}}^{2}{\Lambda_{z}}^{4}\Omega_{y}\Omega_{z}\text{cosh}{\left(\frac{L\Lambda_{z}}{2}\right)}^{2}\\ & & +\;2L^{3}{\Lambda_{y}}^{2}{\Lambda_{z}}^{3}\Omega_{z}\text{cosh}{\left(\frac{L\Lambda_{y}}{2}\right)}^{2}\text{cosh}\left(\frac{L\Lambda_{z}}{2}\right)\text{sinh}\left(\frac{L\Lambda_{z}}{2}\right)-2L^{3}{\Lambda_{y}}^{3}{\Lambda_{z}}^{2}\Omega_{y}\text{cosh}\left(\frac{L\Lambda_{y}}{2}\right)\\ & & \times \text{cosh}{\left(\frac{L\Lambda_{z}}{2}\right)}^{2}\text{sinh}\left(\frac{L\Lambda_{y}}{2}\right)-4L^{4}{\Lambda_{y}}^{4}{\Lambda_{z}}^{2}\Omega_{y}\Omega_{z}\text{cosh}{\left(\frac{L\Lambda_{z}}{2}\right)}^{2}\\ & & +4L^{4}{\Lambda_{y}}^{2}{\Lambda_{z}}^{4}\Omega_{y}\Omega_{z}\text{cosh}{\left(\frac{L\Lambda_{y}}{2}\right)}^{2}\text{cosh}{\left(\frac{L\Lambda_{z}}{2}\right)}^{2}+4L^{4}{\Lambda_{y}}^{4}{\Lambda_{z}}^{2}\Omega_{y}\Omega_{z}\text{cosh}{\left(\frac{L\Lambda_{y}}{2}\right)}^{2}\\ & & \times \text{cosh}{\left(\frac{L\Lambda_{z}}{2}\right)}^{2}-2L^{3}\Lambda_{y}{\Lambda_{z}}^{4}\Omega_{z}\text{cosh}\left(\frac{L\Lambda_{y}}{2}\right)\text{cosh}{\left(\frac{L\Lambda_{z}}{2}\right)}^{2}\text{sinh}\left(\frac{L\Lambda_{y}}{2}\right)\\ & & +2L^{3}{\Lambda_{y}}^{4}\Lambda_{z}\Omega_{y}\text{cosh}{\left(\frac{L\Lambda_{y}}{2}\right)}^{2}\text{cosh}\left(\frac{L\Lambda_{z}}{2}\right)\text{sinh}\left(\frac{L\Lambda_{z}}{2}\right) \end{array}$$

For a compressive force (*P* < 0), the interaction between the axial load and the torsion is given according:

The coefficients are written in terms of trigonometric functions:

(61) $$\begin{array}{ccc} \varphi_{y}\; & = & 2\left(\Omega_{y}L^{2}{\Lambda_{y}}^{2}+1\right)(L^{2}{\Lambda_{y}}^{2}+4\sin{\left(\frac{L\Lambda_{y}}{2}\right)}^{2}-2L\Lambda_{y}\sin\left(L\Lambda_{y}\right)-L^{2}{\Lambda_{y}}^{2}\sin{\left(\frac{L\Lambda_{y}}{2}\right)}^{2}\\ & & -L^{3}{\Lambda_{y}}^{3}\Omega_{y}\sin\left(L\Lambda_{y}\right)+4L^{2}{\Lambda_{y}}^{2}\Omega_{y}\sin{\left(\frac{L\Lambda_{y}}{2}\right)}^{2}) \end{array}$$

(62) $$\begin{array}{ccc} \phi_{y}\; & = & (4\sin{\left(\frac{L\Lambda_{y}}{2}\right)}^{2}+2L^{2}{\Lambda_{y}}^{2}\Omega_{y}-L\Lambda_{y}\sin\left(L\Lambda_{y}\right)\\ & & +2L^{2}{\Lambda_{y}}^{2}\Omega_{y}\left(2\sin{\left(\frac{L\Lambda_{y}}{2}\right)}^{2}-1\right)) \end{array}$$

(63) $$\begin{array}{ccc} \varphi_{z}\; & = & 2\left(\Omega_{z}L^{2}{\Lambda_{z}}^{2}+1\right)(L^{2}{\Lambda_{z}}^{2}+4\sin{\left(\frac{L\Lambda_{z}}{2}\right)}^{2}-2L\Lambda_{z}\sin\left(L\Lambda_{z}\right)\\ & & -L^{2}{\Lambda_{z}}^{2}\sin{\left(\frac{L\Lambda_{z}}{2}\right)}^{2}-L^{3}{\Lambda_{z}}^{3}\Omega_{z}\sin\left(L\Lambda_{z}\right)+4L^{2}{\Lambda_{z}}^{2}\Omega_{z}\sin{\left(\frac{L\Lambda_{z}}{2}\right)}^{2}) \end{array}$$

(64) $$\begin{array}{ccc} \phi_{z}\; & = & (4\sin{\left(\frac{L\Lambda_{z}}{2}\right)}^{2}+2L^{2}{\Lambda_{z}}^{2}\Omega_{z}-L\Lambda_{z}\sin\left(L\Lambda_{z}\right)\\ & & +2L^{2}{\Lambda_{z}}^{2}\Omega_{z}\left(2\sin{\left(\frac{L\Lambda_{z}}{2}\right)}^{2}-1\right)) \end{array}$$

(65) $$\begin{array}{ccc} D & = & \left(4\sin{\left(\frac{L\Lambda_{y}}{2}\right)}^{2}-L\Lambda_{y}\sin\left(L\Lambda_{y}\right)+4L^{2}{\Lambda_{y}}^{2}\Omega_{y}\sin{\left(\frac{L\Lambda_{y}}{2}\right)}^{2}\right)(4\sin{\left(\frac{L\Lambda_{z}}{2}\right)}^{2}\\ & & -L\Lambda_{z}\sin\left(L\Lambda_{z}\right)+4L^{2}{\Lambda_{z}}^{2}\Omega_{z}\sin{\left(\frac{L\Lambda_{z}}{2}\right)}^{2}) \end{array}$$

(66) $$\begin{array}{ccc} \psi_{y} & = & {\Lambda_{z}}^{2}(4\Lambda_{y}\left(\sin\left(L\Lambda_{y}\right)-2\cos\left(\frac{L\Lambda_{y}}{2}\right)\cos{\left(\frac{L\Lambda_{z}}{2}\right)}^{2}\sin\left(\frac{L\Lambda_{y}}{2}\right)\right)\\ & & -4L{\Lambda_{y}}^{2}\left(\cos{\left(\frac{L\Lambda_{y}}{2}\right)}^{2}-\cos{\left(\frac{L\Lambda_{y}}{2}\right)}^{2}\cos{\left(\frac{L\Lambda_{z}}{2}\right)}^{2}\right)\\ & & +4L^{2}{\Lambda_{y}}^{3}\left(\Omega_{y}\sin\left(L\Lambda_{y}\right)-2\Omega_{y}\cos\left(\frac{L\Lambda_{y}}{2}\right)\cos{\left(\frac{L\Lambda_{z}}{2}\right)}^{2}\sin\left(\frac{L\Lambda_{y}}{2}\right)\right))\\ & & -{\Lambda_{z}}^{3}(4\left(\sin\left(L\Lambda_{z}\right)-2\cos{\left(\frac{L\Lambda_{y}}{2}\right)}^{2}\cos\left(\frac{L\Lambda_{z}}{2}\right)\sin\left(\frac{L\Lambda_{z}}{2}\right)\right)\\ & & +4L^{2}{\Lambda_{y}}^{2}\left(\Omega_{y}\sin\left(L\Lambda_{z}\right)-2\Omega_{y}\cos{\left(\frac{L\Lambda_{y}}{2}\right)}^{2}\cos\left(\frac{L\Lambda_{z}}{2}\right)\sin\left(\frac{L\Lambda_{z}}{2}\right)\right)\\ & & -L\Lambda_{y}\sin\left(L\Lambda_{y}\right)\sin\left(L\Lambda_{z}\right)) \end{array}$$

(67) $$\begin{array}{ccc} \psi_{z} & = & {\Lambda_{y}}^{2}(4\Lambda_{z}\left(\sin\left(L\Lambda_{z}\right)-2\cos{\left(\frac{L\Lambda_{y}}{2}\right)}^{2}\cos\left(\frac{L\Lambda_{z}}{2}\right)\sin\left(\frac{L\Lambda_{z}}{2}\right)\right)\\ & & -4L{\Lambda_{z}}^{2}\left(\cos{\left(\frac{L\Lambda_{z}}{2}\right)}^{2}-\cos{\left(\frac{L\Lambda_{y}}{2}\right)}^{2}\cos{\left(\frac{L\Lambda_{z}}{2}\right)}^{2}\right)\\ & & +4L^{2}{\Lambda_{z}}^{3}\left(\Omega_{z}\sin\left(L\Lambda_{z}\right)-2\Omega_{z}\cos{\left(\frac{L\Lambda_{y}}{2}\right)}^{2}\cos\left(\frac{L\Lambda_{z}}{2}\right)\sin\left(\frac{L\Lambda_{z}}{2}\right)\right))\\ & & -\;{\Lambda_{y}}^{3}(4\left(\sin\left(L\Lambda_{y}\right)-2\cos\left(\frac{L\Lambda_{y}}{2}\right)\cos{\left(\frac{L\Lambda_{z}}{2}\right)}^{2}\sin\left(\frac{L\Lambda_{y}}{2}\right)\right)\\ & & +4L^{2}{\Lambda_{z}}^{2}\left(\Omega_{z}\sin\left(L\Lambda_{y}\right)-2\Omega_{z}\cos\left(\frac{L\Lambda_{y}}{2}\right)\cos{\left(\frac{L\Lambda_{z}}{2}\right)}^{2}\sin\left(\frac{L\Lambda_{y}}{2}\right)\right)\\ & & -L\Lambda_{z}\sin\left(L\Lambda_{y}\right)\sin\left(L\Lambda_{z}\right)) \end{array}$$

(68) $$\begin{array}{ccc} \psi_{1} & = & \sin\left(\frac{L\;\Lambda_{z}}{2}\right)\;(2\;L\;\;\left({\Lambda_{y}}^{2}-{\Lambda_{z}}^{2}\right)\;\left(2\;\Lambda_{z}\;\cos\left(\frac{L\;\Lambda_{z}}{2}\right)-2\;\Lambda_{z}\;\cos{\left(\frac{L\;\Lambda_{y}}{2}\right)}^{2}\;\cos\left(\frac{L\;\Lambda_{z}}{2}\right)\right)\\ & & -2\;L^{3}\;{\Lambda_{y}}^{2}\;{\Lambda_{z}}^{2}\;\;\left(2\;\Lambda_{z}\;\cos\left(\frac{L\;\Lambda_{z}}{2}\right)-2\;\Lambda_{z}\;\cos{\left(\frac{L\;\Lambda_{y}}{2}\right)}^{2}\;\cos\left(\frac{L\;\Lambda_{z}}{2}\right)\right)\;\left(\Omega_{y}-\Omega_{z}\right))\\ & & -\sin\left(\frac{L\;\Lambda_{y}}{2}\right)\;(2\;L\;\left({\Lambda_{y}}^{2}-{\Lambda_{z}}^{2}\right)\;\left(2\;\Lambda_{y}\;\cos\left(\frac{L\;\Lambda_{y}}{2}\right)-2\;\Lambda_{y}\;\cos\left(\frac{L\;\Lambda_{y}}{2}\right)\;\cos{\left(\frac{L\;\Lambda_{z}}{2}\right)}^{2}\right)\\ & & -2\;L^{3}\;{\Lambda_{y}}^{2}\;{\Lambda_{z}}^{2}\;\;\left(2\;\Lambda_{y}\;\cos\left(\frac{L\;\Lambda_{y}}{2}\right)-2\;\Lambda_{y}\;\cos\left(\frac{L\;\Lambda_{y}}{2}\right)\;\cos{\left(\frac{L\;\Lambda_{z}}{2}\right)}^{2}\right)\;\left(\Omega_{y}-\Omega_{z}\right))\\ & & +\frac{L^{2}\;\Lambda_{y}\;\Lambda_{z}\;\;\left({\Lambda_{y}}^{2}\;\sin\left(L\;\Lambda_{y}\right)\;\sin\left(L\;\Lambda_{z}\right)-4\;\Lambda_{y}\;\Lambda_{z}\;\cos{\left(\frac{L\;\Lambda_{z}}{2}\right)}^{2}-4\;\Lambda_{y}\;\Lambda_{z}\;\cos{\left(\frac{L\;\Lambda_{y}}{2}\right)}^{2}+{\Lambda_{z}}^{2}\;\sin\left(L\;\Lambda_{y}\right)\;\sin\left(L\;\Lambda_{z}\right)+8\;\Lambda_{y}\;\Lambda_{z}\;\cos{\left(\frac{L\;\Lambda_{y}}{2}\right)}^{2}\;\cos{\left(\frac{L\;\Lambda_{z}}{2}\right)}^{2}\right)}{2} \end{array}$$

(69) $$\begin{array}{ccc} \psi_{2} & = & 4{\Lambda_{y}}^{2}\sin{\left(\frac{L\Lambda_{y}}{2}\right)}^{2}\sin{\left(\frac{L\Lambda_{z}}{2}\right)}^{2}-4{\Lambda_{z}}^{2}\sin{\left(\frac{L\Lambda_{y}}{2}\right)}^{2}\sin{\left(\frac{L\Lambda_{z}}{2}\right)}^{2}+L^{2}{\Lambda_{y}}^{2}{\Lambda_{z}}^{2}\sin{\left(\frac{L\Lambda_{y}}{2}\right)}^{2}\\ & & -L^{2}{\Lambda_{y}}^{2}{\Lambda_{z}}^{2}\sin{\left(\frac{L\Lambda_{z}}{2}\right)}^{2}+4L^{2}{\Lambda_{y}}^{4}\Omega_{y}\sin{\left(\frac{L\Lambda_{y}}{2}\right)}^{2}\sin{\left(\frac{L\Lambda_{z}}{2}\right)}^{2}\\ & & -4L^{2}{\Lambda_{z}}^{4}\Omega_{z}\sin{\left(\frac{L\Lambda_{y}}{2}\right)}^{2}\sin{\left(\frac{L\Lambda_{z}}{2}\right)}^{2}+2L\Lambda_{y}{\Lambda_{z}}^{2}\sin\left(L\Lambda_{y}\right)\sin{\left(\frac{L\Lambda_{z}}{2}\right)}^{2}\\ & & -2L{\Lambda_{y}}^{2}\Lambda_{z}\sin\left(L\Lambda_{z}\right)\sin{\left(\frac{L\Lambda_{y}}{2}\right)}^{2}-4L^{2}{\Lambda_{y}}^{2}{\Lambda_{z}}^{2}\Omega_{y}\sin{\left(\frac{L\Lambda_{y}}{2}\right)}^{2}\sin{\left(\frac{L\Lambda_{z}}{2}\right)}^{2}\\ & & +4L^{2}{\Lambda_{y}}^{2}{\Lambda_{z}}^{2}\Omega_{z}\sin{\left(\frac{L\Lambda_{y}}{2}\right)}^{2}\sin{\left(\frac{L\Lambda_{z}}{2}\right)}^{2}-L^{3}{\Lambda_{y}}^{4}\Lambda_{z}\Omega_{y}\sin\left(L\Lambda_{z}\right)\sin{\left(\frac{L\Lambda_{y}}{2}\right)}^{2}\\ & & +L^{3}\Lambda_{y}{\Lambda_{z}}^{4}\Omega_{z}\sin\left(L\Lambda_{y}\right)\sin{\left(\frac{L\Lambda_{z}}{2}\right)}^{2}+L^{3}{\Lambda_{y}}^{3}{\Lambda_{z}}^{2}\Omega_{y}\sin\left(L\Lambda_{y}\right)\sin{\left(\frac{L\Lambda_{z}}{2}\right)}^{2}\\ & & +L^{3}{\Lambda_{y}}^{2}{\Lambda_{z}}^{3}\Omega_{z}\sin\left(L\Lambda_{z}\right)\sin{\left(\frac{L\Lambda_{y}}{2}\right)}^{2}-4L^{4}{\Lambda_{y}}^{2}{\Lambda_{z}}^{4}\Omega_{y}\Omega_{z}\sin{\left(\frac{L\Lambda_{y}}{2}\right)}^{2}\sin{\left(\frac{L\Lambda_{z}}{2}\right)}^{2}\\ & & +4L^{4}{\Lambda_{y}}^{4}{\Lambda_{z}}^{2}\Omega_{y}\Omega_{z}\sin{\left(\frac{L\Lambda_{y}}{2}\right)}^{2}\sin{\left(\frac{L\Lambda_{z}}{2}\right)}^{2} \end{array}$$

**Taylor Series Expansion**

The tangent stiffness matrix developed using complete expressions with hyperbolic and trigonometric functions (TBT_Large_complete) provides accurate results for nonlinear analysis and for buckling load prediction to spatial structures. However, the hyperbolic and trigonometric functions might be computationally inefficient. In addition, in some cases, mainly for initials loads steps in a nonlinear code and for reduced axial loads, the algorithm can present numerical instability. To improve computation efficiency and avoid numerical problems, a Taylor series expansion is proposed, which generates polynomial functions. Furthermore, the user can select the number of terms in the Taylor expansion according to:

**TBT_Large_2tr** – Timoshenko beam theory, 2 terms in tangent stiffness matrix (1 elastic + 1 geometric). The geometric term corresponds to factor that multiplies the coefficient *P*. Conventional formulation (Rodrigues et al. [10])

**TBT_Large_3tr** – Timoshenko beam theory, 3 terms in tangent stiffness matrix (1 elastic + 2 geometric). The geometric term corresponds to factor that multiplies the coefficients *P* and *P².*

**TBT_Large_4tr** – Timoshenko beam theory, 4 terms in tangent stiffness matrix (1 elastic + 3 geometric). The geometric term corresponds to factor that multiplies the coefficients *P, P²* and *P³.*

The coefficients of the complete tangent stiffness matrix of a 3D frame element, including the elastic components and the geometric components with all terms of the Taylor expansion, are shown in eq. 70 to 93:

(70) $$K\left(1,1\right)=K\left(7,7\right)=\frac{P}{L}+\frac{EA}{L}=-K\left(1,7\right)=-K\left(7,1\right)$$

(71) $$\begin{array}{ccc} K(2,2) & = & K\left(8,8\right)=\frac{12E\text{Iz}}{L^{3}\left(12\Omega_{y}+1\right)}+\left(\frac{\left(120{\Omega_{y}}^{2}+20\Omega_{y}+1\right)6}{L{\left(12\Omega_{y}+1\right)}^{2}5}+\frac{12\text{Iz}}{AL^{3}{\left(12\Omega_{y}+1\right)}^{2}}\right)P\\ & & +\left(\begin{array}{c} -\frac{L\left(1680{\Omega_{y}}^{2}+180\Omega_{y}+1\right)}{E\text{Iz}{\left(12\Omega_{y}+1\right)}^{3}350}-\frac{24\text{Iz}\Omega_{y}}{E\text{Iz}5AL{\left(12\Omega_{y}+1\right)}^{3}}+\\ +\frac{L\left(907200{\Omega_{y}}^{3}+172800{\Omega_{y}}^{2}+8640\Omega_{y}+45\right)}{E\text{Iz}{\left(12\Omega_{y}+1\right)}^{4}31500} \end{array}\right)P^{2}\\ & & +\left(\begin{array}{c} \frac{L^{3}\left(21772800{\Omega_{y}}^{5}+6480000{\Omega_{y}}^{4}+665280{\Omega_{y}}^{3}+25920{\Omega_{y}}^{2}+252\Omega_{y}+1\right)}{E^{2}Iz^{2}{\left(12\Omega_{y}+1\right)}^{5}21000}+\frac{\text{Iz}L\left(483840{\Omega_{y}}^{4}+97920{\Omega_{y}}^{3}+5376{\Omega_{y}}^{2}+60\Omega_{y}+1\right)}{E^{2}Iz^{2}A{\left(12\Omega_{y}+1\right)}^{5}700}+\\ -\frac{L\left(1814400L^{2}{\Omega_{y}}^{4}+388800L^{2}{\Omega_{y}}^{3}+23040L^{2}{\Omega_{y}}^{2}+240L^{2}\Omega_{y}+L^{2}\right)}{E^{2}Iz^{2}{\left(12\Omega_{y}+1\right)}^{4}31500} \end{array}\right)P^{3}\\ & & =-K(2,8)=-K(8,2) \end{array}$$

(72) $$\begin{array}{ccc} K(3,3) & = & K\left(9,9\right)=\frac{12E\text{Iy}}{L^{3}\left(12\Omega_{z}+1\right)}+\left(\frac{\left(120{\Omega_{z}}^{2}+20\Omega_{z}+1\right)6}{L{\left(12\Omega_{z}+1\right)}^{2}5}+\frac{12\text{Iy}}{AL^{3}{\left(12\Omega_{z}+1\right)}^{2}}\right)P\\ & & +\left(\begin{array}{c} -\frac{L\left(1680{\Omega_{z}}^{2}+180\Omega_{z}+1\right)}{E\text{Iy}{\left(12\Omega_{z}+1\right)}^{3}350}-\frac{24\text{Iy}\Omega_{z}}{E\text{Iy}5AL{\left(12\Omega_{z}+1\right)}^{3}}+\;\frac{L\left(907200{\Omega_{z}}^{3}+172800{\Omega_{z}}^{2}+8640\Omega_{z}+45\right)}{E\text{Iy}{\left(12\Omega_{z}+1\right)}^{4}31500} \end{array}\right)P^{2}\\ & & +\left(\begin{array}{c} \frac{L^{3}\left(21772800{\Omega_{z}}^{5}+6480000{\Omega_{z}}^{4}+665280{\Omega_{z}}^{3}+25920{\Omega_{z}}^{2}+252\Omega_{z}+1\right)}{E^{2}Iy^{2}{\left(12\Omega_{z}+1\right)}^{5}21000}+\frac{\text{Iy}L\left(483840{\Omega_{z}}^{4}+97920{\Omega_{z}}^{3}+5376{\Omega_{z}}^{2}+60\Omega_{z}+1\right)}{E^{2}Iy^{2}A{\left(12\Omega_{z}+1\right)}^{5}700}\\ -\frac{L\left(1814400L^{2}{\Omega_{z}}^{4}+388800L^{2}{\Omega_{z}}^{3}+23040L^{2}{\Omega_{z}}^{2}+240L^{2}\Omega_{z}+L^{2}\right)}{E^{2}Iy^{2}{\left(12\Omega_{z}+1\right)}^{4}31500} \end{array}\right)P^{3}\\ & & =-K(3,9)=-K(9,3) \end{array}$$

(73) $$K\left(4,4\right)=K\left(10,10\right)=\frac{\mathrm{PJ}\text{p}}{\mathrm{AL}}=-K\left(4,10\right)=-K\left(10,4\right)$$

(74) $$\begin{array}{ccc} K(5,5) & = & K\left(11,11\right)=\frac{4E\text{Iy}\left(3\Omega_{z}+1\right)}{L\left(12\Omega_{z}+1\right)}+\left(\frac{L\left(90{\Omega_{z}}^{2}+15\Omega_{z}+1\right)2}{{\left(12\Omega_{z}+1\right)}^{2}15}+\frac{\text{Iy}\left(36{\Omega_{z}}^{2}+6\Omega_{z}+1\right)4}{AL{\left(12\Omega_{z}+1\right)}^{2}}\right)P\\ & & +\left(\begin{array}{c} -\frac{L^{3}\left(907200{\Omega_{z}}^{4}+241920{\Omega_{z}}^{3}+26460{\Omega_{z}}^{2}+1245\Omega_{z}+11\right)}{E\text{Iy}{\left(12\Omega_{z}+1\right)}^{3}3150}+\frac{6L\text{Iy}\Omega_{z}}{E\text{Iy}5A{\left(12\Omega_{z}+1\right)}^{3}}\\ +\frac{L^{3}\left(163296000{\Omega_{z}}^{5}+57153600{\Omega_{z}}^{4}+8391600{\Omega_{z}}^{3}+621000{\Omega_{z}}^{2}+20655\Omega_{z}+165\right)}{E\text{Iy}{\left(12\Omega_{z}+1\right)}^{4}94500} \end{array}\right)P^{2}\\ & & +\left(\begin{array}{c} \frac{L^{5}\left(3919104000{\Omega_{z}}^{7}+1763596800{\Omega_{z}}^{6}+344476800{\Omega_{z}}^{5}+37260000{\Omega_{z}}^{4}+2307960{\Omega_{z}}^{3}+75690{\Omega_{z}}^{2}+1089\Omega_{z}+7\right)}{E^{2}Iy^{2}{\left(12\Omega_{z}+1\right)}^{5}63000}\\ +\frac{\text{Iy}L^{3}\left(2177280{\Omega_{z}}^{5}+1995840{\Omega_{z}}^{4}+371520{\Omega_{z}}^{3}+24696{\Omega_{z}}^{2}+660\Omega_{z}+11\right)}{E^{2}Iy^{2}A{\left(12\Omega_{z}+1\right)}^{5}6300}\\ -\frac{L^{3}\left(326592000L^{2}{\Omega_{z}}^{6}+119750400L^{2}{\Omega_{z}}^{5}+18727200L^{2}{\Omega_{z}}^{4}+1544400L^{2}{\Omega_{z}}^{3}+63630L^{2}{\Omega_{z}}^{2}+1005L^{2}\Omega_{z}+7L^{2}\right)}{E^{2}Iy^{2}{\left(12\Omega_{z}+1\right)}^{4}94500} \end{array}\right)P^{3} \end{array}$$

(75) $$\begin{array}{ccc} K(6,6) & = & K\left(12,12\right)=\frac{4E\text{Iz}\left(3\Omega_{y}+1\right)}{L\left(12\Omega_{y}+1\right)}+\left(\frac{L\left(90{\Omega_{y}}^{2}+15\Omega_{y}+1\right)2}{{\left(12\Omega_{y}+1\right)}^{2}15}+\frac{\text{Iz}\left(36{\Omega_{y}}^{2}+6\Omega_{y}+1\right)4}{AL{\left(12\Omega_{y}+1\right)}^{2}}\right)P\\ & & +\;\left(\begin{array}{c} -\frac{L^{3}\left(907200{\Omega_{y}}^{4}+241920{\Omega_{y}}^{3}+26460{\Omega_{y}}^{2}+1245\Omega_{y}+11\right)}{E\text{Iz}{\left(12\Omega_{y}+1\right)}^{3}3150}+\frac{6L\text{Iy}\Omega_{y}}{E\text{Iz}5A{\left(12\Omega_{y}+1\right)}^{3}}+\\ +\frac{L^{3}\left(163296000{\Omega_{y}}^{5}+57153600{\Omega_{y}}^{4}+8391600{\Omega_{y}}^{3}+621000{\Omega_{y}}^{2}+20655\Omega_{y}+165\right)}{E\text{Iz}{\left(12\Omega_{y}+1\right)}^{4}94500} \end{array}\right)P^{2}\\ & & +\left(\begin{array}{c} \frac{L^{5}\left(3919104000{\Omega_{y}}^{7}+1763596800{\Omega_{y}}^{6}+344476800{\Omega_{y}}^{5}+37260000{\Omega_{y}}^{4}+2307960{\Omega_{y}}^{3}+75690{\Omega_{y}}^{2}+1089\Omega_{y}+7\right)}{E^{2}Iz^{2}{\left(12\Omega_{y}+1\right)}^{5}63000}\\ +\frac{\text{Iz}L^{3}\left(2177280{\Omega_{y}}^{5}+1995840{\Omega_{y}}^{4}+371520{\Omega_{y}}^{3}+24696{\Omega_{y}}^{2}+660\Omega_{y}+11\right)}{E^{2}Iz^{2}A{\left(12\Omega_{y}+1\right)}^{5}6300}+\\ -\frac{L^{3}\left(326592000L^{2}{\Omega_{y}}^{6}+119750400L^{2}{\Omega_{y}}^{5}+18727200L^{2}{\Omega_{y}}^{4}+1544400L^{2}{\Omega_{y}}^{3}+63630L^{2}{\Omega_{y}}^{2}+1005L^{2}\Omega_{y}+7L^{2}\right)}{E^{2}Iz^{2}{\left(12\Omega_{y}+1\right)}^{4}94500} \end{array}\right)P^{3} \end{array}$$

(76) $$K\left(3,4\right)=K\left(4,3\right)=K\left(6,7\right)=K\left(7,6\right)=\frac{\text{Mza}}{L}=-K\left(1,6\right)=-K\left(6,1\right)=-K\left(4,9\right)=-K\left(9,4\right)$$

(77) $$\begin{array}{ccc} K(4,5) & = & K\left(5,4\right)=\frac{\text{Mzb}}{6}-\frac{\text{Mza}}{3}+\left(-\frac{\left(\frac{1680L^{2}}{E\text{Iy}}+\frac{100800L^{2}\Omega_{z}}{E\text{Iy}}\right)\left(\text{Mza}+\text{Mzb}\right)}{604800}\right)P\\ & & +\frac{\left(\text{Mza}+\text{Mzb}\right)\left(\frac{40L^{4}}{E^{2}Iy^{2}}+\frac{100800L^{4}{\Omega_{z}}^{2}}{E^{2}Iy^{2}}+\frac{3360L^{4}\Omega_{z}}{E^{2}Iy^{2}}\right)}{604800}P^{2}\\ & & +\left(-\frac{\left(\text{Mza}+\text{Mzb}\right)\left(\frac{L^{6}}{E^{3}Iy^{3}}+\frac{5040L^{6}{\Omega_{z}}^{2}}{E^{3}Iy^{3}}+\frac{100800L^{6}{\Omega_{z}}^{3}}{E^{3}Iy^{3}}+\frac{120L^{6}\Omega_{z}}{E^{3}Iy^{3}}\right)}{604800}\right)P^{3} \end{array}$$

(78) $$K(9,10)=K\left(10,9\right)=K\left(1,12\right)=K\left(12,1\right)=-\frac{\text{Mzb}}{L}=-K(7,12)=-K(12,7)=-K(3,10)=-K(10,3)$$

(79) $$\begin{array}{ccc} K(10,11) & = & K\left(11,10\right)=\frac{\text{Mza}}{6}-\frac{\text{Mzb}}{3}+\left(-\frac{\left(\frac{1680L^{2}}{E\text{Iy}}+\frac{100800L^{2}\Omega_{z}}{E\text{Iy}}\right)\left(\text{Mza}+\text{Mzb}\right)}{604800}\right)P\\ & & +\;\frac{\left(\text{Mza}+\text{Mzb}\right)\left(\frac{40L^{4}}{E^{2}Iy^{2}}+\frac{100800L^{4}{\Omega_{z}}^{2}}{E^{2}Iy^{2}}+\frac{3360L^{4}\Omega_{z}}{E^{2}Iy^{2}}\right)}{604800}P^{2}\\ & & +\;\left(-\frac{\left(\text{Mza}+\text{Mzb}\right)\left(\frac{L^{6}}{E^{3}Iy^{3}}+\frac{5040L^{6}{\Omega_{z}}^{2}}{E^{3}Iy^{3}}+\frac{100800L^{6}{\Omega_{z}}^{3}}{E^{3}Iy^{3}}+\frac{120L^{6}\Omega_{z}}{E^{3}Iy^{3}}\right)}{604800}\right)P^{3} \end{array}$$

(80) $$K\left(2,4\right)=K\left(4,2\right)=K\left(5,7\right)=K\left(7,5\right)=\frac{\text{Mya}}{L}=-K\left(1,5\right)=-K\left(5,1\right)=-K\left(4,8\right)=-K\left(8,4\right)$$

(81) $$\begin{array}{ccc} K(3,5) & = & K\left(5,3\right)=-\frac{6E\text{Iy}}{L^{2}\left(12\Omega_{z}+1\right)}+\left(-\frac{1}{10{\left(12\Omega_{z}+1\right)}^{2}}-\frac{6\text{Iy}}{AL^{2}{\left(12\Omega_{z}+1\right)}^{2}}\right)P\\ & & +\left(\begin{array}{c} \frac{L^{2}\left(1680{\Omega_{z}}^{2}+180\Omega_{z}+1\right)}{E\text{Iy}{\left(12\Omega_{z}+1\right)}^{3}700}+\frac{12\text{Iy}\Omega_{z}}{E\text{Iy}5A{\left(12\Omega_{z}+1\right)}^{3}}\\ -\frac{L^{2}\left(907200{\Omega_{z}}^{3}+172800{\Omega_{z}}^{2}+8640\Omega_{z}+45\right)}{E\text{Iy}{\left(12\Omega_{z}+1\right)}^{4}63000} \end{array}\right)P^{2}\\ & & +\;\left(\begin{array}{c} -\frac{L^{4}\left(21772800{\Omega_{z}}^{5}+6480000{\Omega_{z}}^{4}+665280{\Omega_{z}}^{3}+25920{\Omega_{z}}^{2}+252\Omega_{z}+1\right)}{E^{2}Iy^{2}{\left(12\Omega_{z}+1\right)}^{5}42000}\\ -\frac{\text{Iy}L^{2}\left(483840{\Omega_{z}}^{4}+97920{\Omega_{z}}^{3}+5376{\Omega_{z}}^{2}+60\Omega_{z}+1\right)}{E^{2}Iy^{2}A{\left(12\Omega_{z}+1\right)}^{5}1400}\\ +\frac{L^{2}\left(1814400L^{2}{\Omega_{z}}^{4}+388800L^{2}{\Omega_{z}}^{3}+23040L^{2}{\Omega_{z}}^{2}+240L^{2}\Omega_{z}+L^{2}\right)}{E^{2}Iy^{2}{\left(12\Omega_{z}+1\right)}^{4}63000} \end{array}\right)P^{3}\\ & = & K(3,11)=K(11,3)=-K(9,11)=-K(11,9)=-K(5,9)=-K(9,5) \end{array}$$

(82) $$\begin{array}{ccc} K(4,6) & = & K\left(6,4\right)=\frac{\text{Mya}}{3}-\frac{\text{Myb}}{6}+\frac{\left(100800L^{2}\Omega_{y}+1680L^{2}\right)\left(\text{Mya}+\text{Myb}\right)}{604800E\text{Iz}}P\\ & & +\left(-\frac{\left(\text{Mya}+\text{Myb}\right)\left(100800L^{4}{\Omega_{y}}^{2}+3360L^{4}\Omega_{y}+40L^{4}\right)}{E^{2}Iz^{2}604800}\right)P^{2}\\ & & +\frac{\left(\text{Mya}+\text{Myb}\right)\left(100800L^{6}{\Omega_{y}}^{3}+5040L^{6}{\Omega_{y}}^{2}+120L^{6}\Omega_{y}+L^{6}\right)}{E^{3}Iz^{3}604800}P^{3} \end{array}$$

(83) $$\begin{array}{ccc} K(6,8) & = & K\left(8,6\right)=-\frac{6E\text{Iz}}{L^{2}\left(12\Omega_{y}+1\right)}+\left(-\frac{1}{10{\left(12\Omega_{y}+1\right)}^{2}}-\frac{6\text{Iz}}{AL^{2}{\left(12\Omega_{y}+1\right)}^{2}}\right)P\\ & & +\left(\begin{array}{c} \frac{L^{2}\left(1680{\Omega_{y}}^{2}+180\Omega_{y}+1\right)}{E\text{Iz}{\left(12\Omega_{y}+1\right)}^{3}700}+\frac{12\text{Iz}\Omega_{y}}{E\text{Iz}5A{\left(12\Omega_{y}+1\right)}^{3}}\\ -\frac{L^{2}\left(907200{\Omega_{y}}^{3}+172800{\Omega_{y}}^{2}+8640\Omega_{y}+45\right)}{E\text{Iz}{\left(12\Omega_{y}+1\right)}^{4}63000} \end{array}\right)P^{2}\\ & & +\left(\begin{array}{c} -\frac{L^{4}\left(21772800{\Omega_{y}}^{5}+6480000{\Omega_{y}}^{4}+665280{\Omega_{y}}^{3}+25920{\Omega_{y}}^{2}+252\Omega_{y}+1\right)}{E^{2}Iz^{2}{\left(12\Omega_{y}+1\right)}^{5}42000}\\ -\frac{\text{Iz}L^{2}\left(483840{\Omega_{y}}^{4}+97920{\Omega_{y}}^{3}+5376{\Omega_{y}}^{2}+60\Omega_{y}+1\right)}{E^{2}Iz^{2}A{\left(12\Omega_{y}+1\right)}^{5}1400}\\ +\frac{L^{2}\left(1814400L^{2}{\Omega_{y}}^{4}+388800L^{2}{\Omega_{y}}^{3}+23040L^{2}{\Omega_{y}}^{2}+240L^{2}\Omega_{y}+L^{2}\right)}{E^{2}Iz^{2}{\left(12\Omega_{y}+1\right)}^{4}63000} \end{array}\right)P^{3}\\ & = & K(8,12)=K(12,8)=-K(2,6)=-K(6,2)=-K(2,12)=-K(12,2) \end{array}$$

(84) $$K(8,10)=K\left(10,8\right)=K\left(1,11\right)=K\left(11,1\right)=-\frac{\text{Myb}}{L}=-K(7,11)=-K(11,7)=-K(2,10)=-K(10,2)$$

(85) $$\begin{array}{ccc} K(10,12) & = & K\left(12,10\right)=\frac{\text{Myb}}{3}-\frac{\text{Mya}}{6}+\frac{\left(100800L^{2}\Omega_{y}+1680L^{2}\right)\left(\text{Mya}+\text{Myb}\right)}{604800E\text{Iz}}P\\ & & +\left(-\frac{\left(\text{Mya}+\text{Myb}\right)\left(100800L^{4}{\Omega_{y}}^{2}+3360L^{4}\Omega_{y}+40L^{4}\right)}{E^{2}Iz^{2}604800}\right)P^{2}\\ & & +\frac{\left(\text{Mya}+\text{Myb}\right)\left(100800L^{6}{\Omega_{y}}^{3}+5040L^{6}{\Omega_{y}}^{2}+120L^{6}\Omega_{y}+L^{6}\right)}{E^{3}Iz^{3}604800}P^{3} \end{array}$$

(86) $$\begin{array}{ccc} K\left(2,5\right) & = & K\left(5,2\right)=\frac{Mx_{2}}{L\;\left(12\;\Omega_{y}+1\right)}+(-\frac{L\;Mx_{2}}{60\;E\;\text{Iy}\;\left(12\;\Omega_{y}+1\right)}\\ & & -\frac{\begin{array}{c} L\;Mx_{2}\;(941525544960000\;E^{5}\;Iy^{3}\;Iz^{2}\;{\Omega_{y}}^{3}+156920924160000\;E^{5}\;Iy^{3}\;Iz^{2}\;{\Omega_{y}}^{2}\\ +6538371840000\;E^{5}\;Iy^{3}\;Iz^{2}\;\Omega_{y}) \end{array}}{E^{6}\;Iy^{3}\;Iz^{3}\;{\left(12\;\Omega_{y}+1\right)}^{4}\;32691859200000})\;P\\ & & +(\frac{L\;Mx_{2}\;(941525544960000\;E^{4}\;Iy^{3}\;\text{Iz}\;L^{2}\;{\Omega_{y}}^{4}+179338199040000\;E^{4}\;Iy^{3}\;\text{Iz}\;L^{2}\;{\Omega_{y}}^{3}+}{E^{6}\;Iy^{3}\;Iz^{3}\;{\left(12\;\Omega_{y}+1\right)}^{4}\;32691859200000}\\ & & \frac{+8966909952000\;E^{4}\;Iy^{3}\;\text{Iz}\;L^{2}\;{\Omega_{y}}^{2}+46702656000\;E^{4}\;Iy^{3}\;\text{Iz}\;L^{2}\;\Omega_{y}+}{E^{6}\;Iy^{3}\;Iz^{3}\;{\left(12\;\Omega_{y}+1\right)}^{4}\;32691859200000}\\ & & \frac{+22417274880000\;E^{4}\;Iy^{2}\;Iz^{2}\;L^{2}\;{\Omega_{y}}^{3}+\;4296644352000\;E^{4}\;Iy^{2}\;Iz^{2}\;L^{2}\;{\Omega_{y}}^{2}+}{E^{6}\;Iy^{3}\;Iz^{3}\;{\left(12\;\Omega_{y}+1\right)}^{4}\;32691859200000}\\ & & \frac{+249080832000\;E^{4}\;Iy^{2}\;Iz^{2}\;L^{2}\;\Omega_{y}+3891888000\;E^{4}\;Iy^{2}\;Iz^{2}\;L^{2}}{E^{6}\;Iy^{3}\;Iz^{3}\;{\left(12\;\Omega_{y}+1\right)}^{4}\;32691859200000}+\frac{Mx_{2}\;\left(40\;E\;\text{Iy}\;L^{4}+1680\;E\;\text{Iy}\;L^{4}\;\Omega_{z}\right)}{100800\;E^{3}\;Iy^{3}\;L\;\left(12\;\Omega_{y}+1\right)})P^{2}\\ & & +(-\frac{Mx_{2}\;\left(1680\;L^{6}\;{\Omega_{z}}^{2}+80\;L^{6}\;\Omega_{z}+L^{6}\right)}{E^{3}\;Iy^{3}\;L\;\left(12\;\Omega_{y}+1\right)\;100800}-\frac{L\;Mx_{2}\;(124540416000\;E^{3}\;Iy^{3}\;L^{4}\;{\Omega_{y}}^{2}+}{32691859200000\;E^{6}\;Iy^{3}\;Iz^{3}\;{\left(12\;\Omega_{y}+1\right)}^{4}}\\ & & \frac{\;+11955879936000\;E^{3}\;Iy^{3}\;L^{4}\;{\Omega_{y}}^{3}+201755473920000\;E^{3}\;Iy^{3}\;L^{4}\;{\Omega_{y}}^{4}+}{32691859200000\;E^{6}\;Iy^{3}\;Iz^{3}\;{\left(12\;\Omega_{y}+1\right)}^{4}}\\ & & \frac{+941525544960000\;E^{3}\;Iy^{3}\;L^{4}\;{\Omega_{y}}^{5}+108108000\;E^{3}\;\text{Iy}\;Iz^{2}\;L^{4}+}{32691859200000\;E^{6}\;Iy^{3}\;Iz^{3}\;{\left(12\;\Omega_{y}+1\right)}^{4}}\\ & & \frac{+43243200\;E^{3}\;Iy^{2}\;\text{Iz}\;L^{4}+518918400\;E^{3}\;Iy^{3}\;L^{4}\;\Omega_{y}+108972864000\;E^{3}\;\text{Iy}\;Iz^{2}\;L^{4}\;{\Omega_{y}}^{2}+}{32691859200000\;E^{6}\;Iy^{3}\;Iz^{3}\;{\left(12\;\Omega_{y}+1\right)}^{4}}\\ & & \frac{+326918592000\;E^{3}\;Iy^{2}\;\text{Iz}\;L^{4}\;{\Omega_{y}}^{2}+560431872000\;E^{3}\;\text{Iy}\;Iz^{2}\;L^{4}\;{\Omega_{y}}^{3}+}{32691859200000\;E^{6}\;Iy^{3}\;Iz^{3}\;{\left(12\;\Omega_{y}+1\right)}^{4}}\\ & & \frac{\;+4857076224000\;E^{3}\;Iy^{2}\;\text{Iz}\;L^{4}\;{\Omega_{y}}^{3}+22417274880000\;E^{3}\;Iy^{2}\;\text{Iz}\;L^{4}\;{\Omega_{y}}^{4}+6486480000\;E^{3}\;\text{Iy}\;Iz^{2}\;L^{4}\;\Omega_{y}+}{32691859200000\;E^{6}\;Iy^{3}\;Iz^{3}\;{\left(12\;\Omega_{y}+1\right)}^{4}}\\ & & \frac{\;+7005398400\;E^{3}\;Iy^{2}\;\text{Iz}\;L^{4}\;\Omega_{y}+3891888000\;E^{3}\;\text{Iy}\;Iz^{2}\;L^{4}\;\Omega_{z}+249080832000\;E^{3}\;\text{Iy}\;Iz^{2}\;L^{4}\;\Omega_{y}\;\Omega_{z}+}{32691859200000\;E^{6}\;Iy^{3}\;Iz^{3}\;{\left(12\;\Omega_{y}+1\right)}^{4}}\\ & & \;\frac{+4296644352000\;E^{3}\;\text{Iy}\;Iz^{2}\;L^{4}\;{\Omega_{y}}^{2}\;\Omega_{z}+22417274880000\;E^{3}\;\text{Iy}\;Iz^{2}\;L^{4}\;{\Omega_{y}}^{3}\;\Omega_{z})}{32691859200000\;E^{6}\;Iy^{3}\;Iz^{3}\;{\left(12\;\Omega_{y}+1\right)}^{4}})P^{3}\\ & & =K\left(8,11\right)=K\left(11,8\right)=-K\left(5,8\right)=-K\left(8,5\right)=-K\left(2,11\right)=-K\left(11,2\right) \end{array}$$

(87) $$\begin{array}{ccc} K(3,6) & = & K\left(6,3\right)=\frac{Mx_{2}}{L\left(12\Omega_{z}+1\right)}+(-\frac{LMx_{2}}{60E\text{Iz}\left(12\Omega_{z}+1\right)}\\ & & -\frac{\begin{array}{c} LMx_{2}(941525544960000E^{5}Iy^{2}Iz^{3}{\Omega_{z}}^{3}+156920924160000E^{5}Iy^{2}Iz^{3}{\Omega_{z}}^{2}\\ +6538371840000E^{5}Iy^{2}Iz^{3}\Omega_{z}) \end{array}}{E^{6}Iy^{3}Iz^{3}{\left(12\Omega_{z}+1\right)}^{4}32691859200000})P\\ & & +(\frac{Mx_{2}\left(40E\text{Iz}L^{4}+1680E\text{Iz}L^{4}\Omega_{y}\right)}{100800E^{3}Iz^{3}L\left(12\Omega_{z}+1\right)}+\frac{LMx_{2}(22417274880000E^{4}Iy^{2}Iz^{2}L^{2}{\Omega_{z}}^{3}+}{E^{6}Iy^{3}Iz^{3}{\left(12\Omega_{z}+1\right)}^{4}32691859200000}\\ & & \frac{+4296644352000E^{4}Iy^{2}Iz^{2}L^{2}{\Omega_{z}}^{2}+249080832000E^{4}Iy^{2}Iz^{2}L^{2}\Omega_{z}+3891888000E^{4}Iy^{2}Iz^{2}L^{2}+}{E^{6}Iy^{3}Iz^{3}{\left(12\Omega_{z}+1\right)}^{4}32691859200000}\\ & & \frac{+941525544960000E^{4}\text{Iy}Iz^{3}L^{2}{\Omega_{z}}^{4}+179338199040000E^{4}\text{Iy}Iz^{3}L^{2}\Omega_{z}+}{E^{6}Iy^{3}Iz^{3}{\left(12\Omega_{z}+1\right)}^{4}32691859200000}\\ & & \frac{+8966909952000E^{4}\text{Iy}Iz^{3}L^{2}{\Omega_{z}}^{2}+46702656000E^{4}\text{Iy}Iz^{3}L^{2}\Omega_{z})}{E^{6}Iy^{3}Iz^{3}{\left(12\Omega_{z}+1\right)}^{4}32691859200000})P^{2}\\ & & +(-\frac{Mx_{2}\left(1680L^{6}{\Omega_{y}}^{2}+80L^{6}\Omega_{y}+L^{6}\right)}{E^{3}Iz^{3}L\left(12\Omega_{z}+1\right)100800}-\frac{LMx_{2}(124540416000E^{3}Iz^{3}L^{4}{\Omega_{z}}^{2}+}{32691859200000E^{6}Iy^{3}Iz^{3}{\left(12\Omega_{z}+1\right)}^{4}}\\ & & \frac{\;+\;11955879936000E^{3}Iz^{3}L^{4}{\Omega_{z}}^{3}\;\;+\;\;201755473920000E^{3}Iz^{3}L^{4}{\Omega_{z}}^{4}\;+\;941525544960000E^{3}Iz^{3}L^{4}{\Omega_{z}}^{5}\;+\;}{32691859200000E^{6}Iy^{3}Iz^{3}{\left(12\Omega_{z}+1\right)}^{4}}\\ & & \frac{+518918400E^{3}Iz^{3}L^{4}\Omega_{z}+326918592000E^{3}\text{Iy}Iz^{2}L^{4}{\Omega_{z}}^{2}+108972864000E^{3}Iy^{2}\text{Iz}L^{4}{\Omega_{z}}^{2}+}{32691859200000E^{6}Iy^{3}Iz^{3}{\left(12\Omega_{z}+1\right)}^{4}}\\ & & \frac{+4857076224000E^{3}\text{Iy}Iz^{2}L^{4}{\Omega_{z}}^{3}\;\;+\;\;560431872000E^{3}Iy^{2}\text{Iz}L^{4}{\Omega_{z}}^{3}\;+\;22417274880000E^{3}\text{Iy}Iz^{2}L^{4}{\Omega_{z}}^{4}\;\;+\;\;}{32691859200000E^{6}Iy^{3}Iz^{3}{\left(12\Omega_{z}+1\right)}^{4}}\\ & & \frac{+3891888000E^{3}Iy^{2}\text{Iz}L^{4}\Omega_{y}+43243200E^{3}\text{Iy}Iz^{2}L^{4}+108108000E^{3}Iy^{2}\text{Iz}L^{4}+}{32691859200000E^{6}Iy^{3}Iz^{3}{\left(12\Omega_{z}+1\right)}^{4}}\\ & & \frac{+7005398400E^{3}\text{Iy}Iz^{2}L^{4}\Omega_{z}+6486480000E^{3}Iy^{2}\text{Iz}L^{4}\Omega_{z}+249080832000E^{3}Iy^{2}\text{Iz}L^{4}\Omega_{y}\Omega_{z}+}{32691859200000E^{6}Iy^{3}Iz^{3}{\left(12\Omega_{z}+1\right)}^{4}}\\ & & \frac{+4296644352000E^{3}Iy^{2}\text{Iz}L^{4}\Omega_{y}{\Omega_{z}}^{2}+22417274880000E^{3}Iy^{2}\text{Iz}L^{4}\Omega_{y}{\Omega_{z}}^{3})}{32691859200000E^{6}Iy^{3}Iz^{3}{\left(12\Omega_{z}+1\right)}^{4}})\\ & & =K\left(9,12\right)=K\left(12,9\right)=-K\left(6,9\right)=-K\left(9,6\right)=-K\left(3,12\right)=-K\left(12,3\right) \end{array}$$

(88) $$\begin{array}{ccc} K\left(6,10\right) & = & K\left(10,6\right)=\frac{\text{Mya}}{6}+\frac{\text{Myb}}{6}+\left(-\frac{\left(100800L^{2}\Omega_{y}+1680L^{2}\right)\left(\text{Mya}+\text{Myb}\right)}{604800E\text{Iz}}\right)P\\ & & +\left(\frac{\left(\text{Mya}+\text{Myb}\right)\left(100800L^{4}{\Omega_{y}}^{2}+3360L^{4}\Omega_{y}+40L^{4}\right)}{E^{2}{\mathrm{Iz}}^{2}604800}\right)P^{2}+\\ & & +\left(-\frac{\left(\text{Mya}+\text{Myb}\right)\left(100800L^{6}{\Omega_{y}}^{3}+5040L^{6}{\Omega_{y}}^{2}+120L^{6}\Omega_{y}+L^{6}\right)}{E^{3}Iz^{3}604800}\right)P^{3}\\ & & =K\left(4,12\right)=K\left(12,4\right) \end{array}$$

(89) $$\begin{array}{ccc} K\left(5,10\right) & = & K\left(10,5\right)=-\frac{\text{Mza}}{6}-\frac{\text{Mzb}}{6}+\frac{\left(\frac{1680L^{2}}{E\text{Iy}}+\frac{100800L^{2}\Omega_{z}}{E\text{Iy}}\right)\left(\text{Mza}+\text{Mzb}\right)}{604800}P\\ & & +\left(-\frac{\left(\text{Mza}+\text{Mzb}\right)\left(\frac{40L^{4}}{E^{2}Iy^{2}}+\frac{100800L^{4}{\Omega_{z}}^{2}}{E^{2}Iy^{2}}+\frac{3360L^{4}\Omega_{z}}{E^{2}Iy^{2}}\right)}{604800}\right)P^{2}\\ & & +\left(\frac{\left(\text{Mza}+\text{Mzb}\right)\left(\frac{L^{6}}{E^{3}{\mathrm{Iy}}^{3}}+\frac{5040L^{6}{\Omega_{z}}^{2}}{E^{3}{\mathrm{Iy}}^{3}}+\frac{100800L^{6}{\Omega_{z}}^{3}}{E^{3}{\mathrm{Iy}}^{3}}+\frac{120L^{6}\Omega_{z}}{E^{3}{\mathrm{Iy}}^{3}}\right)}{604800}\right)P^{3}\\ & & =K\left(4,11\right)=K\left(11,4\right) \end{array}$$

(90) $$\begin{array}{ccc} K\left(5,11\right) & = & \frac{2E\text{Iy}\left(1-6\Omega_{z}\right)}{L\left(12\Omega_{z}+1\right)}+\left(-\frac{L\left(360{\Omega_{z}}^{2}+60\Omega_{z}+1\right)}{{\left(12\Omega_{z}+1\right)}^{2}30}-\frac{\text{Iy}\left(72{\Omega_{z}}^{2}+12\Omega_{z}-1\right)2}{\mathrm{AL}{\left(12\Omega_{z}+1\right)}^{2}}\right)P\\ & & +\left(\begin{array}{c} \frac{L^{3}\left(1814400{\Omega_{z}}^{4}+483840{\Omega_{z}}^{3}+37800{\Omega_{z}}^{2}+870\Omega_{z}+13\right)}{E\text{Iy}{\left(12\Omega_{z}+1\right)}^{3}6300}-\frac{6\text{Iy}L\Omega_{z}}{E\text{Iy}5A{\left(12\Omega_{z}+1\right)}^{3}}+\\ -\frac{L^{3}\left(326592000{\Omega_{z}}^{5}+114307200{\Omega_{z}}^{4}+14061600{\Omega_{z}}^{3}+723600{\Omega_{z}}^{2}+15390\Omega_{z}+195\right)}{E\text{Iy}{\left(12\Omega_{z}+1\right)}^{4}189000} \end{array}\right)P^{2}\\ & & \left(\;\begin{array}{c} -\frac{L^{5}\left(7838208000{\Omega_{z}}^{7}+3527193600{\Omega_{z}}^{6}+623635200{\Omega_{z}}^{5}+55080000{\Omega_{z}}^{4}+2620080{\Omega_{z}}^{3}+73620{\Omega_{z}}^{2}+1422\Omega_{z}+11\right)}{E^{2}Iy^{2}{\left(12\Omega_{z}+1\right)}^{5}126000}+\\ +\frac{\text{Iy}L^{3}\left(4354560{\Omega_{z}}^{5}-362880{\Omega_{z}}^{4}-138240{\Omega_{z}}^{3}+1008{\Omega_{z}}^{2}+780\Omega_{z}+13\right)}{E^{2}Iy^{2}A{\left(12\Omega_{z}+1\right)}^{5}12600}+\\ +\frac{L^{3}\left(653184000L^{2}{\Omega_{z}}^{6}+239500800L^{2}{\Omega_{z}}^{5}+32011200L^{2}{\Omega_{z}}^{4}+1922400L^{2}{\Omega_{z}}^{3}+58140L^{2}{\Omega_{z}}^{2}+1290L^{2}\Omega_{z}+11L^{2}\right)}{E^{2}Iy^{2}{\left(12\Omega_{z}+1\right)}^{4}189000} \end{array}\;\right)P^{3}\\ & & =K\left(11,5\right) \end{array}$$

(91) $$\begin{array}{ccc} K\left(6,12\right) & & =\frac{2E\text{Iz}\left(1-6\Omega_{y}\right)}{L\left(12\Omega_{y}+1\right)}+\left(-\frac{L\left(360{\Omega_{y}}^{2}+60\Omega_{y}+1\right)}{{\left(12\Omega_{y}+1\right)}^{2}30}-\frac{\text{Iz}\left(72{\Omega_{y}}^{2}+12\Omega_{y}-1\right)2}{\mathrm{AL}{\left(12\Omega_{y}+1\right)}^{2}}\right)P\\ & & +\left(\begin{array}{c} \frac{L^{3}\left(1814400{\Omega_{y}}^{4}+483840{\Omega_{y}}^{3}+37800{\Omega_{y}}^{2}+870\Omega_{y}+13\right)}{E\text{Iz}{\left(12\Omega_{y}+1\right)}^{3}6300}-\frac{6\text{Iz}L\Omega_{z}}{E\text{Iz}5A{\left(12\Omega_{y}+1\right)}^{3}}+\\ -\frac{L^{3}\left(326592000{\Omega_{y}}^{5}+114307200{\Omega_{y}}^{4}+14061600{\Omega_{y}}^{3}+723600{\Omega_{y}}^{2}+15390\Omega_{y}+195\right)}{E\text{Iz}{\left(12\Omega_{y}+1\right)}^{4}189000} \end{array}\right)P^{2}\\ & & \left(\begin{array}{c} +-\frac{L^{5}\left(7838208000{\Omega_{y}}^{7}+3527193600{\Omega_{y}}^{6}+623635200{\Omega_{y}}^{5}+55080000{\Omega_{y}}^{4}+2620080{\Omega_{y}}^{3}+73620{\Omega_{y}}^{2}+1422\Omega_{y}+11\right)}{E^{2}Iz^{2}{\left(12\Omega_{y}+1\right)}^{5}126000}+\\ +\frac{\text{Iz}L^{3}\left(4354560{\Omega_{y}}^{5}-362880{\Omega_{y}}^{4}-138240{\Omega_{y}}^{3}+1008{\Omega_{y}}^{2}+780\Omega_{y}+13\right)}{E^{2}Iz^{2}A{\left(12\Omega_{y}+1\right)}^{5}12600}+\\ +\frac{L^{3}\left(653184000L^{2}{\Omega_{y}}^{6}+239500800L^{2}{\Omega_{y}}^{5}+32011200L^{2}{\Omega_{y}}^{4}+1922400L^{2}{\Omega_{y}}^{3}+58140L^{2}{\Omega_{y}}^{2}+1290L^{2}\Omega_{y}+11L^{2}\right)}{E^{2}Iz^{2}{\left(12\Omega_{y}+1\right)}^{4}189000} \end{array}\right)P^{3}\\ & & =K\left(12,6\right) \end{array}$$

(92) $$\begin{array}{ccc} K(6,11) & = & K\left(11,6\right)=\frac{Mx_{2}\left(144\Omega_{y}\Omega_{z}-1\right)}{2\left(12\Omega_{y}+1\right)\left(12\Omega_{z}+1\right)}+\\ & & +\frac{L^{2}Mx_{2}(\text{Iy}+\text{Iz}+144\text{Iy}{\Omega_{y}}^{2}+144\text{Iz}{\Omega_{z}}^{2}+36\text{Iy}\Omega_{y}+12\text{Iy}\Omega_{z}+12\text{Iz}\Omega_{y}+36\text{Iz}\Omega_{z}+}{120E\text{Iy}\text{Iz}{\left(12\Omega_{y}+1\right)}^{2}{\left(12\Omega_{z}+1\right)}^{2}}\\ & & \frac{+576\text{Iy}\Omega_{y}\Omega_{z}\;+\;576\text{Iz}\Omega_{y}\Omega_{z}\;+\;1728\text{Iy}\Omega_{y}{\Omega_{z}}^{2}\;+\;1728\text{Iy}{\Omega_{y}}^{2}\Omega_{z}\;+\;1728\text{Iz}\Omega_{y}{\Omega_{z}}^{2}\;+\;1728\text{Iz}{\Omega_{y}}^{2}\Omega_{z})}{120E\text{Iy}\text{Iz}{\left(12\Omega_{y}+1\right)}^{2}{\left(12\Omega_{z}+1\right)}^{2}}P\\ & & -(\frac{L^{4}Mx_{2}(52254720Iy^{2}{\Omega_{y}}^{4}{\Omega_{z}}^{2}\;+\;8709120Iy^{2}{\Omega_{y}}^{4}\Omega_{z}\;+\;362880Iy^{2}{\Omega_{y}}^{4}\;+\;52254720Iy^{2}{\Omega_{y}}^{3}{\Omega_{z}}^{3}+}{25200E^{2}Iy^{2}Iz^{2}{\left(12\Omega_{y}+1\right)}^{3}{\left(12\Omega_{z}+1\right)}^{3}}\\ & & \frac{+27371520Iy^{2}{\Omega_{y}}^{3}{\Omega_{z}}^{2}+3473280Iy^{2}{\Omega_{y}}^{3}\Omega_{z}+129600Iy^{2}{\Omega_{y}}^{3}+5598720Iy^{2}{\Omega_{y}}^{2}{\Omega_{z}}^{3}+}{25200E^{2}Iy^{2}Iz^{2}{\left(12\Omega_{y}+1\right)}^{3}{\left(12\Omega_{z}+1\right)}^{3}}\\ & & \frac{+2799360Iy^{2}{\Omega_{y}}^{2}{\Omega_{z}}^{2}\;+\;349920Iy^{2}{\Omega_{y}}^{2}\Omega_{z}\;+\;12960Iy^{2}{\Omega_{y}}^{2}\;+\;31104Iy^{2}\Omega_{y}{\Omega_{z}}^{3}\;+\;63936Iy^{2}\Omega_{y}{\Omega_{z}}^{2}+}{25200E^{2}Iy^{2}Iz^{2}{\left(12\Omega_{y}+1\right)}^{3}{\left(12\Omega_{z}+1\right)}^{3}}\\ & & \frac{+10008Iy^{2}\Omega_{y}\Omega_{z}+408Iy^{2}\Omega_{y}+720Iy^{2}{\Omega_{z}}^{2}+120Iy^{2}\Omega_{z}+5Iy^{2}+1244160\text{IyIz}{\Omega_{y}}^{3}{\Omega_{z}}^{2}+}{25200E^{2}Iy^{2}Iz^{2}{\left(12\Omega_{y}+1\right)}^{3}{\left(12\Omega_{z}+1\right)}^{3}}\\ & & \frac{+134784\text{IyIz}{\Omega_{y}}^{3}\Omega_{z}+2592\text{IyIz}{\Omega_{y}}^{3}+1244160\text{IyIz}{\Omega_{y}}^{2}{\Omega_{z}}^{3}+622080\text{IyIz}{\Omega_{y}}^{2}{\Omega_{z}}^{2}+}{25200E^{2}Iy^{2}Iz^{2}{\left(12\Omega_{y}+1\right)}^{3}{\left(12\Omega_{z}+1\right)}^{3}}\\ & & \frac{+59616\text{IyIz}{\Omega_{y}}^{2}\Omega_{z}+1368\text{IyIz}{\Omega_{y}}^{2}+134784\text{IyIz}\Omega_{y}{\Omega_{z}}^{3}+59616\text{IyIz}\Omega_{y}{\Omega_{z}}^{2}+5616\text{IyIz}\Omega_{y}\Omega_{z}+}{25200E^{2}Iy^{2}Iz^{2}{\left(12\Omega_{y}+1\right)}^{3}{\left(12\Omega_{z}+1\right)}^{3}}\\ & & \frac{+132\text{IyIz}\Omega_{y}+2529\text{IyIz}{\Omega_{z}}^{3}+1368\text{IyIz}{\Omega_{z}}^{2}+132\text{IyIz}\Omega_{z}+3\text{IyIz}+52254720Iz^{2}{\Omega_{y}}^{3}{\Omega_{z}}^{3}+}{25200E^{2}Iy^{2}Iz^{2}{\left(12\Omega_{y}+1\right)}^{3}{\left(12\Omega_{z}+1\right)}^{3}}\\ & & \frac{5598720Iz^{2}{\Omega_{y}}^{3}{\Omega_{z}}^{2}+31104Iz^{2}{\Omega_{y}}^{3}\Omega_{z}+52254720Iz^{2}{\Omega_{y}}^{2}{\Omega_{z}}^{4}+27371520Iz^{2}{\Omega_{y}}^{2}{\Omega_{z}}^{3}+}{25200E^{2}Iy^{2}Iz^{2}{\left(12\Omega_{y}+1\right)}^{3}{\left(12\Omega_{z}+1\right)}^{3}}\\ & & \frac{+2799360Iz^{2}{\Omega_{y}}^{2}{\Omega_{z}}^{2}\;+\;2529\text{IyIz}{\Omega_{z}}^{3}\;+\;1368\text{IyIz}{\Omega_{z}}^{2}\;+\;132\text{IyIz}\Omega_{z}\;+\;3\text{IyIz}\;+\;52254720Iz^{2}{\Omega_{y}}^{3}{\Omega_{z}}^{3}+}{25200E^{2}Iy^{2}Iz^{2}{\left(12\Omega_{y}+1\right)}^{3}{\left(12\Omega_{z}+1\right)}^{3}}\\ & & \frac{+5598720Iz^{2}{\Omega_{y}}^{3}{\Omega_{z}}^{2}+31104Iz^{2}{\Omega_{y}}^{3}\Omega_{z}+52254720Iz^{2}{\Omega_{y}}^{2}{\Omega_{z}}^{4}+27371520Iz^{2}{\Omega_{y}}^{2}{\Omega_{z}}^{3}+}{25200E^{2}Iy^{2}Iz^{2}{\left(12\Omega_{y}+1\right)}^{3}{\left(12\Omega_{z}+1\right)}^{3}}\\ & & \frac{+2799360Iz^{2}{\Omega_{y}}^{2}{\Omega_{z}}^{2}+63936Iz^{2}{\Omega_{y}}^{2}\Omega_{z}+720Iz^{2}{\Omega_{y}}^{2}+8709120Iz^{2}\Omega_{y}{\Omega_{z}}^{4}+}{25200E^{2}Iy^{2}Iz^{2}{\left(12\Omega_{y}+1\right)}^{3}{\left(12\Omega_{z}+1\right)}^{3}}\\ & & \frac{+3473280Iz^{2}\Omega_{y}{\Omega_{z}}^{3}+349920Iz^{2}\Omega_{y}{\Omega_{z}}^{2}+10008Iz^{2}\Omega_{y}\Omega_{z}+120Iz^{2}\Omega_{y}+362880Iz^{2}{\Omega_{z}}^{4}+}{25200E^{2}Iy^{2}Iz^{2}{\left(12\Omega_{y}+1\right)}^{3}{\left(12\Omega_{z}+1\right)}^{3}}\\ & & \frac{+129600Iz^{2}{\Omega_{z}}^{3}+12960Iz^{2}{\Omega_{z}}^{2}+408Iz^{2}\Omega_{z}+5Iz^{2})}{25200E^{2}Iy^{2}Iz^{2}{\left(12\Omega_{y}+1\right)}^{3}{\left(12\Omega_{z}+1\right)}^{3}})P^{2}\\ & & +(\frac{L^{6}Mx_{2}(648\Omega_{z}+23040\Omega_{y}{\Omega_{z}}^{2}+2211840\Omega_{y}{\Omega_{z}}^{3}+37324800\Omega_{y}{\Omega_{z}}^{4}+174182400\Omega_{y}{\Omega_{z}}^{5}+}{1008000E^{3}Iy^{3}\left(12\Omega_{y}+1\right){\left(12\Omega_{z}+1\right)}^{4}}\\ & & \frac{+33840{\Omega_{z}}^{2}\;+\;967680{\Omega_{z}}^{3}\;+\;13236480{\Omega_{z}}^{4}\;+\;80870400{\Omega_{z}}^{5}\;+\;174182400{\Omega_{z}}^{6}\;+\;96\Omega_{y}\Omega_{z}+5)}{1008000E^{3}Iy^{3}\left(12\Omega_{y}+1\right){\left(12\Omega_{z}+1\right)}^{4}}+\\ & & \frac{L^{6}Mx_{2}(648\Omega_{y}+23040{\Omega_{y}}^{2}\Omega_{z}+2211840{\Omega_{y}}^{3}\Omega_{z}+37324800{\Omega_{y}}^{4}\Omega_{z}+174182400{\Omega_{y}}^{5}\Omega_{z}+}{1008000E^{3}Iz^{3}{\left(12\Omega_{y}+1\right)}^{4}\left(12\Omega_{z}+1\right)}\\ & & \frac{+33840{\Omega_{y}}^{2}+967680{\Omega_{y}}^{3}+13236480{\Omega_{y}}^{4}+80870400{\Omega_{y}}^{5}+174182400{\Omega_{y}}^{6}+96\Omega_{y}\Omega_{z}+5)}{1008000E^{3}Iz^{3}{\left(12\Omega_{y}+1\right)}^{4}\left(12\Omega_{z}+1\right)}\\ & & +\frac{L^{6}Mx_{2}(12441600{\Omega_{y}}^{4}\Omega_{z}\;+\;311040{\Omega_{y}}^{4}\;+\;12441600{\Omega_{y}}^{3}{\Omega_{z}}^{2}\;+\;5495040{\Omega_{y}}^{3}\Omega_{z}+172800{\Omega_{y}}^{3}+}{3024000E^{3}\text{Iy}Iz^{2}{\left(12\Omega_{y}+1\right)}^{3}{\left(12\Omega_{z}+1\right)}^{2}}\\ & & \frac{+1658880{\Omega_{y}}^{2}{\Omega_{z}}^{2}\;+\;613440{\Omega_{y}}^{2}\Omega_{z}\;+\;20160{\Omega_{y}}^{2}\;+\;43200\Omega_{y}{\Omega_{z}}^{2}+20880\Omega_{y}\Omega_{z}+660\Omega_{y}+}{3024000E^{3}\text{Iy}Iz^{2}{\left(12\Omega_{y}+1\right)}^{3}{\left(12\Omega_{z}+1\right)}^{2}}\\ & & \frac{+288{\Omega_{z}}^{2}+7)}{3024000E^{3}\text{Iy}Iz^{2}{\left(12\Omega_{y}+1\right)}^{3}{\left(12\Omega_{z}+1\right)}^{2}}+\frac{L^{6}Mx_{2}(12441600{\Omega_{y}}^{2}{\Omega_{z}}^{3}+}{3024000E^{3}Iy^{2}\text{Iz}{\left(12\Omega_{y}+1\right)}^{2}{\left(12\Omega_{z}+1\right)}^{3}}\\ & & \frac{+1658880{\Omega_{y}}^{2}{\Omega_{z}}^{2}+43200{\Omega_{y}}^{2}\Omega_{z}+288{\Omega_{y}}^{2}+12441600\Omega_{y}{\Omega_{z}}^{4}+5495040\Omega_{y}{\Omega_{z}}^{3}+}{3024000E^{3}Iy^{2}\text{Iz}{\left(12\Omega_{y}+1\right)}^{2}{\left(12\Omega_{z}+1\right)}^{3}}\\ \; & & \frac{+613440\Omega_{y}{\Omega_{z}}^{2}+20880\Omega_{y}\Omega_{z}+228\Omega_{y}+311040{\Omega_{z}}^{4}+172800{\Omega_{z}}^{3}+660\Omega_{z}+7)}{3024000E^{3}Iy^{2}\text{Iz}{\left(12\Omega_{y}+1\right)}^{2}{\left(12\Omega_{z}+1\right)}^{3}})P^{3}\\ & & =-K(5,12)=-K(12,5) \end{array}$$

$$\begin{array}{ccc} K(5,6) & = & K\left(6,5\right)=-\frac{6Mx_{2}\left(\Omega_{y}-\Omega_{z}\right)}{\left(12\Omega_{y}+1\right)\left(12\Omega_{z}+1\right)}+\frac{L^{2}Mx_{2}(\text{Iy}-\text{Iz}+144\text{Iy}{\Omega_{y}}^{2}-144\text{Iz}{\Omega_{z}}^{2}+12\text{Iy}\Omega_{y}+}{120E\text{Iy}\text{Iz}{\left(12\Omega_{y}+1\right)}^{2}{\left(12\Omega_{z}+1\right)}^{2}}\\ & & \frac{+12\text{Iy}\Omega_{z}\;-\;12\text{Iz}\Omega_{y}\;-\;12\text{Iz}\Omega_{z}\;-\;1728\text{Iy}\Omega_{y}{\Omega_{z}}^{2}\;+\;1728\text{Iy}{\Omega_{y}}^{2}\Omega_{z}\;-\;1728\text{Iz}\Omega_{y}{\Omega_{z}}^{2}+1728\text{Iz}{\Omega_{y}}^{2}\Omega_{z})}{120E\text{Iy}\text{Iz}{\left(12\Omega_{y}+1\right)}^{2}{\left(12\Omega_{z}+1\right)}^{2}}P\\ & & -(\frac{L^{4}Mx_{2}(52254720Iy^{2}{\Omega_{y}}^{4}{\Omega_{z}}^{2}\;+\;8709120Iy^{2}{\Omega_{y}}^{4}\Omega_{z}\;+\;362880Iy^{2}{\Omega_{y}}^{4}\;-\;52254720Iy^{2}{\Omega_{y}}^{3}{\Omega_{z}}^{3}+}{25200E^{2}Iy^{2}Iz^{2}{\left(12\Omega_{y}+1\right)}^{3}{\left(12\Omega_{z}+1\right)}^{3}}\\ & & \frac{+1244160Iy^{2}{\Omega_{y}}^{3}{\Omega_{z}}^{2}+1296000Iy^{2}{\Omega_{y}}^{3}\Omega_{z}+69120Iy^{2}{\Omega_{y}}^{3}-5598720Iy^{2}{\Omega_{y}}^{2}{\Omega_{z}}^{3}+}{25200E^{2}Iy^{2}Iz^{2}{\left(12\Omega_{y}+1\right)}^{3}{\left(12\Omega_{z}+1\right)}^{3}}\\ & & \frac{+116640Iy^{2}{\Omega_{y}}^{2}\Omega_{z}+6480Iy^{2}{\Omega_{y}}^{2}-31104Iy^{2}\Omega_{y}{\Omega_{z}}^{3}+48384Iy^{2}\Omega_{y}{\Omega_{z}}^{2}+8712Iy^{2}\Omega_{y}\Omega_{z}+}{25200E^{2}Iy^{2}Iz^{2}{\left(12\Omega_{y}+1\right)}^{3}{\left(12\Omega_{z}+1\right)}^{3}}\\ & & \frac{+372Iy^{2}\Omega_{y}+720Iy^{2}{\Omega_{z}}^{2}+120Iy^{2}\Omega_{z}+5Iy^{2}+1244160\text{IyIz}{\Omega_{y}}^{3}{\Omega_{z}}^{2}+134784\text{IyIz}{\Omega_{y}}^{3}\Omega_{z}+}{25200E^{2}Iy^{2}Iz^{2}{\left(12\Omega_{y}+1\right)}^{3}{\left(12\Omega_{z}+1\right)}^{3}}\\ & & \frac{+2592\text{IyIz}{\Omega_{y}}^{3}-1244160\text{IyIz}{\Omega_{y}}^{2}{\Omega_{z}}^{3}+7776\text{IyIz}{\Omega_{y}}^{2}\Omega_{z}-72\text{IyIz}{\Omega_{y}}^{2}-134784\text{IyIz}\Omega_{y}{\Omega_{z}}^{3}-}{25200E^{2}Iy^{2}Iz^{2}{\left(12\Omega_{y}+1\right)}^{3}{\left(12\Omega_{z}+1\right)}^{3}}\\ & & \frac{-7776\text{IyIz}\Omega_{y}{\Omega_{z}}^{2}-24\text{IyIz}\Omega_{y}-2592\text{IyIz}{\Omega_{z}}^{3}+72\text{IyIz}{\Omega_{z}}^{2}+24\text{IyIz}\Omega_{z}+52254720Iz^{2}{\Omega_{y}}^{3}{\Omega_{z}}^{3}+}{25200E^{2}Iy^{2}Iz^{2}{\left(12\Omega_{y}+1\right)}^{3}{\left(12\Omega_{z}+1\right)}^{3}}\\ & & \frac{+5598720Iz^{2}{\Omega_{y}}^{3}{\Omega_{z}}^{2}+31104Iz^{2}{\Omega_{y}}^{3}\Omega_{z}-52254720Iz^{2}{\Omega_{y}}^{2}{\Omega_{z}}^{4}-1244160Iz^{2}{\Omega_{y}}^{2}{\Omega_{z}}^{3}-}{25200E^{2}Iy^{2}Iz^{2}{\left(12\Omega_{y}+1\right)}^{3}{\left(12\Omega_{z}+1\right)}^{3}}\\ & & \frac{-48384Iz^{2}{\Omega_{y}}^{2}\Omega_{z}-720Iz^{2}{\Omega_{y}}^{2}-8709120Iz^{2}\Omega_{y}{\Omega_{z}}^{4}-1296000Iz^{2}\Omega_{y}{\Omega_{z}}^{3}-116640Iz^{2}\Omega_{y}{\Omega_{z}}^{2}+}{25200E^{2}Iy^{2}Iz^{2}{\left(12\Omega_{y}+1\right)}^{3}{\left(12\Omega_{z}+1\right)}^{3}}\\ & & \frac{-8712Iz^{2}\Omega_{y}\;-\;120Iz^{2}\Omega_{y}\;-\;362880Iz^{2}{\Omega_{z}}^{4}\;-\;69120Iz^{2}\Omega_{y}{\Omega_{z}}^{3}\;-\;6480Iz^{2}{\Omega_{z}}^{2}\;-\;372Iz^{2}\Omega_{z}\;-\;5Iz^{2}}{25200E^{2}Iy^{2}Iz^{2}{\left(12\Omega_{y}+1\right)}^{3}{\left(12\Omega_{z}+1\right)}^{3}})P^{2}\\ & & +(\frac{\text{Iy}L^{6}(49766400{\Omega_{y}}^{4}{\Omega_{z}}^{2}+5391360{\Omega_{y}}^{4}\Omega_{z}+103680{\Omega_{y}}^{4}-49766400{\Omega_{y}}^{3}{\Omega_{z}}^{3}+}{1008000E^{3}Iy^{2}Iz^{2}{\left(12\Omega_{y}+1\right)}^{3}{\left(12\Omega_{z}+1\right)}^{3}}\\ & & \frac{+1244160{\Omega_{y}}^{3}{\Omega_{z}}^{2}\;+\;449280{\Omega_{y}}^{3}\Omega_{z}\;-\;6635520{\Omega_{y}}^{2}{\Omega_{z}}^{3}\;-\;311040{\Omega_{y}}^{2}{\Omega_{z}}^{2}\;+\;8640{\Omega_{y}}^{2}\Omega_{z}\;-\;960{\Omega_{y}}^{2}\;-\;}{1008000E^{3}Iy^{2}Iz^{2}{\left(12\Omega_{y}+1\right)}^{3}{\left(12\Omega_{z}+1\right)}^{3}}\\ & & \frac{-172800\Omega_{y}{\Omega_{z}}^{3}+11520\Omega_{y}{\Omega_{z}}^{2}+2400\Omega_{y}\Omega_{z}+20\Omega_{y}-1152{\Omega_{z}}^{3}+432{\Omega_{z}}^{2}+56\Omega_{z}+1)}{1008000E^{3}Iy^{2}Iz^{2}{\left(12\Omega_{y}+1\right)}^{3}{\left(12\Omega_{z}+1\right)}^{3}} \end{array}$$

(93) $$\begin{array}{ccc} \; & & -\frac{\text{Iz}L^{6}(-49766400{\Omega_{y}}^{3}{\Omega_{z}}^{3}-6635520{\Omega_{y}}^{3}{\Omega_{z}}^{2}-172800{\Omega_{y}}^{3}\Omega_{z}-1152{\Omega_{y}}^{3}}{1008000E^{3}Iy^{2}Iz^{2}{\left(12\Omega_{y}+1\right)}^{3}{\left(12\Omega_{z}+1\right)}^{3}}\\ & & \frac{+49766400{\Omega_{y}}^{2}{\Omega_{z}}^{4}+1244160{\Omega_{y}}^{2}{\Omega_{z}}^{3}+}{1008000E^{3}Iy^{2}Iz^{2}{\left(12\Omega_{y}+1\right)}^{3}{\left(12\Omega_{z}+1\right)}^{3}}\\ & & \frac{-311040{\Omega_{y}}^{2}{\Omega_{z}}^{2}+11520{\Omega_{y}}^{2}\Omega_{z}+432{\Omega_{y}}^{2}+5391360\Omega_{y}{\Omega_{z}}^{4}}{1008000E^{3}Iy^{2}Iz^{2}{\left(12\Omega_{y}+1\right)}^{3}{\left(12\Omega_{z}+1\right)}^{3}}\\ & & \frac{+449280\Omega_{y}{\Omega_{z}}^{3}+8640\Omega_{y}{\Omega_{z}}^{2}+2400\Omega_{y}\Omega_{z}+56\Omega_{y}+103680{\Omega_{z}}^{4}-960{\Omega_{z}}^{2}+20\Omega_{z}+1)}{1008000E^{3}Iy^{2}Iz^{2}{\left(12\Omega_{y}+1\right)}^{3}{\left(12\Omega_{z}+1\right)}^{3}}\\ & & +\frac{L^{6}(632\Omega_{y}-23040{\Omega_{y}}^{2}\Omega_{z}-2211840{\Omega_{y}}^{3}\Omega_{z}+}{1008000E^{3}Iz^{3}{\left(12\Omega_{y}+1\right)}^{4}\left(12\Omega_{z}+1\right)}\\ & & -\frac{37324800{\Omega_{y}}^{4}\Omega_{z}-174182400{\Omega_{y}}^{5}\Omega_{z}+30000{\Omega_{y}}^{2}+599040{\Omega_{y}}^{3}+7015680{\Omega_{y}}^{4}+}{1008000E^{3}Iz^{3}{\left(12\Omega_{y}+1\right)}^{4}\left(12\Omega_{z}+1\right)}\\ & & \frac{+51840000{\Omega_{y}}^{5}+174182400{\Omega_{y}}^{6}-96\Omega_{y}\Omega_{z}+5)}{1008000E^{3}Iz^{3}{\left(12\Omega_{y}+1\right)}^{4}\left(12\Omega_{z}+1\right)}\\ & & -\frac{L^{6}(-25082265600{\Omega_{y}}^{3}{\Omega_{z}}^{5}-5374771200{\Omega_{y}}^{3}{\Omega_{z}}^{4}+}{1008000E^{3}Iy^{3}{\left(12\Omega_{y}+1\right)}^{3}{\left(12\Omega_{z}+1\right)}^{4}}\\ & & \frac{-318504960{\Omega_{y}}^{3}{\Omega_{z}}^{3}-3317760{\Omega_{y}}^{3}{\Omega_{z}}^{2}-13824{\Omega_{y}}^{3}\Omega_{z}+25082265600{\Omega_{y}}^{2}{\Omega_{z}}^{6}+}{1008000E^{3}Iy^{3}{\left(12\Omega_{y}+1\right)}^{3}{\left(12\Omega_{z}+1\right)}^{4}}\\ & & \frac{+3284582400{\Omega_{y}}^{2}{\Omega_{z}}^{5}+114462720{\Omega_{y}}^{2}{\Omega_{z}}^{4}+33177600{\Omega_{y}}^{2}{\Omega_{z}}^{3}+3767040{\Omega_{y}}^{2}{\Omega_{z}}^{2}+}{1008000E^{3}Iy^{3}{\left(12\Omega_{y}+1\right)}^{3}{\left(12\Omega_{z}+1\right)}^{4}}\\ & & \frac{+88704{\Omega_{y}}^{2}\Omega_{z}+720{\Omega_{y}}^{2}+4180377600\Omega_{y}{\Omega_{z}}^{6}+1069977600\Omega_{y}{\Omega_{z}}^{5}+131051520\Omega_{y}{\Omega_{z}}^{4}+}{1008000E^{3}Iy^{3}{\left(12\Omega_{y}+1\right)}^{3}{\left(12\Omega_{z}+1\right)}^{4}}\\ & & \frac{+12165120\Omega_{y}{\Omega_{z}}^{3}+696960\Omega_{y}{\Omega_{z}}^{2}+15072\Omega_{y}\Omega_{z}+120\Omega_{y}+174182400{\Omega_{z}}^{6}+51840000{\Omega_{z}}^{5}+}{1008000E^{3}Iy^{3}{\left(12\Omega_{y}+1\right)}^{3}{\left(12\Omega_{z}+1\right)}^{4}}\\ & & \frac{+7015680{\Omega_{z}}^{4}+599040{\Omega_{z}}^{3}+30000{\Omega_{z}}^{2}+632\Omega_{z}+5)}{1008000E^{3}Iy^{3}{\left(12\Omega_{y}+1\right)}^{3}{\left(12\Omega_{z}+1\right)}^{4}})Mx_{2}P^{3}\\ & & =K(11,12)=K(12,11) \end{array}$$

When the constant Ω is very small, the Timoshenko beam theory behaves as the Euler-Bernoulli beam theory, and the tangent stiffness matrix is equal for both theories. However, expressions for the tangent stiffness matrix considering just the Euler-Bernoulli beam theory can be found in Rodrigues [17] and the open-source code in [15] and [16].

## Verification

**Fig. 1:**
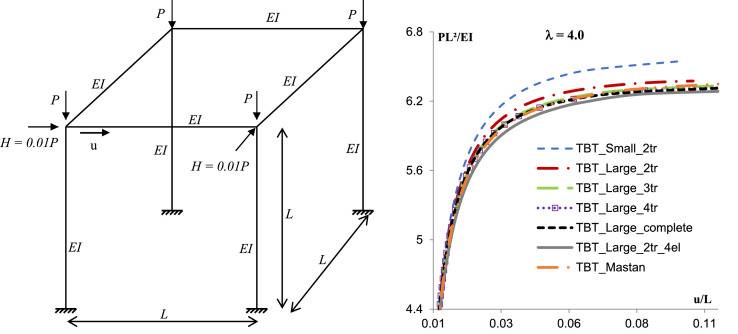
Spatial frame with torsion.

**Fig. 2:**
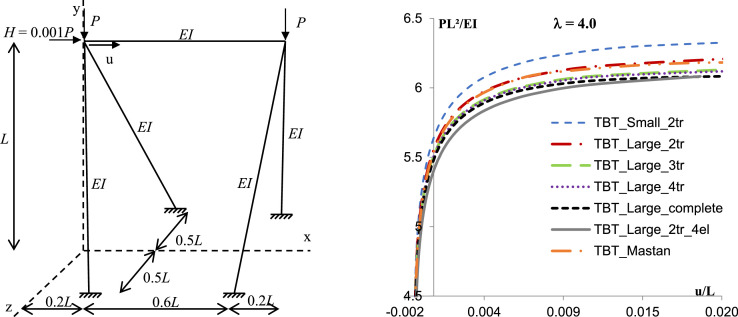
Spatial frame with inclined columns (adapted from Zugic et al. [19]).

**Fig. 3:**
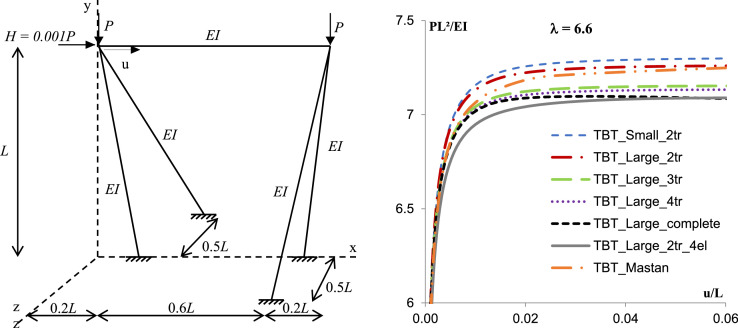
Asymmetric spatial frame (adapted from Zugic et al. [19]).

To verify the developed tangent stiffness matrix, the buckling of spatial frames was studied, adopting just one element per member, and with a moderate slenderness ratio (λ=L/h) to consider the Timoshenko beam theory influence. The responses were compared with a numerical solution considering a discretized structure using the usual formulation. Studied examples have length *L* = 1 m, Young's modulus *E* = 10^7^ kN/m^2^, a section form factor χ=5/6 and a Poisson's ratio ν=0.3. Equilibrium paths found for the structures are given Fig. 1, Fig. 2, Fig. 3, and the reference solution for comparison purposes is given by a structure with 4 segments in each member (TBT_Large_2tr_4el) Table 1.

**Table 1:** Summary results.

Discretization	1 Element						4 Elements
Element - TBT	Mastan	Small	Large				
		2tr	2tr	3tr	4tr	complete	2tr_4el
Spatial frame with torsion − λ = 4.0	6.389	6.548	6.374	6.367	**6.367**	**6.341**	**6.341**
Spatial frame with inclined columns − λ = 4.0	6.182	6.371	6.227	6.155	**6.155**	**6.082**	**6.082**
Asymmetric spatial frame − λ = 6.6	7.282	7.298	7.259	7.152	**7.132**	**7.083**	**7.089**

The examples clearly evidence the efficiency of the proposed method to predict the critical buckling load for the spatial frame with no discretization and with torsion interaction. For the spatial frame with torsion and the asymmetric spatial frame, results considering the complete formulation or the Taylor series expansion with 4 or 3 terms, and using just one element in each member provides differences with the response of the discretized structure using conventional formulation in order of 0,5%.

**Fig. 4:**
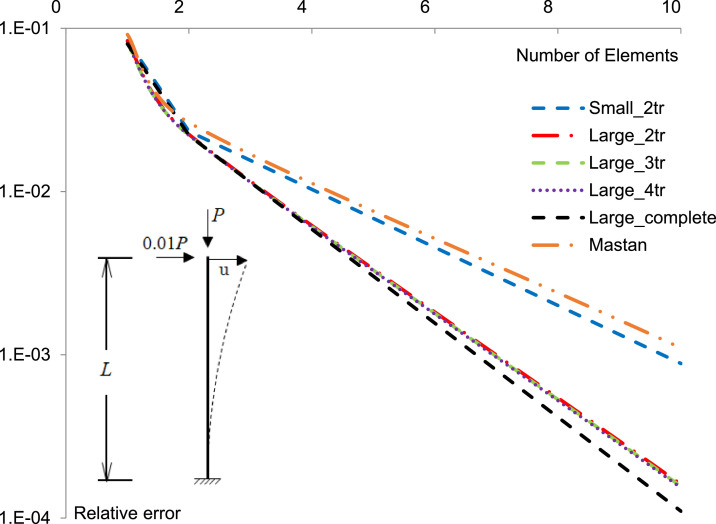
Convergence rate for the horizontal displacement of a clamped beam-column.

In order to check the performance of the stiffness matrices presented in this paper, a convergence study was implemented. Fig. 4 presents the convergence rate found considering different options for the tangent stiffness matrix. The study was carried out by comparing the top displacement of a beam-column to the one obtained by a highly discretized solution using conventional elements. The beam-column was loaded according to Fig. 4, with *P* = 0.6 Pcr (60% of the critical buckling load).

The presented method can be used to perform a nonlinear geometric analysis of spatial structures using minimal or a reduced discretization, whereas, while the usual tangent stiffness matrix would require a more refined mesh to predict the structure behavior. The method can be implemented in any structural analysis software. Matrix coefficients use hyperbolic and trigonometric expressions. Alternatively, polynomial expressions for geometric stiffness coefficients using a Taylor series expansion with 4 or 3 terms are proposed, and all necessary equations are available online in open source.

## Analytical interpolation functions

The presented tangent stiffness was calculated by using interpolation functions for displacements and rotations in a frame element obtained directly from the solution of differential equations that consider the equilibrium in the deformed configuration. As a consequence, these interpolation functions depend on the axial force in the element, and also on the parameters of the Timoshenko beam theory. Moreover, the interpolation functions deduced in the deformed configuration can be used to improve visualization in a graphics program and the computation of the local P-delta effect using just one element per member.

For a tensile force (*P* > 0), the interpolation functions for displacements and rotations are written in terms of hyperbolic functions, according to:

(94) $$\begin{array}{c} N_{2}^{v}=-\frac{\cosh\left(L\Lambda \right)-\cosh\left(\Lambda x\right)+\cosh\left(\Lambda \left(L-x\right)\right)+L^{2}\Lambda^{2}\Omega -L\Lambda sinh\left(L\Lambda \right)}{L\Lambda sinh\left(L\Lambda \right)-2L^{2}\Lambda^{2}\Omega -2\cosh\left(L\Lambda \right)+2L^{2}\Lambda^{2}\Omega \cosh\left(L\Lambda \right)+2}+\\ +\frac{\Lambda xsinh\left(L\Lambda \right)-L^{2}\Lambda^{2}\Omega \cosh\left(L\Lambda \right)+L^{2}\Lambda^{2}\Omega \cosh\left(\Lambda x\right)-L^{2}\Lambda^{2}\Omega \cosh\left(\Lambda \left(L-x\right)\right)-1}{L\Lambda sinh\left(L\Lambda \right)-2L^{2}\Lambda^{2}\Omega -2\cosh\left(L\Lambda \right)+2L^{2}\Lambda^{2}\Omega \cosh\left(L\Lambda \right)+2} \end{array}$$

(95) $$\begin{array}{c} N_{3}^{v}=\frac{\left(L^{2}\Lambda^{2}\Omega -1\right)\left(sinh\left(L\Lambda \right)-sinh\left(\Lambda x\right)-sinh\left(\Lambda \left(L-x\right)\right)-\Lambda x-L\Lambda cosh\left(L\Lambda \right)\right)}{\Lambda \left(L\Lambda sinh\left(L\Lambda \right)-2L^{2}\Lambda^{2}\Omega -2\cosh\left(L\Lambda \right)+2L^{2}\Lambda^{2}\Omega \cosh\left(L\Lambda \right)+2\right)}+\\ +\frac{\left(L^{2}\Lambda^{2}\Omega -1\right)\left(\Lambda xcosh\left(L\Lambda \right)+L\Lambda cosh\left(\Lambda \left(L-x\right)\right)-L^{2}\Lambda^{2}\Omega sinh\left(L\Lambda \right)\right)}{\Lambda \left(L\Lambda sinh\left(L\Lambda \right)-2L^{2}\Lambda^{2}\Omega -2\cosh\left(L\Lambda \right)+2L^{2}\Lambda^{2}\Omega \cosh\left(L\Lambda \right)+2\right)}+\\ +\frac{\left(L^{2}\Lambda^{2}\Omega -1\right)\left(L^{2}\Lambda^{2}\Omega sinh\left(\Lambda x\right)+L^{2}\Lambda^{2}\Omega sinh\left(\Lambda \left(L-x\right)\right)\right)}{\Lambda \left(L\Lambda sinh\left(L\Lambda \right)-2L^{2}\Lambda^{2}\Omega -2\cosh\left(L\Lambda \right)+2L^{2}\Lambda^{2}\Omega \cosh\left(L\Lambda \right)+2\right)} \end{array}$$

(96) $$\begin{array}{c} N_{5}^{v}=\frac{\cosh\left(\Lambda \left(L-x\right)\right)-\cosh\left(\Lambda x\right)-\cosh\left(L\Lambda \right)-L^{2}\Lambda^{2}\Omega +\Lambda xsinh\left(L\Lambda \right)}{L\Lambda sinh\left(L\Lambda \right)-2L^{2}\Lambda^{2}\Omega -2\cosh\left(L\Lambda \right)+2L^{2}\Lambda^{2}\Omega \cosh\left(L\Lambda \right)+2}+\\ +\frac{L^{2}\Lambda^{2}\Omega \cosh\left(L\Lambda \right)+L^{2}\Lambda^{2}\Omega \cosh\left(\Lambda x\right)-L^{2}\Lambda^{2}\Omega \cosh\left(\Lambda \left(L-x\right)\right)-1}{L\Lambda sinh\left(L\Lambda \right)-2L^{2}\Lambda^{2}\Omega -2\cosh\left(L\Lambda \right)+2L^{2}\Lambda^{2}\Omega \cosh\left(L\Lambda \right)+2} \end{array}$$

(97) $$\begin{array}{c} N_{6}^{v}=\frac{-\left(L^{2}\Lambda^{2}\Omega -1\right)\left(sinh\left(L\Lambda \right)-sinh\left(\Lambda x\right)-sinh\left(\Lambda \left(L-x\right)\right)+\Lambda x+L\Lambda \right)}{\Lambda \left(L\Lambda sinh\left(L\Lambda \right)-2L^{2}\Lambda^{2}\Omega -2\cosh\left(L\Lambda \right)+2L^{2}\Lambda^{2}\Omega \cosh\left(L\Lambda \right)+2\right)}+\\ +\frac{-\left(L^{2}\Lambda^{2}\Omega -1\right)\left(L\Lambda cosh\left(\Lambda x\right)-\Lambda xcosh\left(L\Lambda \right)-L^{2}\Lambda^{2}\Omega sinh\left(L\Lambda \right)\right)}{\Lambda \left(L\Lambda sinh\left(L\Lambda \right)-2L^{2}\Lambda^{2}\Omega -2\cosh\left(L\Lambda \right)+2L^{2}\Lambda^{2}\Omega \cosh\left(L\Lambda \right)+2\right)}+\\ +\frac{-\left(L^{2}\Lambda^{2}\Omega -1\right)\left(L^{2}\Lambda^{2}\Omega sinh\left(\Lambda x\right)+L^{2}\Lambda^{2}\Omega sinh\left(\Lambda \left(L-x\right)\right)\right)}{\Lambda \left(L\Lambda sinh\left(L\Lambda \right)-2L^{2}\Lambda^{2}\Omega -2\cosh\left(L\Lambda \right)+2L^{2}\Lambda^{2}\Omega \cosh\left(L\Lambda \right)+2\right)} \end{array}$$

(98) $$N_{2}^{\theta }=\frac{\Lambda \left(sinh\left(\Lambda x\right)-sinh\left(L\Lambda \right)+\sinh\left(\Lambda \left(L-x\right)\right)\right)}{L\Lambda sinh\left(L\Lambda \right)-2L^{2}\Lambda^{2}\Omega -2\cosh\left(L\Lambda \right)+2L^{2}\Lambda^{2}\Omega \cosh\left(L\Lambda \right)+2}$$

(99) $$\begin{array}{ccc} N_{3}^{\theta } & = & \frac{\cosh\left(\Lambda x\right)-\cosh\left(L\Lambda \right)-\cosh\left(\Lambda \left(L-x\right)\right)-L^{2}\Lambda^{2}\Omega +L\Lambda \sinh\left(\Lambda \left(L-x\right)\right)}{L\Lambda \sinh\left(L\Lambda \right)-2L^{2}\Lambda^{2}\Omega -2\cosh\left(L\Lambda \right)+2L^{2}\Lambda^{2}\Omega \cosh\left(L\Lambda \right)+2}+\\ & & +\;\frac{L^{2}\Lambda^{2}\Omega \cosh\left(L\Lambda \right)-L^{2}\Lambda^{2}\Omega \cosh\left(\Lambda x\right)+L^{2}\Lambda^{2}\Omega \cosh\left(\Lambda \left(L-x\right)\right)+1}{L\Lambda \sinh\left(L\Lambda \right)-2L^{2}\Lambda^{2}\Omega -2\cosh\left(L\Lambda \right)+2L^{2}\Lambda^{2}\Omega \cosh\left(L\Lambda \right)+2} \end{array}$$

(100) $$N_{5}^{\theta }=-\frac{\Lambda \left(sinh\left(\Lambda x\right)-sinh\left(L\Lambda \right)+\sinh\left(\Lambda \left(L-x\right)\right)\right)}{L\Lambda sinh\left(L\Lambda \right)-2L^{2}\Lambda^{2}\Omega -2\cosh\left(L\Lambda \right)+2L^{2}\Lambda^{2}\Omega \cosh\left(L\Lambda \right)+2}$$

(101) $$\begin{array}{ccc} N_{6}^{\theta } & = & \frac{\cosh\left(\Lambda \left(L-x\right)\right)-\cosh\left(\Lambda x\right)-\cosh\left(L\Lambda \right)-L^{2}\Lambda^{2}\Omega +L\Lambda \sinh\left(\Lambda x\right)}{L\Lambda \sinh\left(L\Lambda \right)-2L^{2}\Lambda^{2}\Omega -2\cosh\left(L\Lambda \right)+2L^{2}\Lambda^{2}\Omega \cosh\left(L\Lambda \right)+2}+\\ & & +\;\frac{L^{2}\Lambda^{2}\Omega \cosh\left(L\Lambda \right)+L^{2}\Lambda^{2}\Omega \cosh\left(\Lambda x\right)-L^{2}\Lambda^{2}\Omega \cosh\left(\Lambda \left(L-x\right)\right)+1}{L\Lambda \sinh\left(L\Lambda \right)-2L^{2}\Lambda^{2}\Omega -2\cosh\left(L\Lambda \right)+2L^{2}\Lambda^{2}\Omega \cosh\left(L\Lambda \right)+2} \end{array}$$

For a compressive force (*P* < 0), the interpolation functions for displacements and rotations are written in terms of trigonometric functions:

(101) $$\begin{array}{ccc} N_{2}^{v} & = & \frac{\cos\left(L\Lambda \right)-\cos\left(\Lambda x\right)+\cos\left(\Lambda \left(L-x\right)\right)-L^{2}\Lambda^{2}\Omega +L\Lambda sin\left(L\Lambda \right)-\Lambda xsin\left(L\Lambda \right)}{2\cos\left(L\Lambda \right)-2L^{2}\Lambda^{2}\Omega +L\Lambda sin\left(L\Lambda \right)+2L^{2}\Lambda^{2}\Omega \cos\left(L\Lambda \right)-2}+\\ & & +\;\frac{L^{2}\Lambda^{2}\Omega \cos\left(L\Lambda \right)-L^{2}\Lambda^{2}\Omega \cos\left(\Lambda x\right)+L^{2}\Lambda^{2}\Omega \cos\left(\Lambda \left(L-x\right)\right)-1}{2\cos\left(L\Lambda \right)-2L^{2}\Lambda^{2}\Omega +L\Lambda sin\left(L\Lambda \right)+2L^{2}\Lambda^{2}\Omega \cos\left(L\Lambda \right)-2} \end{array}$$

(103) $$\begin{array}{ccc} N_{3}^{v} & = & \frac{-\left(1+L^{2}\Lambda^{2}\Omega \right)\left(sin\left(\Lambda x\right)-sin\left(L\Lambda \right)+\sin\left(\Lambda \left(L-x\right)\right)+\Lambda x+L\Lambda cos\left(L\Lambda \right)\right)}{\Lambda \left(2\cos\left(L\Lambda \right)-2L^{2}\Lambda^{2}\Omega +L\Lambda sin\left(L\Lambda \right)+2L^{2}\Lambda^{2}\Omega \cos\left(L\Lambda \right)-2\right)}+\\ & & +\;\frac{-\left(1+L^{2}\Lambda^{2}\Omega \right)\left(-\Lambda xcos\left(L\Lambda \right)-L\Lambda \cos\left(\Lambda \left(L-x\right)\right)-L^{2}\Lambda^{2}\Omega sin\left(L\Lambda \right)\right)}{\Lambda \left(2\cos\left(L\Lambda \right)-2L^{2}\Lambda^{2}\Omega +L\Lambda sin\left(L\Lambda \right)+2L^{2}\Lambda^{2}\Omega \cos\left(L\Lambda \right)-2\right)}\\ & & +\;\frac{-\left(1+L^{2}\Lambda^{2}\Omega \right)\left(L^{2}\Lambda^{2}\Omega sin\left(\Lambda x\right)+L^{2}\Lambda^{2}\Omega \sin\left(\Lambda \left(L-x\right)\right)\right)}{\Lambda \left(2\cos\left(L\Lambda \right)-2L^{2}\Lambda^{2}\Omega +L\Lambda sin\left(L\Lambda \right)+2L^{2}\Lambda^{2}\Omega \cos\left(L\Lambda \right)-2\right)} \end{array}$$

(104) $$\begin{array}{ccc} N_{5}^{v} & = & \frac{\cos\left(L\Lambda \right)+\cos\left(\Lambda x\right)-\cos\left(\Lambda \left(L-x\right)\right)-L^{2}\Lambda^{2}\Omega +\Lambda xsin\left(L\Lambda \right)+}{2\cos\left(L\Lambda \right)-2L^{2}\Lambda^{2}\Omega +L\Lambda sin\left(L\Lambda \right)+2L^{2}\Lambda^{2}\Omega \cos\left(L\Lambda \right)-2}+\\ & & +\;\frac{L^{2}\Lambda^{2}\Omega \cos\left(L\Lambda \right)+L^{2}\Lambda^{2}\Omega \cos\left(\Lambda x\right)-L^{2}\Lambda^{2}\Omega \cos\left(\Lambda \left(L-x\right)\right)-1}{2\cos\left(L\Lambda \right)-2L^{2}\Lambda^{2}\Omega +L\Lambda sin\left(L\Lambda \right)+2L^{2}\Lambda^{2}\Omega \cos\left(L\Lambda \right)-2} \end{array}$$

(105) $$\begin{array}{ccc} N_{6}^{v} & = & \frac{\left(1+L^{2}\Lambda^{2}\Omega \right)\left(sin\left(\Lambda x\right)-sin\left(L\Lambda \right)+\sin\left(\Lambda \left(L-x\right)\right)-\Lambda x+L\Lambda \right)}{\Lambda \left(2\cos\left(L\Lambda \right)-2L^{2}\Lambda^{2}\Omega +L\Lambda sin\left(L\Lambda \right)+2L^{2}\Lambda^{2}\Omega \cos\left(L\Lambda \right)-2\right)}+\\ & & +\;\frac{\left(1+L^{2}\Lambda^{2}\Omega \right)\left(-L\Lambda cos\left(\Lambda x\right)+\Lambda xcos\left(L\Lambda \right)-L^{2}\Lambda^{2}\Omega sin\left(L\Lambda \right)\right)}{\Lambda \left(2\cos\left(L\Lambda \right)-2L^{2}\Lambda^{2}\Omega +L\Lambda sin\left(L\Lambda \right)+2L^{2}\Lambda^{2}\Omega \cos\left(L\Lambda \right)-2\right)}\\ & & +\;\frac{\left(1+L^{2}\Lambda^{2}\Omega \right)\left(L^{2}\Lambda^{2}\Omega sin\left(\Lambda x\right)+L^{2}\Lambda^{2}\Omega \sin\left(\Lambda \left(L-x\right)\right)\right)}{\Lambda \left(2\cos\left(L\Lambda \right)-2L^{2}\Lambda^{2}\Omega +L\Lambda sin\left(L\Lambda \right)+2L^{2}\Lambda^{2}\Omega \cos\left(L\Lambda \right)-2\right)} \end{array}$$

(106) $$N_{2}^{\theta }=\frac{\Lambda \left(sin\left(\Lambda x\right)-sin\left(L\Lambda \right)+\sin\left(\Lambda \left(L-x\right)\right)\right)}{2\cos\left(L\Lambda \right)-2L^{2}\Lambda^{2}\Omega +L\Lambda sin\left(L\Lambda \right)+2L^{2}\Lambda^{2}\Omega \cos\left(L\Lambda \right)-2}$$

(107) $$\begin{array}{ccc} N_{3}^{\theta } & = & \frac{\cos\left(L\Lambda \right)-\cos\left(\Lambda x\right)+\cos\left(\Lambda \left(L-x\right)\right)-L^{2}\Lambda^{2}\Omega +L\Lambda sin\left(\Lambda \left(L-x\right)\right)}{2\cos\left(L\Lambda \right)-2L^{2}\Lambda^{2}\Omega +L\Lambda sin\left(L\Lambda \right)+2L^{2}\Lambda^{2}\Omega \cos\left(L\Lambda \right)-2}\\ & & +\;\frac{L^{2}\Lambda^{2}\Omega \cos\left(L\Lambda \right)-L^{2}\Lambda^{2}\Omega \cos\left(\Lambda x\right)+L^{2}\Lambda^{2}\Omega \cos\left(\Lambda \left(L-x\right)\right)-1}{2\cos\left(L\Lambda \right)-2L^{2}\Lambda^{2}\Omega +L\Lambda sin\left(L\Lambda \right)+2L^{2}\Lambda^{2}\Omega \cos\left(L\Lambda \right)-2} \end{array}$$

(108) $$N_{5}^{\theta }=-\frac{\Lambda \left(sin\left(\Lambda x\right)-sin\left(L\Lambda \right)+\sin\left(\Lambda \left(L-x\right)\right)\right)}{2\cos\left(L\Lambda \right)-2L^{2}\Lambda^{2}\Omega +L\Lambda sin\left(L\Lambda \right)+2L^{2}\Lambda^{2}\Omega \cos\left(L\Lambda \right)-2}$$

(109) $$\begin{array}{ccc} N_{6}^{\theta } & = & \frac{\cos\left(L\Lambda \right)+\cos\left(\Lambda x\right)-\cos\left(\Lambda \left(L-x\right)\right)-L^{2}\Lambda^{2}\Omega +L\Lambda sin\left(\Lambda x\right)}{2\cos\left(L\Lambda \right)-2L^{2}\Lambda^{2}\Omega +L\Lambda sin\left(L\Lambda \right)+2L^{2}\Lambda^{2}\Omega \cos\left(L\Lambda \right)-2}\\ & & +\;\frac{L^{2}\Lambda^{2}\Omega \cos\left(L\Lambda \right)+L^{2}\Lambda^{2}\Omega \cos\left(\Lambda x\right)-L^{2}\Lambda^{2}\Omega \cos\left(\Lambda \left(L-x\right)\right)-1}{2\cos\left(L\Lambda \right)-2L^{2}\Lambda^{2}\Omega +L\Lambda sin\left(L\Lambda \right)+2L^{2}\Lambda^{2}\Omega \cos\left(L\Lambda \right)-2} \end{array}$$

Rodrigues [17] also presents analytical interpolation functions for displacements and rotations considering exponential functions along with expressions for articulated left member end, and for articulated right member end. In cases for which the constantΩ is very small, the Timoshenko beam theory behaves as the Euler-Bernoulli beam theory and the interpolation functions lead to the same results. Expressions for the interpolation functions considering just the Euler-Bernoulli beam theory are also featured in [17] and implemented in [15] and [16].

**Fig. 5:**
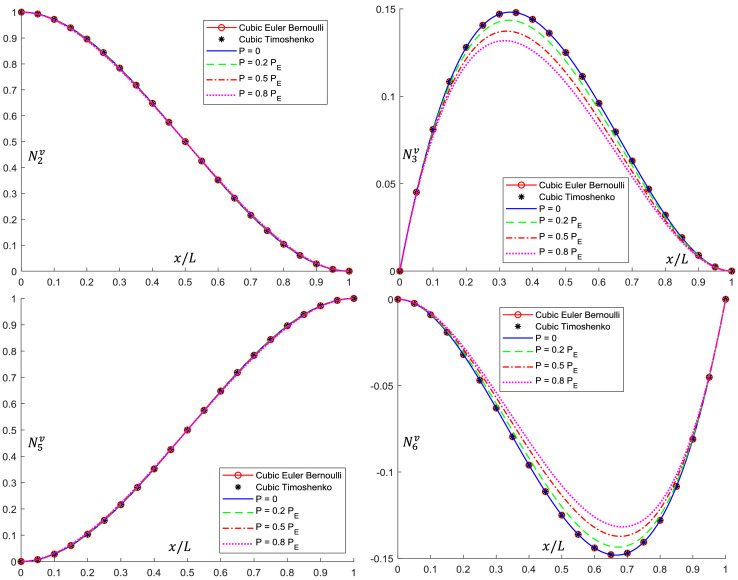
Translational interpolation functions for variable axial load *P* and Ω=0.

**Fig. 6:**
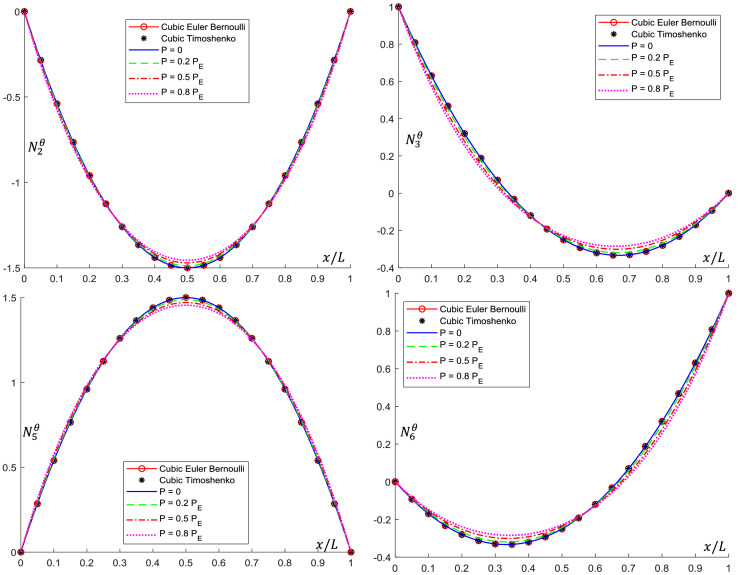
Rotational interpolation functions for variable axial load *P* and Ω=0.

**Fig. 7:**
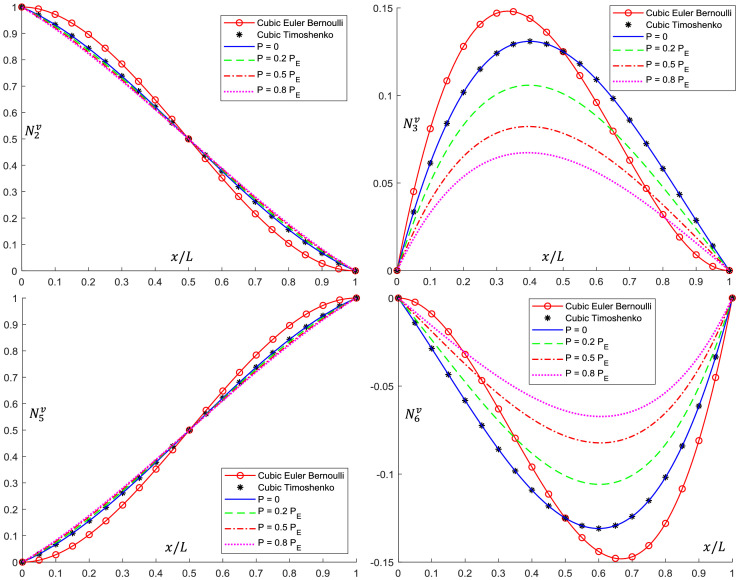
Translational interpolation functions for variable axial load *P* and Ω=0.1.

**Fig. 8:**
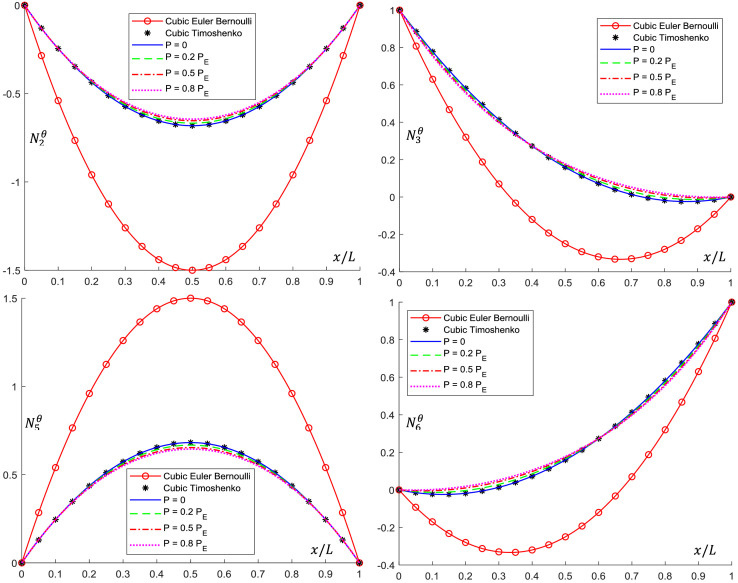
Rotational interpolation functions for variable axial load *P* and Ω=0.1.

**Fig. 9:**
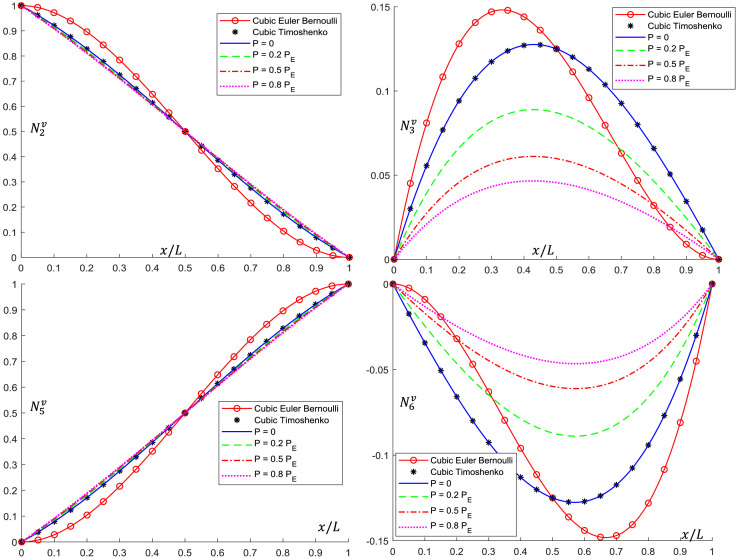
Translational interpolation functions for variable axial load *P* and Ω=0.2.

**Fig. 10:**
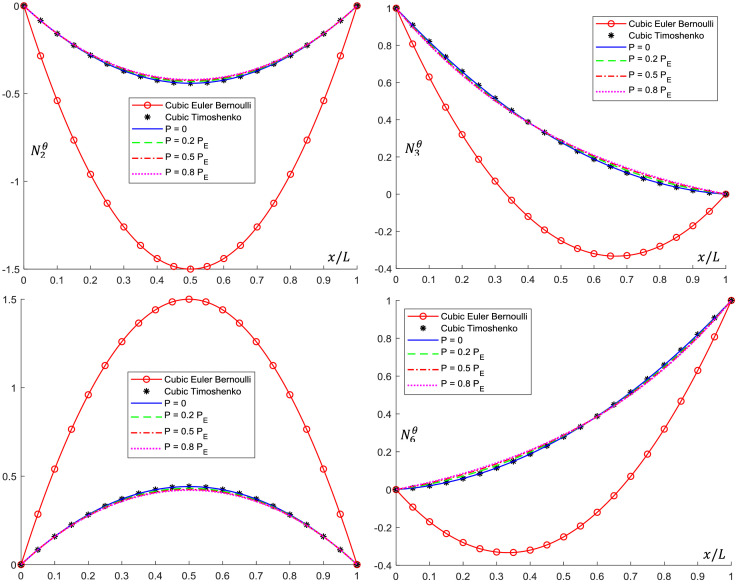
Rotational interpolation functions for variable axial load *P* and Ω=0.2.

Analytical interpolation functions plots are shown in Fig. 5, Fig. 6, Fig. 7, Fig. 8, Fig. 9, Fig. 10. It can be noticed that when the axial load is null (*P = 0*), conventional cubic interpolation functions are identical to the ones developed in this work. As the axial load increases, analytical interpolations functions present a distinct behavior, as expected. This influence becomes more evident for elements with Ω>0, for which the Timoshenko beam theory is used.

## Declaration of Competing Interest

The authors declare that they have no known competing financial interests or personal relationships that could have appeared to influence the work reported in this paper.
